# Protein Disulfide Isomerase Disassembles TDP‐43/G3BP1 Condensates and Antagonizes TDP‐43 Pathological Aggregates

**DOI:** 10.1002/advs.202516846

**Published:** 2026-05-25

**Authors:** Jia‐Qi Liu, Hao Liu, Yu‐Xuan Sun, Yuying Li, Xiangyi Liu, Li‐Qiang Wang, Zhaofei Yang, Qi Fu, Xiaojiao Xu, Jie Chen, Yingshuang Zhang, Jun Zhou, Weidong Le, Mengchao Cui, Yi Liang

**Affiliations:** ^1^ Hubei Key Laboratory of Cell Homeostasis College of Life Sciences Wuhan University Wuhan China; ^2^ Key Laboratory of Radiopharmaceuticals Ministry of Education College of Chemistry Beijing Normal University Beijing China; ^3^ Department of Neurology Peking University Third Hospital Beijing China; ^4^ Liaoning Provincial Key Laboratory for Research on the Pathogenic Mechanisms of Neurological Diseases the First Affiliated Hospital Dalian Medical University Dalian China; ^5^ Hubei Key Laboratory of Bioinorganic Chemistry & Materia Medica Hubei Engineering Research Center for Biomaterials and Medical Protective Materials School of Chemistry and Chemical Engineering Huazhong University of Science and Technology Wuhan China; ^6^ Institute of Neurology, Sichuan Academy of Medical Sciences‐Sichuan Provincial Hospital Medical School of University of Electronic Science and Technology of China Chengdu China; ^7^ Center For Advanced Materials Research & Faculty of Arts and Sciences Beijing Normal University At Zhuhai Zhuhai China

**Keywords:** amyotrophic lateral sclerosis, mitochondrial impairment, protein aggregation, protein disulfide isomerase, protein phase separation, TDP‐43

## Abstract

Cytoplasmic mislocalization and aggregation of transactive response DNA‐binding protein‐43 (TDP‐43) is a common pathological feature of amyotrophic lateral sclerosis (ALS), frontotemporal lobar degeneration, and Alzheimer's disease with TDP‐43 pathology (AD‐TDP); the exact role of protein disulfide isomerase (PDI), an enzyme with chaperone activity, in modulating the pathological behavior of TDP‐43 is unknown. In this study, we report that wild‐type PDI, through its specific interaction with TDP‐43, markedly attenuates phase separation of TDP‐43, competitively displaces G3BP1 to disassemble TDP‐43/G3BP1 condensates, and further counteracts the pathological mislocalization, abnormal phosphorylation, and pathological aggregation of TDP‐43 through the b’ domain of the enzyme. Ultimately, this alleviates mitochondrial damage and neuronal toxicity caused by TDP‐43 aggregation and suppresses *UNC13A* cryptic splicing in stressed cells. In the presence of abnormal forms of PDI, however, PDI loses its activity, and stress granules containing TDP‐43 are assembled into amyloid fibrils, resulting in mitochondrial impairment and neuronal cell death in ALS and AD‐TDP patients. These findings not only provide new insights into the pathogenic mechanisms of TDP‐43 in neurodegenerative diseases such as ALS and AD‐TDP, but also propose PDI as a potential therapeutic target.

## Graphical abstract

Cytoplasmic aggregation of TDP‐43 is a common pathological feature in amyotrophic lateral sclerosis, frontotemporal lobar degeneration, and Alzheimer's disease with TDP‐43 pathology. This study reports that wild‐type PDI slows down phase separation of TDP‐43 through direct interaction with TDP‐43. Wild‐type PDI decreases oxidative stress‐induced interaction between TDP‐43 and G3BP1 to disassemble stress granules containing TDP‐43, and counteracts TDP‐43 pathological phosphorylation.

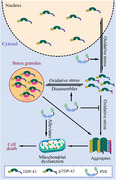

**Abbreviations**
AD: Alzheimer's disease
ALS: amyotrophic lateral sclerosis
BiFC: bimolecular fluorescence complementation
BSA: bovine serum albumin
co‐IP: coimmunoprecipitation
dnPDI: dominant‐negative PDI
ER: endoplasmic reticulum
FRAP: fluorescence recovery after photobleaching
FTLD: frontotemporal lobar degeneration
IP: Immunoprecipitation
PDI: protein disulfide isomerase;
PSP: progressive supranuclear palsy.
SNO‐PDI: S‐nitrosylated PDI
SOD1: copper–zinc superoxide dismutase
TAMRA: 5(6)‐carboxy‐tetramethylrhodamine N‐succinimidyl ester
TDP‐43: transactive response DNA‐binding protein‐43
TEM: transmission electron microscopy

## 1 Introduction

Transactive response DNA‐binding protein‐43 (TDP‐43), a highly conserved, ubiquitously expressed nuclear DNA/RNA‐binding protein, has multifaceted functions in regulating RNA metabolism, alternative splicing, miRNA processing, and translation of mRNA [1]. Under pathological stress conditions, TDP‐43 is partly mislocalized from the nucleus to the cytosol and is pathologically phosphorylated; cytoplasmic mislocalization and aggregation of TDP‐43 in neurons are common pathological features in amyotrophic lateral sclerosis (ALS) and frontotemporal lobar degeneration (FTLD‐TDP) [1, 2, 3, 4, 5, 6]. ALS is an invariably fatal neurodegenerative disease characterized by the selective death of motor neurons in the brain and spinal cord in a progressive manner [1, 7, 8]. Cytoplasmic accumulation of copper–zinc superoxide dismutase (SOD1) in motor neurons is another pathological feature of ALS [7, 8, 9]. Cytoplasmic accumulation of TDP‐43 is also observed in the brain of some patients with Alzheimer's disease (AD) [10, 11, 12, 13, 14, 15, 16, 17, 18] or AD transgenic mice [14], but the mechanism behind the phenomenon remains unclear.

Protein disulfide isomerase (PDI) functions as both an enzyme and a molecular chaperone to catalyze disulfide bond formation, isomerization, and reduction under physiological conditions and to correct or eliminate misfolded proteins under pathological conditions [19, 20, 21, 22, 23, 24, 25]. PDI is mainly located at the endoplasmic reticulum (ER) of cells; under ER stress conditions, this ER‐resident protein is partly mislocalized from the ER to the cytosol, named PDI ER‐to‐cytosol reflux [22, 24, 26, 27, 28, 29, 30]. PDI protects against protein aggregation and is S‐nitrosylated in several diverse neurodegenerative diseases, ranging from AD to ALS and Parkinson's disease [21, 22, 23, 24, 25]. Another member of the PDI family, ERp57, has been reported to inhibit nuclear export and aggregation of the TDP‐43 M337V mutant [31]. However, the exact role of PDI in neurodegenerative diseases is unknown.

RNA‐binding proteins such as TDP‐43 tend to form supramolecular assemblies called membrane‐less organelles, like stress granules via phase separation of proteins in cells to perform key functions [32, 33, 34, 35]. The liquid droplets formed by biological macromolecules, called biomolecular condensates, have fusion properties [33, 36, 37, 38, 39, 40, 41, 42]. Because TDP‐43 contains a low‐complexity domain in its C‐terminal domain [6, 38, 39, 40, 41, 43, 44, 45], it undergoes phase separation in vitro and in cells and forms TDP‐43 condensates [37, 38, 39, 40, 41, 43, 44, 45, 46, 47]. TDP‐43 liquid‐phase condensation is modulated by molecular chaperones (Hsp70 and HSPB1), pathological phosphorylation, pathological mutations, RNA (mRNA and yeast total RNA), and other factors [37, 38, 39, 40, 41, 43, 44, 45, 46, 47]. Very recently, Yan and co‐workers reported that TDP‐43 condensates are assembled into TDP‐43 pathological aggregates [48]. In contrast, Zhang and co‐workers reported that YAP modulates the dynamics of TDP‐43 condensates to antagonize TDP‐43 pathological aggregates [49]. However, it is unclear whether TDP‐43 selectively recruits PDI into phase‐separated condensates, which in turn regulates phase separation of TDP‐43.

In this study, we systematically revealed the multifaceted roles of wild‐type PDI in regulating the pathological processes of TDP‐43. Specifically, we focused on the effects of the interaction between cytoplasmic TDP‐43 and wild‐type PDI with the connections between cytoplasmic TDP‐43 and a key stress granule component, G3BP1. We unmasked the role of wild‐type PDI in disassembling TDP‐43/G3BP1 condensates by weakening the interaction between cytoplasmic TDP‐43 and G3BP1 under pathological stress conditions. Our findings provide insights into the regulation of PDI on pathological functions of cytoplasmic TDP‐43 via phase separation of the protein inhibited by wild‐type PDI, but not abnormal forms of PDI, which has important implications in ALS etiology.

## 2 Results

### 2.1 TDP‐43 Directly Interacts With PDI Through the *b*′ Domain of the Enzyme

**FIGURE 1:**
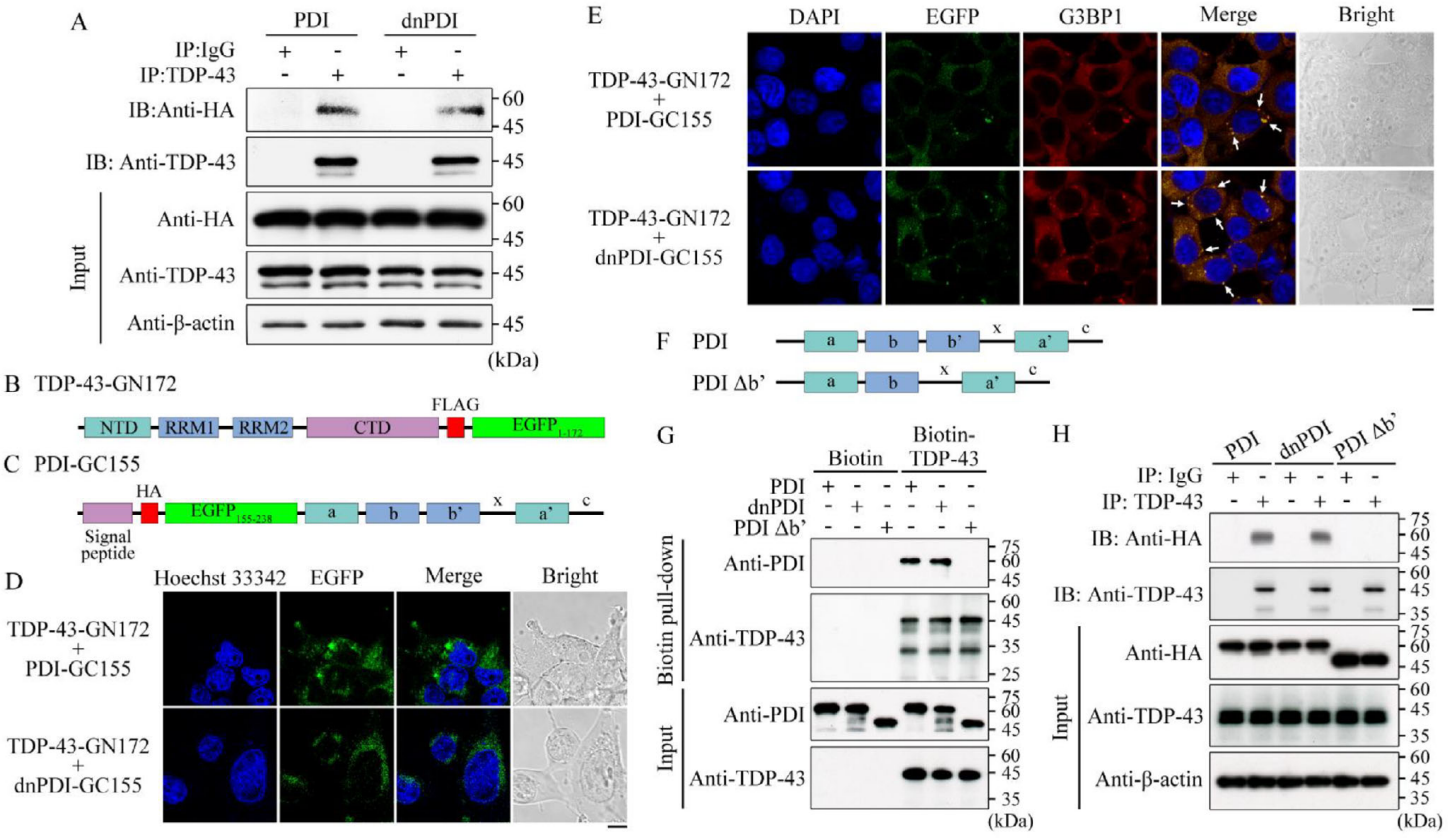
TDP‐43 directly interacts with PDI through the *b′* domain of the enzyme. (A) Co‐IP assay to verify the interaction of TDP‐43 with PDI in HEK‐293T cells transiently co‐transfected with TDP‐43 and HA‐tagged wild‐type PDI or HA‐tagged dnPDI treated with 1 mm H_2_O_2_ for 1 h. (B,C) Schemes of TDP‐43‐PDI constructs used in BiFC assay: TDP‐43‐EGFP_1‐172_ fusion protein (B) and signal peptide‐HA‐EGFP_155‐238_‐*a*‐*b*‐*b′*‐*x*‐*a′*‐*c* fusion protein (C). (D) Specific interaction between TDP‐43 and PDI in living cells was detected by BiFC. HEK‐293T cells transiently expressing both TDP‐43‐GN172 and wild‐type PDI‐GC155 or dnPDI‐GC155 constructs were cultured for 1 day and treated with 1 mm H_2_O_2_ for 1 h. Shown are nuclei stained with Hoechst 33342 (blue). EGFP (green) was observed in (D). The negative controls of BiFC experiments are shown in Figure S1B–G, and the vector‐only control is shown in Figure S1H,I. (E) Immunofluorescence images of HEK‐293T cells transiently expressing both TDP‐43‐GN172 and wild‐type PDI‐GC155 or dnPDI‐GC155 constructs cultured for 1 day and treated with 1 mm H_2_O_2_ for 1 h, using the anti‐G3BP1 (red) antibody and staining with DAPI (blue). EGFP (green) was observed in (E). The white arrows indicate colocalization of TDP‐43 condensates and endogenous G3BP1 in HEK‐293T cells. Scale bars, 10 µm (D,E). (F) Schemes of PDI (*a*‐*b*‐*b*′‐*x*‐a′‐*c*) and PDI Δ*b*′ (*a*‐*b*‐*x*‐*a*′‐*c*) constructs used in pull‐down assay and co‐IP assay. (G) Biotin pull‐down assay to map the binding region of PDI for the TDP‐43‐PDI complex. Wild‐type PDI, dnPDI, or PDI Δ*b′* mutant incubated with biotin or biotin‐labeled TDP‐43, followed by pull‐down with streptavidin beads. The interaction was determined by western blotting using anti‐PDI and anti‐TDP‐43 antibodies. (H) Co‐IP assay to map the binding region of PDI for the endogenous TDP‐43‐PDI complex in N2a cells transiently transfected with HA‐tagged wild‐type PDI, HA‐tagged dnPDI, or HA‐tagged PDI Δ*b′* treated with 1 mm H_2_O_2_ for 1 h. (A,H) Anti‐TDP‐43 antibody‐binding beads were used for co‐IP experiments and then detected by western blotting using anti‐HA, anti‐TDP‐43, and anti‐β‐actin antibodies. We have added IP bands of TDP‐43 in the second row.

Given that PDI protects against protein aggregation in neurodegenerative diseases, including ALS [21, 22, 23, 24, 25], we hypothesized that PDI might specifically interact with TDP‐43 in the cytoplasm of cells under pathological stress conditions. To test this hypothesis, we performed coimmunoprecipitation (co‐IP) experiments (Figure 1A) and took live‐cell images of TDP‐43‐PDI complex formation (Figure 1B–D). Our co‐IP experiments, visualized by the anti‐TDP‐43 antibody and anti‐HA antibody, verified the interaction of TDP‐43 with wild‐type PDI or dominant‐negative PDI (dnPDI), a quadruple cysteine mutant of this enzyme [21, 23], in HEK‐293T cells transiently co‐transfected with TDP‐43 and HA‐tagged wild‐type PDI or HA‐tagged dnPDI treated with 1 mm hydrogen peroxide (H_2_O_2_) for 1 h (Figure 1A). Here, H_2_O_2_ at a high concentration (1 mm) was used to induce oxidative stress and ER stress [50, 51, 52]. In contrast, our control co‐IP experiments verified that TDP‐43 did not bind to PDI in HEK‐293T cells transiently co‐transfected with TDP‐43 and HA‐tagged wild‐type PDI or dnPDI treated with PBS (Figure S1A). We then applied a bimolecular fluorescence complementation (BiFC) assay [53, 54] to determine the location of TDP‐43‐PDI interactions in living cells (Figure 1B–D). Schemes of TDP‐43‐PDI constructs were used in our BiFC assay: TDP‐43‐EGFP_1‐172_ fusion protein (Figure 1B) and signal peptide‐HA‐EGFP_155‐238_‐PDI fusion protein (Figure 1C). HEK‐293T cells transiently expressing both TDP‐43‐GN172 and wild‐type PDI‐GC155 or dnPDI‐GC155 constructs were cultured for 1 day and treated with 1 mm H_2_O_2_ for 1 h. Specific interactions between TDP‐43 and wild‐type PDI or dnPDI in the cytoplasm of living cells under pathological stress (H_2_O_2_‐induced oxidative stress) conditions were visualized by our BiFC experiments, and EGFP foci (green) were observed (Figure 1D), indicating that TDP‐43 interacts with PDI in the cytoplasm. In contrast, EGFP (green) was not detected in our negative control BiFC experiments (Figure S1B–G), indicating that neither the binding of TDP‐43 to PDI in living cells treated with PBS nor the interactions between TDP‐43‐GN172 and GC155, between GN172 and wild‐type PDI‐GC155, between GN172 and dnPDI‐GC155, or between GN172 and GC155 in living cells treated with 1 mm H_2_O_2_ for 1 h were detected by BiFC. It should be mentioned that the BiFC results in Figure S1B represent only a representative result, which shows that without 1 mm stimulation, TDP‐43 and PDI display almost no interaction. Similarly, EGFP (green) was not detected in the vector‐only control (Figure S1H,I). In cells treated with PBS, TDP‐43‐EGFP_1‐172_ fusion protein maintained its normal nuclear localization, indicating that the C‐terminal EGFP_1‐172 _tag cannot affect its subcellular localization (Figure S1H,I). In cells treated with 1 mM H_2_O_2_, however, the TDP‐43‐EGFP_1‐172_ fusion protein was partly mislocalized from the nucleus to the cytosol (Figure S1H,I). We asked whether TDP‐43‐PDI‐EGFP foci localize to stress granules under H_2_O_2_ stress. Notably, not only G3BP1‐positive stress granules (red dots) but also the colocalization of cytoplasmic TDP‐43‐PDI‐EGFP foci (green) and G3BP1 (red) were observed in HEK‐293T cells treated with 1 mM H_2_O_2_ (Figure 1E, yellow dots in the merged images), indicating that the TDP‐43‐PDI complex is potentially associated with stress granules under the pathological stress condition.

Given that wild‐type PDI specifically interacts with TDP‐43 in the cytoplasm of cells under pathological stress conditions (Figure 1A–D), we predicted that PDI might interact with TDP‐43 in the cytoplasm of the brain of ALS patients and AD patients with TDP‐43 pathology (AD‐TDP). To test this hypothesis, we took confocal images of paraffin brain sections from two ALS patients, six AD‐TDP cases, three healthy controls, and two controls with Castleman disease and progressive supranuclear palsy (PSP), respectively (Figures S2,S3 and Table 1). The ALS subtypes of the two ALS patients are TDP type B, and the regions stained are the precentral gyrus for ALS patient 1, and the precentral gyrus and the substantia nigra, respectively, for ALS patient 2. The brain sections were doubly immunostained with the anti‐TDP‐43 antibody (green) and anti‐PDI antibody (red) or with the anti‐ER antibody (green) and anti‐PDI antibody (red), stained with DAPI (blue), and visualized by confocal microscopy. TDP‐43 (green) was mislocalized to the cytoplasm; cytoplasmic TDP‐43 and PDI (red) signals exhibit overlapping distribution patterns in the brain of the ALS patients and the AD‐TDP patients (Figure S2A–H); and PDI was partly mislocalized from the ER to the cytosol in the brain of these patients (Figure S3A–H). In sharp contrast, TDP‐43 (green) neither mislocalized to the cytoplasm nor colocalized with PDI (red) in the brain of the five age‐matched controls (Figure S2I–M), and PDI was predominantly localized in the ER of the brain of the three healthy controls (yellow in the merged images, Figure S3I–K). The aforementioned findings were supported by the following statistical analysis. Pearson correlation coefficient (*r*) between TDP‐43 and PDI in the brain of ALS patients and AD‐TDP patients were 0.583 ± 0.049, 0.468 ± 0.015 or 0.488 ± 0.037, 0.512 ± 0.040, 0.461 ± 0.053, 0.500 ± 0.037, 0.478 ± 0.031, 0.486 ± 0.036, and 0.488 ± 0.032 for Cases 1, 2, 3, 4, 5, 6, 7, and 8, respectively, which was significantly higher than the Pearson *r*‐value between TDP‐43 and PDI in the brain of five controls (0.233 ± 0.058, 0.243 ± 0.087, 0.216 ± 0.076, 0.262 ± 0.039, and 0.249 ± 0.033 for Controls 1, 2, 3, 4, and 5, respectively) (Figure S2N). Pearson correlation coefficient (*r*) between ER and PDI in the brain of ALS patients and AD‐TDP patients were 0.605 ± 0.022, 0.578 ± 0.028, or 0.554 ± 0.041, 0.551 ± 0.050, 0.597 ± 0.050, 0.625 ± 0.026, 0.573 ± 0.039, 0.566 ± 0.049, and 0.614 ± 0.016 for Cases 1, 2, 3, 4, 5, 6, 7, and 8, respectively, which was significantly lower than the Pearson *r‐*value between ER and PDI in the brain of three healthy controls (0.724 ± 0.032, 0.740 ± 0.042, and 0.749 ± 0.048 for Controls 1, 2, and 3, respectively) (Figure S3L). Because fluorescent staining is prone to artifacts in human tissue, controls for non‐specific staining of ALS and AD‐TDP brains, as well as healthy, Castleman disease, and PSP controls were performed (the secondary antibody control), which all had very low levels of IgG (green and red) in most fields of view (Figure S4A–M). Thus, cytoplasmic TDP‐43 accumulated and exhibited overlapping distribution with possibly S‐nitrosylated PDI [21, 22] in the brain of ALS patients and AD‐TDP patients.

**TABLE 1:** Clinical details of patients with ALS and AD at the time when brain slices were taken.

Case	Gender	Age	Mean age ± SD	TDP‐43 pathology	Tau pathology	Amyloid‐β pathology	Clinical diagnosis
**ALS and Alzheimer's disease**							
1	M	69	76.9 ± 11.8	4‐5	ND	ND	Sporadic ALS
2	M	77		4	ND	ND	Sporadic ALS
3	M	76		ND	V	5	AD‐TDP
4	M	92		ND	V	4	AD‐TDP
5	M	95		ND	III	1	AD‐TDP
6	F	72		ND	I	1	AD‐TDP
7	M	59		ND	III	4	AD‐TDP
8	M	75		ND	V	6	AD‐TDP
**Control**							
1	M	76	74.8 ± 7.3	ND	0	0	Healthy individual
2	F	70		ND	0	0	Healthy individual
3	F	77		ND	0	0	Healthy individual
4	M	66		ND	0	0	Castleman disease
5	M	85		ND	II	0	PSP

**Abbreviations**: M, male; F, female; ND, not detected; TDP‐43 pathology (NCI/GCI), ALS, amyotrophic lateral sclerosis; AD‐TDP, Alzheimer's disease with TDP‐43 pathology; PSP, progressive supranuclear palsy.

Because the b’ domain of PDI provides a main binding site for substrate proteins [55, 56], we hypothesized that the interaction between TDP‐43 and PDI might be closely related to the b’ domain of the enzyme. To test this hypothesis, we performed a mapping analysis of the regions involved in TDP‐43 and PDI interaction. Schemes of PDI (*a*‐*b*‐*b*′‐*x*‐*a*′*‐c*) and PDI Δ*b*′ (*a‐b‐x‐a*′‐*c*) constructs (Figure 1F) were used in our pull‐down assay and co‐IP assay (Figure 1G,H). Wild‐type PDI, dnPDI, or PDI Δ*b*′ mutant was incubated with biotin or biotin‐labeled TDP‐43, followed by pull‐down with streptavidin beads (Figure 1G). Our pull‐down experiments visualized by the anti‐PDI antibody and anti‐TDP‐43 antibody verified that TDP‐43 interacted with both wild‐type PDI and dnPDI, but did not bind to PDI Δ*b*′ mutant (Figure 1G), indicating that the *b*′ domain of PDI is crucial for the interaction between TDP‐43 and PDI in vitro. Our co‐IP experiments, visualized by the anti‐TDP‐43 antibody and anti‐HA antibody, verified that endogenous TDP‐43 interacted with both wild‐type PDI and dnPDI, but did not bind to PDI Δ*b*′ mutant, in N2a cells transiently transfected with HA‐tagged PDI (wild‐type PDI, dnPDI, or PDI Δ*b*′ mutant) treated with 1 mm H_2_O_2_ for 1 h (Figure 1H). Therefore, cytoplasmic endogenous TDP‐43 directly interacts with PDI through the *b*′ domain of the enzyme in neuronal cells.

### 2.2 Wild‐Type PDI Slows Down Phase Separation of TDP‐43 Through Direct Interaction With TDP‐43

**FIGURE 2:**
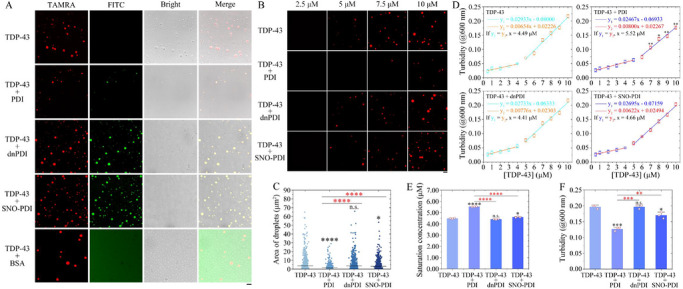
Wild‐type PDI slows down phase separation of TDP‐43 through direct interaction with TDP‐43. (A) TDP‐43 selectively recruits PDI into its phase‐separated condensate. Fluorescence images of in vitro phase‐separated droplets (red; Merge: yellow) of 10 µm TAMRA‐labeled bacterial‐purified TDP‐43 incubated with Tris‐HCl buffer (pH 8) containing 10 µm FITC‐labeled bacterial‐purified wild‐type PDI, dnPDI, or SNO‐PDI (green) and 10% (w/v) PEG 3350 on ice, and FITC‐labeled BSA as a control. (B) Concentration‐dependent TDP‐43 condensate formation regulated by PDI. Fluorescence images of 2.5, 5, 7.5, or 10 µm recombinant TDP‐43 labeled by TAMRA (red) and incubated with Tris‐HCl buffer containing wild‐type PDI, dnPDI, or SNO‐PDI at 10 µm and 10% PEG 3350 on ice. Scale bars, 10 µm. (C) Scatter plot analyzing the area of droplets (TDP‐43 condensates, µm^2^) coacervated with wild‐type PDI (pale blue), dnPDI (blue), SNO‐PDI (dark blue), or without PDI (control, pale gray‐blue), and showing the quantification of data from *n *= 1000 condensates examined over 3 independent experiments. Black lines represent the mean of area of droplets. 10 µm TDP‐43 + 10 µm wild‐type PDI, *p* = 1.43 × 10^−15^; 10 µm TDP‐43 + 10 µm dnPDI, *p* = 0.921 or 1.99 × 10^−19^ (red); and 10 µm TDP‐43 + 10 µm SNO‐PDI, *p* = 0.0424 or 9.78 × 10^−16^ (red). (D) The dependence of turbidity changes for phase separation of TDP‐43, TDP‐43 + 10 µm wild‐type PDI, TDP‐43 + 10 µm dnPDI, or TDP‐43 + 10 µm SNO‐PDI on the concentration of TDP‐43 ([TDP‐43]) is expressed as the mean ± SD (with error bars) of values obtained in three independent experiments. The turbidity of TDP‐43 condensates was measured at 600 nm at 25°C. Representative calculation based on turbidity measurements to determine saturation concentrations of TDP‐43 or TDP‐43 + dnPDI (open circle) and TDP‐43 + wild‐type PDI or TDP‐43 + SNO‐PDI (open square). The orange and the red lines are drawn through data points indicating the absence of phase separation, whereas the cyan and the blue lines are drawn through data points in which robust phase separation of TDP‐43 occurs. The concentration of protein at which these two lines intersect is an estimation of the saturation concentration. (E,F) Saturation concentration of TDP‐43 (E) and turbidity of TDP‐43 condensates (F) (open red circles shown in scatter plots) are expressed as the mean ± SD (with error bars) of values obtained in three biologically independent experiments. TDP‐43 + wild‐type PDI, *p* = 0.000002 (E) and 0.00012 (F); TDP‐43 + dnPDI, *p* = 0.0545 or 0.00000068 (E) and 1 or 0.00012 (F); and TDP‐43 + SNO‐PDI, *p* = 0.0299 or 0.0000000092 (E) and 0.0161 or 0.0029 (F). Statistical analyses were performed using two‐sided Student's *t*‐tests. Values of *p* < 0.05 indicate statistically significant differences. The following notation is used throughout: *p < 0.05, **p < 0.01, ***p < 0.001, and ****p < 0.0001 relative to control (the saturation concentration for TDP‐43 or TDP‐43 + wild‐type PDI). n.s., not significant.

Given that wild‐type PDI specifically interacts with TDP‐43 in the cytoplasm of neuronal cells under pathological stress conditions (Figure 1), we predicted that wild‐type PDI might regulate the phase separation of TDP‐43 via interaction with the protein. We used confocal microscopy and turbidity experiments as well as fluorescence recovery after photobleaching (FRAP) [35, 36, 37, 38, 39, 40, 41, 42, 44, 45, 46] to test this hypothesis. The freshly bacterial‐purified full‐length human TDP‐43 (residues 1−414), labeled with 5(6)‐carboxy‐tetramethylrhodamine *N*‐succinimidyl ester (TAMRA, red fluorescence) and incubated with Tris‐HCl buffer containing 10% PEG 3350 on ice, underwent phase separation in vitro and formed protein condensates (Figure 2A,B; Figure S5A–C). During the first 20 min after droplet initiation, two small liquid condensates of TDP‐43 rapidly fused into one larger liquid droplet within 2 s (Figure S5A,B). After 60 min of incubation, however, two small liquid condensates of TDP‐43 slowly fused into one larger gel‐like/solid‐like droplet within 60 s, and the fusion rate of the former was much greater than that of the latter (Figure S5C), suggesting that liquid‐to‐solid phase transitions of TDP‐43 occur under these conditions. TDP‐43 formed abundant liquid droplets within 20 min after droplet initiation, and protein condensates formed by TDP‐43 (10 µm) became much smaller in the presence of 10 µm wild‐type human PDI than in the absence of PDI (Figure 2A,B). We obtained fluorescence images of in vitro phase‐separated TDP‐43 droplets with recombinant, wild‐type human PDI, dnPDI, and S‐nitrosylated PDI (SNO‐PDI) (Figure 2A). Here, SNO‐PDI, observed in the brain of patients with ALS and AD [21, 22], was assessed by the release of NO, which causes the conversion of DAN into the fluorescent compound NAT (Figure S6A). TDP‐43 (10 µm) labeled with TAMRA (red fluorescence) and incubated with Tris‐HCl buffer containing 10 µm FITC‐labeled wild‐type PDI, dnPDI, or SNO‐PDI (green fluorescence) and 10% PEG 3350 on ice and subjected to phase separation in vitro (Figure 2A). TDP‐43‐demixed droplets (red; Merge: yellow) coacervated with wild‐type PDI, dnPDI, or SNO‐PDI (green) were observed by confocal microscopy with excitation at 552 and 488 nm, respectively (Figure 2A). Notably, TDP‐43 selectively recruited wild‐type PDI and dnPDI (or SNO‐PDI) but did not recruit nonspecific bovine serum albumin (BSA), a negative control, to its phase‐separated condensate (Figure 2A). Our control experiments demonstrated that wild‐type PDI, dnPDI, SNO‐PDI, or BSA alone did not form liquid droplets (Figure S6B–E). We then investigated concentration‐dependent TDP‐43 condensate formation regulated by PDI. In total, 2.5 µm TDP‐43 did not form any liquid droplets, TDP‐43 at 5 µm did produce a few liquid droplets, and 7.5 or 10 µm TDP‐43 formed abundant liquid droplets in Tris‐HCl buffer containing 10% PEG 3350 on ice (Figure 2B). 10 µm wild‐type PDI strongly slowed the in vitro phase separation of TDP‐43 (5, 7.5, and 10 µm) (Figure 2B). In sharp contrast, 10 µm dnPDI and 10 µm SNO‐PDI, two negative controls, and abnormal forms of PDI, did not slow the in vitro phase separation of TDP‐43 (10 µm) (Figure 2A,B). Quantification of droplet area revealed that wild‐type PDI significantly decreased the area of TDP‐43 condensates (*p* = 1.43 × 10^−15^), but dnPDI did not significantly reduce, and SNO‐PDI only mildly reduced the area of TDP‐43 condensates (*p* = 0.921 and 0.0424, respectively) (Figure 2C). Therefore, TDP‐43 selectively recruits wild‐type PDI to its phase‐separated condensate, which in turn slows the in vitro phase separation of TDP‐43. Together, these data demonstrate that wild‐type PDI recruited by TDP‐43 forms a complex with TDP‐43 to inhibit the in vitro phase separation of TDP‐43.

A generally accepted measure of protein propensity for phase separation is the saturation concentration [33, 35, 36, 38, 41, 46, 57, 58, 59, 60]. To assess the effects of PDI on the propensity of TDP‐43 for phase separation, we determined the saturation concentrations of TDP‐43 in the absence and presence of PDI by measuring the turbidity of TDP‐43 condensates at 600 nm as a function of the concentration of TDP‐43 (Figure 2D). The turbidity of a solution does not increase when no phase separation occurs, based on the absorbance at 600 nm (Figure 2D). We then compared the saturation concentrations of TDP‐43 and the turbidity of TDP‐43 condensates in the absence and presence of PDI (Figure 2E,F). Importantly, we found that the saturation concentration of TDP‐43 in the presence of wild‐type PDI (5.54 ± 0.02 µm, *p* = 0.000002) was significantly greater than that of TDP‐43 in the absence of PDI (4.49 ± 0.04 µm), but dnPDI and SNO‐PDI did not significantly change the saturation concentration of TDP‐43 (4.41 ± 0.03 µm, *p* = 0.0545; 4.60 ± 0.05 µm, *p* = 0.0299) (Figure 2E). We also found that the turbidity of TDP‐43 condensates in the presence of wild‐type PDI (0.127 ± 0.006, *p* = 0.00012) was significantly lower than that of TDP‐43 condensates in the absence of PDI (0.197 ± 0.006), but dnPDI and SNO‐PDI did not significantly change the turbidity of TDP‐43 condensates (0.197 ± 0.006, *p* = 1; 0.17 ± 0.01, *p* = 0.016) (Figure 2F). Together, these data demonstrate that wild‐type PDI strongly slows down phase separation of TDP‐43 through direct interaction with TDP‐43, shifting the equilibrium phase boundary to higher protein concentrations.

We then investigated and evaluated the dynamics of in vitro phase separation of droplets of TDP‐43 with/without PDI by FRAP (Figure S7A–E). FRAP of phase‐separated TDP‐43 droplets without PDI or with two negative controls, dnPDI and SNO‐PDI, revealed fluorescence recoveries of 32.6% ± 0.7%, 31.6% ± 3.6%, and 37.5% ± 4.6%, respectively, within 250 s (Figure S7E,F). In sharp contrast, FRAP of phase‐separated TDP‐43 droplets coacervated with wild‐type PDI revealed a much greater fluorescence recovery (46.7% ± 2.2)% within 250 s (Figure S7E,F). According to Figure S7, wild‐type PDI significantly enhanced fluorescence recovery. This means that wild‐type PDI increases the fluidity of TDP‐43 condensates, possibly through blocking liquid‐to‐solid transitions in phase‐separated TDP‐43 condensates. The aforementioned experiments revealed that wild‐type PDI enhances fluorescence recovery and negatively modulates phase separation of TDP‐43.

Together, these data strongly suggest that the interactions between TDP‐43 and wild‐type PDI control liquidity and that wild‐type PDI enhances TDP‐43 mobility via specific interactions with TDP‐43. Importantly, wild‐type PDI slows phase separation of TDP‐43 by increasing the saturation concentration for TDP‐43 condensation. Therefore, wild‐type PDI is a key factor in modulating TDP‐43 liquid‐phase condensation under pathological stress conditions.

### 2.3 TDP‐43 Accumulates and Exhibits Overlapping Distribution With G3BP1 in the Cytoplasm of the Brain of ALS Patients

**FIGURE 3:**
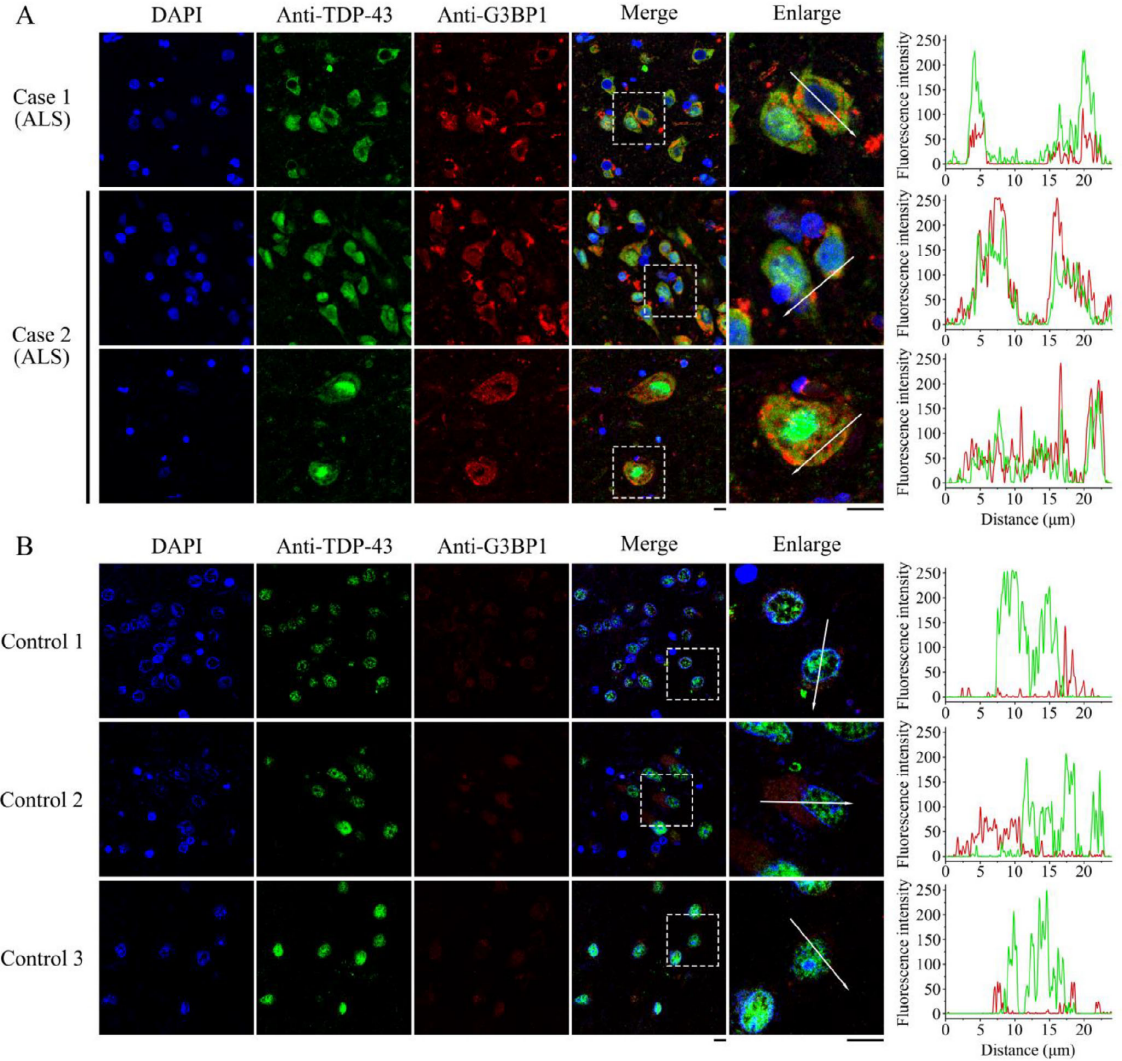
Mislocalization of TDP‐43 to the cytoplasm and the overlapping distribution of cytoplasmic TDP‐43 condensates and G3BP1 were observed in the brain of patients with ALS. (A) Double immunofluorescence staining (anti‐TDP‐43 and anti‐G3BP1) of paraffin brain sections from two patients with ALS (Cases 1 and 2). The sampling for ALS patient one and ALS patient two was the same as that in Figure S2. The nuclei were stained with DAPI (blue); signals were detected with antibodies against TDP‐43 (green) and G3BP1 (red). The enlarged regions (right) show ninefold enlarged images from the merged images. Orange puncta indicate overlapping distribution of cytoplasmic TDP‐43 condensates (green) and G3BP1 (red) in two ALS patients. (B) Double immunofluorescence staining (anti‐TDP‐43 and anti‐G3BP1) of paraffin brain sections from Controls 1 to 3. The healthy controls one to three were sampled in the hippocampus and temporal lobe. The experimental conditions are the same as those in (A). Orange puncta and cytoplasmic TDP‐43 (green) were not observed, indicating that TDP‐43 did not colocalize with G3BP1 (red) in the brains of these three controls. The control samples do have normal levels of G3BP1 (red). The scale bars represent 10 µm. Line‐scan analyses (right) show the spatial overlap between cytoplasmic TDP‐43 (green) and G3BP1 (red) signals of the two ALS patients (A) and the three controls (B). The fluorescence of each group was separately normalized using the average of the maximum green or red fluorescence values in the selected regions.

G3BP1 is an RNA‐binding protein that is widely recognized as a core component of stress granules; G3BP1 triggers phase separation to assemble stress granules and undergoes K63‐linked ubiquitination to modulate stress granule disassembly [58, 59, 61, 62, 63, 64]. Given that G3BP1 is essential for stress granule assembly and facilitates biomolecular condensate formation [59, 61, 62, 63, 64, 65], we predicted that G3BP1 might colocalize with cytoplasmic TDP‐43 in the brain of ALS patients. To test this hypothesis, we obtained confocal images of paraffin brain sections from two patients with ALS and three healthy controls (Figure 3 and Table 1). The brain sections were doubly immunostained with the anti‐TDP‐43 antibody (green) and anti‐G3BP1 antibody (red), stained with DAPI (blue), and visualized by confocal microscopy. Notably, not only abundant G3BP1‐positive stress granules (red dots) but also the overlapping distribution of cytoplasmic TDP‐43 (green) and G3BP1 (red) were observed in the brain of the two ALS patients (Figure 3A, orange dots in the merged images). Mislocalization of TDP‐43 to the cytoplasm and the overlapping distribution of cytoplasmic TDP‐43 condensates and G3BP1 were observed in the brain of patients with ALS (Figure 3A). In sharp contrast, orange puncta and cytoplasmic TDP‐43 were not observed, indicating that TDP‐43 did not colocalize with G3BP1 in the brains of the three age‐matched healthy controls (Figure 3B). Our findings showed that the interaction between cytoplasmic TDP‐43 condensates and cytoplasmic G3BP1 in the ALS brain drastically increased compared with that in the healthy control brain (Figure 3).

### 2.4 Wild‐Type PDI Blocks the Mislocalization of TDP‐43 to the Cytoplasm Induced by H_2_O_2_

Increased levels of oxidative stress and ER stress are both observed in the brain of patients with ALS [66, 67]. To mimic oxidative stress and ER stress conditions in neuronal cells, we treated N2a neuroblastoma cells with 1 mm H_2_O_2_ for 1 h (persistent oxidative stress), and observed a clear shift of endogenous TDP‐43 to the cytoplasm of the cell line (Figure S8A,B). We then compared the amount of endogenous TDP‐43 in the cytoplasm and nucleus of N2a cells in the absence and presence of transiently expressed HA‐tagged PDI (Figure S8B). Importantly, we found that under persistent oxidative stress, the amount of endogenous TDP‐43 in cytoplasm of the cell line in the presence of HA‐tagged wild‐type PDI (0.481 ± 0.045) was significantly lower than that in the absence of PDI (0.937 ± 0.045), and the amount of endogenous TDP‐43 in nucleus of the cell line in the presence of HA‐tagged wild‐type PDI (0.639 ± 0.045) was significantly higher than that in the absence of PDI (0.396 ± 0.026) (Figure S8B). However, under persistent oxidative stress, dnPDI and PDI Δ*b′* mutant, two negative controls, and abnormal forms of PDI, did not significantly change the amount of endogenous TDP‐43 in the cytoplasm of the cell line (0.845 ± 0.070 and 1.002 ± 0.082) and the amount of endogenous TDP‐43 in the nucleus (0.434 ± 0.005 and 0.383 ± 0.027) (Figure S8B). We next took confocal images of N2a cells transiently expressing EGFP‐TDP‐43 and empty vector pcDNA3.1 treated with 1 mm H_2_O_2_ for 20 min (short oxidative stress) and 1 h, respectively, transiently expressing EGFP‐TDP‐43 and HA‐tagged wild‐type PDI treated with 1 mm H_2_O_2_ for 20 min and 1 h, respectively, and transiently expressing EGFP‐TDP‐43 and HA‐tagged dnPDI also treated with 1 mm H_2_O_2_ for 20 min and 1 h, respectively. N2a cells transiently expressing EGFP‐TDP‐43 and pcDNA3.1 were used as a control. The above cells were immunostained with the anti‐HA antibody (red), stained with DAPI (blue), and observed by confocal microscopy (Figure S8C,D). Under the pathological stress condition, EGFP‐TDP‐43 (green) was partly mislocalized from the nucleus to the cytoplasm of the cell line (Figure S8C,D). Importantly, we found that under persistent oxidative stress, wild‐type PDI blocked the mislocalization of TDP‐43 to the cytoplasm of the cell line, but dnPDI and PDI Δ*b′* mutant, two negative controls, and abnormal forms of PDI, did not block the mislocalization of TDP‐43 to the cytoplasm (Figure S8B,D). We unexpectedly found that in the 20‐min images, mislocalization of TDP‐43 was still observed despite PDI overexpression (Figure S8C), which well demonstrates that the PDI‐TDP‐43 interaction is critical for preventing the mislocalization of TDP‐43 to the cytoplasm. Under oxidative stress, TDP‐43 transiently comes out to the cytosol, then interacts with PDI, which later sends TDP‐43 back to the nucleus. We then compared the number of cells with nuclear‐localized TDP‐43 in N2a cells transiently expressing TDP‐43 in the absence and presence of transiently expressed PDI under stress (Figure S8E). Importantly, a significantly higher number of cells with nuclear‐localized TDP‐43 was observed in N2a cells transiently expressing both EGFP‐TDP‐43 and HA‐tagged wild‐type PDI treated with 1 mm H_2_O_2_ for 1 h than in N2a cells transiently expressing EGFP‐TDP‐43 also treated with 1 mm H_2_O_2_ for 1 h (84.14 ± 6.55 for wild‐type PDI + H_2_O_2_ vs. 27.24 ± 6.64 for control + H_2_O_2_) (Figure S8E). However, wild‐type PDI did not significantly change the number of cells with nuclear‐localized TDP‐43 in the same cells treated with 1 mm H_2_O_2_ for 20 min (67.45 ± 3.43 for wild‐type PDI + H_2_O_2_ vs. 64.77 ± 5.04 for control + H_2_O_2_) (Figure S8E). Therefore, wild‐type PDI, but not abnormal forms of PDI, significantly reduces cytoplasmic TDP‐43 accumulation in neuronal cells induced by the persistent oxidative stress, restoring TDP‐43 to the nucleus, possibly via specific interactions with TDP‐43 through the *b′* domain of the enzyme. It should be mentioned that dnPDI is a dominant‐negative mutant, and we have shown that dnPDI interacts with TDP‐43 but is defective in preventing mislocalization. These findings suggest that binding alone is not sufficient to block the mislocalization of TDP‐43 to the cytoplasm.

### 2.5 Wild‐Type PDI Disassembles TDP‐43/G3BP1 Condensates by Decreasing the Interaction of Cytoplasmic TDP‐43 With G3BP1 Induced by H_2_O_2_

**FIGURE 4:**
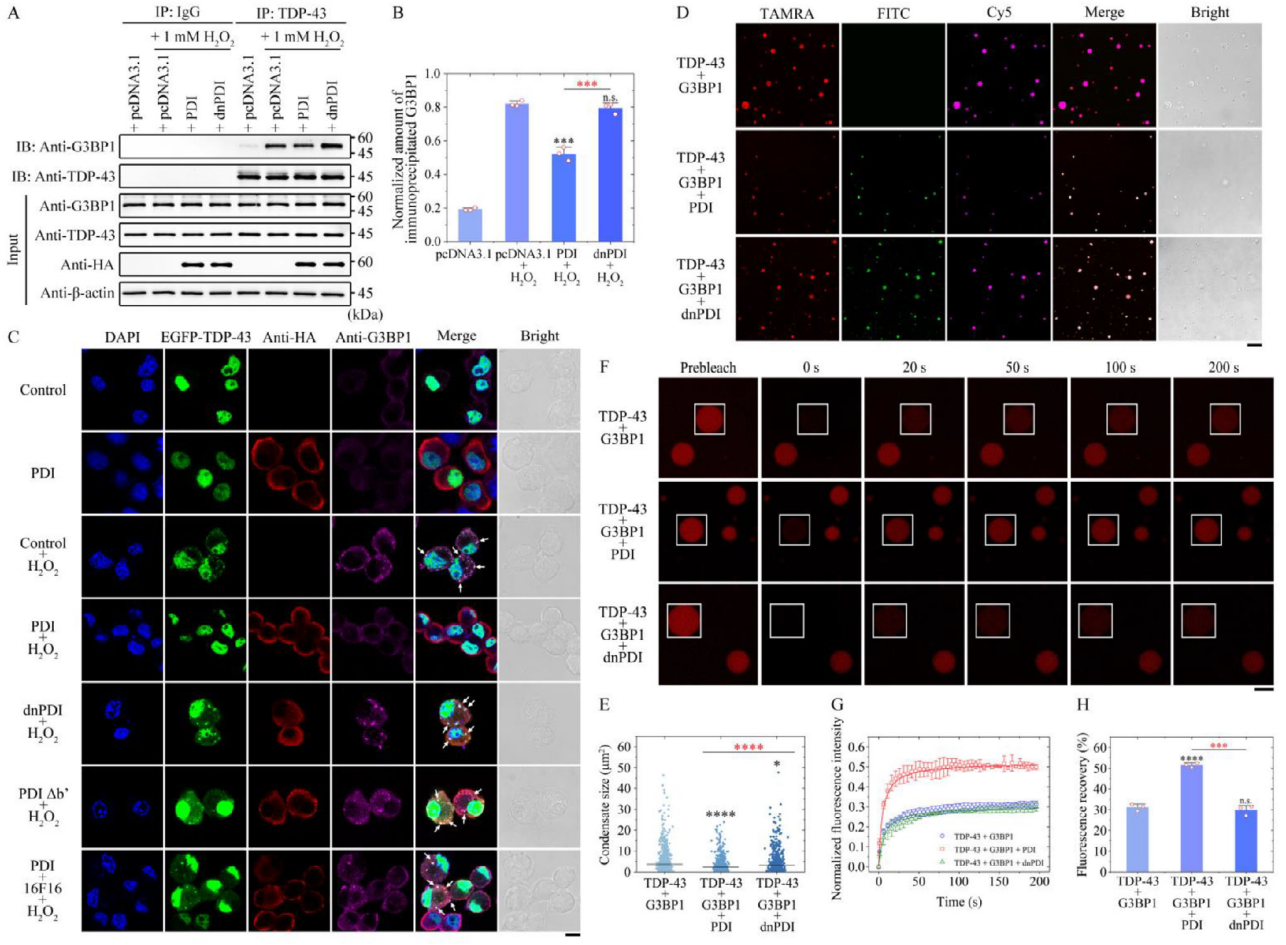
Wild‐type PDI disassembles TDP‐43/G3BP1 condensates by decreasing the interaction of cytoplasmic TDP‐43 with G3BP1 induced by H_2_O_2_. (A) Co‐IP assay to verify the interaction of endogenous TDP‐43 with endogenous G3BP1 in N2a cells transiently expressing the empty vector pcDNA3.1 treated with PBS for 1 h, transiently expressing the empty vector pcDNA3.1 treated with 1 mm H_2_O_2_ for 1 h, and transiently expressing HA‐tagged wild‐type PDI or HA‐tagged dnPDI treated with 1 mm H_2_O_2_ for 1 h. Anti‐TDP‐43 antibody‐binding beads were used for co‐IP experiments and then detected by western blotting using anti‐G3BP1, anti‐TDP‐43, anti‐HA, and anti‐β‐actin antibodies. We have added IP bands of TDP‐43 in the second row. (B) The normalized amount of immunoprecipitated G3BP1 in the aforementioned cells (open red circles shown in scatter plots) is expressed as the mean ± SD (with error bars) of values obtained in three independent experiments. Wild‐type PDI + H_2_O_2_, *p* = 0.00031; and dnPDI + H_2_O_2_, *p* = 0.270 or 0.00073 (red). (C) Wild‐type PDI disassembles TDP‐43/G3BP1 condensates in N2a cells through the *b′* domain of the enzyme. Immunofluorescence images of N2a cells transiently expressing both EGFP‐TDP‐43 and pcDNA3.1 treated with PBS for 1 h (control), transiently expressing both EGFP‐TDP‐43 and HA‐tagged wild‐type PDI treated with PBS for 1 h (PDI), transiently expressing both EGFP‐TDP‐43 and pcDNA3.1 treated with 1 mm H_2_O_2_ for 1 h (control + H_2_O_2_), transiently expressing both EGFP‐TDP‐43 and HA‐tagged wild‐type PDI treated with 1 mm H_2_O_2_ for 1 h (PDI + H_2_O_2_), transiently expressing both EGFP‐TDP‐43 and HA‐tagged dnPDI treated with 1 mm H_2_O_2_ for 1 h (dnPDI + H_2_O_2_), transiently expressing both EGFP‐TDP‐43 and HA‐tagged PDI Δ*b*’ mutant treated with 1 mm H_2_O_2_ for 1 h (PDI Δ*b*’ + H_2_O_2_), and transiently expressing both EGFP‐TDP‐43 and HA‐tagged wild‐type PDI treated with 5 µm PDI inhibitor 16F16 for 16 h and then 1 mm H_2_O_2_ for 1 h (PDI + 16F16 + H_2_O_2_), using anti‐HA (red) and anti‐G3BP1 (magenta) antibodies and staining with DAPI (blue). EGFP‐TDP‐43 (green) was detected. The white arrows indicate colocalization of TDP‐43 condensates and endogenous G3BP1 in N2a cells. (D) Wild‐type PDI disassembles TDP‐43/G3BP1 condensates in vitro. Fluorescence images of in vitro reconstituted co‐condensates (merge: deep magenta or white) of 10 µm TAMRA‐labeled bacterial‐purified TDP‐43 (red) and 5 µm CY5‐labeled bacterial‐purified G3BP1 (magenta) incubated with Tris‐HCl buffer (pH 8) containing 10 µm FITC‐labeled bacterial‐purified wild‐type PDI or dnPDI (green) and 10% (w/v) PEG 3350 on ice. Scale bars: 10 µm (C,D). (E) Scatter plot analyzing the area of droplets (co‐condensates of TDP‐43 + G3BP1, µm^2^), coacervated with wild‐type PDI (pale blue), dnPDI (blue), or without PDI (control, pale gray–blue), and showing the quantification of data from *n* = 1000 condensates examined over 3 independent experiments. Black lines represent the mean of area of droplets. 10 µm TDP‐43 + 5 µm G3BP1 + 10 µm wild‐type PDI, *p* = 6.37 × 10^−13^; and 10 µm TDP‐43 + 5 µm G3BP1 + 10 µm dnPDI, *p* = 0.0441 or 1.38 × 10^−7^ (red). (F–H) PDI enhances fluorescence recovery and negatively modulates co‐phase separation of TDP‐43 and G3BP1 in vitro. (F) FRAP analysis of selected co‐condensates of 10 µm TAMRA‐labeled bacterial‐purified TDP‐43 (red) and 5 µm bacterial‐purified G3BP1 incubated with Tris‐HCl buffer (pH 8) containing 10% PEG 3350 (TDP‐43 + G3BP1), 10 µm wild‐type PDI and 10% PEG 3350 (TDP‐43 + G3BP1 + PDI), or 10 µm dnPDI and 10% PEG 3350 (TDP‐43 + G3BP1 + dnPDI) and 10% (w/v) PEG 3350 on ice before (prebleach), during (0 s), and after photobleaching (20, 50, 100, and 200 s, respectively). The internal photobleaching is marked by a white square. Scale bar: 5 µm. (G) Normalized kinetics of fluorescence recovery data of co‐condensates of TDP‐43 + G3BP1 (blue circles), TDP‐43 + G3BP1 + wild‐type PDI (red squares), and TDP‐43 + G3BP1 + dnPDI (olive triangles) obtained from FRAP intensity, respectively. The normalized fluorescence intensity is expressed as the mean ± SD (with error bars) of values obtained in three independent experiments. The solid lines show the best single exponential fit for the fluorescence intensity‐time curves. (H) The fluorescence recovery percentage of TDP‐43/G3BP1 condensates (open red circles shown in scatter plots) is expressed as the mean ± SD (with error bars) of values obtained in three biologically independent experiments. TDP‐43 + G3BP1 + wild‐type PDI, *p* = 0.000053; and TDP‐43 + G3BP1 + dnPDI, *p* = 0.466 or 0.00013 (red). Statistical analyses were performed using two‐sided Student's *t‐*tests. Values of *p* < 0.05 indicate statistically significant differences. The following notation was used throughout: *p < 0.05, **p < 0.01, ***p < 0.001, and ****p < 0.0001 relative to the control (pcDNA3.1 + H_2_O_2_ or wild‐type PDI + H_2_O_2_) or the control (fluorescence recovery for co‐condensates of TDP‐43 and G3BP1 or co‐condensates of TDP‐43 and G3BP1 incubated with wild‐type PDI). n.s., not significant.

Given that TDP‐43 selectively recruits wild‐type PDI to its phase‐separated condensate, which in turn slows down the in vitro phase separation of TDP‐43 (Figure 2; Figure S7), we predicted that wild‐type PDI might regulate phase separation of TDP‐43 in neuronal cells through direct interaction with TDP‐43, compete with the core stress granule component G3BP1, and thus disassemble TDP‐43/G3BP1 condensates. We used co‐IP and confocal microscopy to test this hypothesis. Our co‐IP experiments with the anti‐G3BP1 antibody, anti‐TDP‐43 antibody, and anti‐HA antibody verified the interaction of endogenous TDP‐43 with endogenous G3BP1 in N2a cells transiently expressing the empty vector pcDNA3.1 and treated with 1 mm H_2_O_2_ for 1 h, and transiently expressing HA‐tagged wild‐type PDI or dnPDI and treated with 1 mm H_2_O_2_ for 1 h (Figure 4A). We then compared the amount of immunoprecipitated G3BP1 in N2a cells in the absence or presence of transiently expressed HA‐tagged PDI (Figure 4B). Notably, under H_2_O_2_‐induced oxidative stress conditions, the amount of immunoprecipitated G3BP1 in N2a cells in the presence of wild‐type PDI (0.523 ± 0.041, *p* = 0.00031) was significantly lower than that in the absence of PDI (0.820 ± 0.017), but dnPDI did not significantly change the amount of immunoprecipitated G3BP1 in N2a cells (0.795 ± 0.030, *p* = 0.270) (Figure 4B). We next obtained confocal images of N2a and HEK‐293T cells transiently expressing EGFP‐TDP‐43 and the empty vector pcDNA3.1 treated with 1 mm H_2_O_2_ for 1 h, and of N2a and HEK‐293T cells transiently expressing EGFP‐TDP‐43 and HA‐tagged wild‐type PDI or dnPDI or PDI Δ*b′* mutant treated with 1 mm H_2_O_2_ for 1 h. N2a and HEK‐293T cells transiently expressing both EGFP‐TDP‐43 and pcDNA3.1 or HA‐tagged wild‐type PDI were treated with PBS for 1 h and used as controls. The above cell lines were doubly immunostained with the anti‐HA antibody (red) and anti‐G3BP1 antibody (magenta), stained with DAPI (blue), and observed by confocal microscopy (Figure 4C; Figure S9A). Under pathological stress conditions, EGFP‐TDP‐43 (green) was partly mislocalized from the nucleus to the cytoplasm of N2a and HEK‐293T cells, and the abundant white puncta observed in confocal images represented the colocalization of cytoplasmic TDP‐43 and endogenous G3BP1 in stress granules (Figure 4C; Figure S9A). Importantly, we found that under the pathological stress condition, overexpressed wild‐type PDI blocked the mislocalization of TDP‐43 to the cytoplasm (Figure 4C; Figure S9A) and disassembled TDP‐43/G3BP1 condensates in N2a and HEK‐293T cells by significantly decreasing the interaction of cytoplasmic TDP‐43 with G3BP1 induced by H_2_O_2_ (Figure 4A–C; Figure S9A). In sharp contrast, overexpressed dnPDI or PDI Δ*b′* mutant did not block the mislocalization of TDP‐43 to the cytoplasm of N2a and HEK‐293T cells, and the abundant white puncta observed in confocal images represented the colocalization of cytoplasmic TDP‐43 and endogenous G3BP1 in stress granules in the presence of dnPDI or PD*I Δb′* mutant (Figure 4C; Figure S9A). Thus, wild‐type PDI disassembles TDP‐43/G3BP1 condensates in N2a and HEK‐293T cells through the *b′* domain of the enzyme. It should be mentioned that the control results with PDI overexpression but without stress in Figure 4C and Figure S9A represent only a representative result, which shows that without 1 mM stimulation, TDP‐43 and PDI or TDP‐43 and G3BP1 display almost no interaction. In cells overexpressing PDI and treated with PBS, EGFP‐TDP‐43 fusion protein maintained its normal nuclear localization and G3BP1 maintained its normal cytoplasmic localization, indicating that PDI cannot affect the subcellular localization of the two proteins under such conditions (Figure 4C; Figure S9A).

To test whether the basal protein level of PDI could modulate these effects, we next performed experiments using inhibition or knockdown of the endogenous PDI to complement the overexpression experiments, and obtained confocal images of N2a cells transiently expressing both EGFP‐TDP‐43 and HA‐tagged wild‐type PDI treated with 5 µm PDI inhibitor 16F16 for 16 h and then 1 mm H_2_O_2_ for 1 h (Figure 4C). Under pathological stress conditions combined with the inhibition of PDI activity, more EGFP‐TDP‐43 (green) protein was mislocalized from the nucleus to the cytoplasm of N2a cells, and the abundant white puncta observed in confocal images represented the colocalization of cytoplasmic TDP‐43 and endogenous G3BP1 in stress granules, further supporting that wild‐type PDI blocks the mislocalization of TDP‐43 to the cytoplasm and disassembles TDP‐43/G3BP1 condensates in N2a cells (Figure 4C). Importantly, the inhibition of PDI activity markedly reduced not only its protective effect on maintaining nuclear localization of TDP‐43 but also its ability to disassemble TDP‐43/G3BP1 condensates in N2a cells (Figure 4C), thereby providing evidence that what we observed could be happening in endogenous conditions. The aforementioned experiments with 16F16 do not fully address the contribution of basal PDI (Figure 4C). Because the direct interaction between PDI and TDP‐43 is primarily demonstrated in overexpression systems (e.g., Figure 1A), endogenous validation could provide stronger support. Our co‐IP experiments with the anti‐G3BP1 antibody, anti‐TDP‐43 antibody, and anti‐PDI antibody verified the interaction of endogenous TDP‐43 with endogenous G3BP1 in HEK‐293T cells treated with 1 mm H_2_O_2_ for 1 h, and treated with 5 µm PDI inhibitor 16F16 for 16 h and then 1 mm H_2_O_2_ for 1 h (Figure S9B). We then compared the amount of immunoprecipitated endogenous G3BP1 in HEK‐293T cells treated with PBS or PDI inhibitor 16F16 under H_2_O_2_ stress (Figure S9C). Notably, under H_2_O_2_‐induced oxidative stress conditions, the amount of immunoprecipitated G3BP1 in HEK‐293T cells in the presence of 16F16 (0.909 ± 0.054, *p* = 0.00038) was significantly higher than that in the absence of 16F16 (0.524 ± 0.028) (Figure S9C). We show that endogenous PDI, through its specific interaction with endogenous TDP‐43, competitively displaces endogenous G3BP1 to disassemble TDP‐43/G3BP1 condensates (Figure S9B,C). We next provided a clearer comparison between H_2_O_2_‐treated TDP‐43‐transfected cells and H_2_O_2_ + 16F16‐treated TDP‐43‐transfected cells (without HA‐tagged wild‐type PDI) and obtained confocal images of HEK‐293T cells transiently expressing EGFP‐TDP‐43 treated with 1 mm H_2_O_2_ for 1 h, and of HEK‐293T cells transiently expressing EGFP‐TDP‐43 treated with 5 µm 16F16 for 16 h and then 1 mm H_2_O_2_ for 1 h. HEK‐293T cells transiently expressing EGFP‐TDP‐43 were treated with PBS for 1 h and used as controls. The above cell lines were doubly immunostained with the anti‐PDI antibody (red) and anti‐G3BP1 antibody (magenta), stained with DAPI (blue), and observed by confocal microscopy (Figure S9D). More TDP‐43/G3BP1 condensates were observed in the 16F16‐treated condition, indicating that basal PDI contributes (Figure S9D). Under pathological stress conditions, the inhibition of endogenous PDI activity significantly increased oxidative stress‐induced interaction between endogenous TDP‐43 and endogenous G3BP1 (Figure S9B,C), thereby promoting TDP‐43/G3BP1 condensate formation (Figure S9D). Therefore, basal PDI disassembles TDP‐43/G3BP1 condensates in HEK‐293T cells by significantly decreasing the interaction of cytoplasmic TDP‐43 with G3BP1 induced by H_2_O_2_.

We then designed and performed experiments where all three of these proteins were made in recombinant form and considered at the same time, and used confocal microscopy to examine whether wild‐type PDI also disassembles TDP‐43/G3BP1 condensates in vitro. The freshly bacterial‐purified G3BP1, labeled with CY5 (magenta fluorescence) and incubated with Tris‐HCl buffer containing 10% PEG 3350 on ice, underwent co‐phase separation with the freshly bacterial‐purified full‐length TDP‐43 labeled with TAMRA (red fluorescence) in the same buffer (Figure 4D). Abundant G3BP1 demixed droplets (magenta) fused with droplets of TDP‐43 (red) were observed by confocal microscopy, with excitation at 638 and 552 nm, forming TDP‐43/G3BP1 condensates in vitro (merge: deep magenta) (Figure 4D). We obtained fluorescence images of in vitro reconstituted TDP‐43/G3BP1 condensates incubated with recombinant wild‐type PDI and dnPDI (Figure 4D). G3BP1 (5 µm) labeled with CY5 (magenta fluorescence) and TDP‐43 (10 µm) labeled with TAMRA (red fluorescence) were incubated with Tris‐HCl buffer containing 10 µm FITC‐labeled wild‐type PDI or dnPDI (green fluorescence) and 10% PEG 3350 on ice (Figure 4D). Importantly, wild‐type PDI (green) competed with G3BP1 (magenta) and significantly decreased the number and size of TDP‐43/G3BP1 condensates (merge: white) in vitro (Figure 4D). Quantification of droplet area revealed that wild‐type PDI significantly decreased the area of TDP‐43/G3BP1 condensates (*p* = 6.37 × 10^−13^), but dnPDI only mildly reduced the area of TDP‐43/G3BP1 condensates (*p* = 0.0441) (Figure 4E). Therefore, wild‐type PDI competes with G3BP1 and disassembles TDP‐43/G3BP1 condensates in vitro through direct interaction with TDP‐43.

Next, we investigated and evaluated the dynamics of in vitro phase separation of co‐condensates of TDP‐43 and G3BP1 with/without PDI by FRAP (Figure 4F,G). FRAP of phase‐separated TDP‐43/G3BP1 condensates without PDI or with a negative control dnPDI revealed fluorescence recoveries of 31.1% ± 1.6% and 29.7% ± 2.4%, respectively, within 200 s (Figure 4G,H). In sharp contrast, FRAP of phase‐separated TDP‐43/G3BP1 condensates coacervated with wild‐type PDI revealed a much greater fluorescence recovery (51.5 ± 1.1)% within 200 s (Figure 4G,H), which is even greater than the fluorescence recovery of TDP‐43 droplets coacervated with wild‐type PDI, (46.7 ± 2.2)% within 250 s (Figure S7E,F). According to Figure 4G,H and Figure S7E,F, wild‐type PDI enhanced the fluorescence recovery of TDP‐43/G3BP1 condensates more significantly than that of TDP‐43 droplets. This means that wild‐type PDI significantly increases the fluidity of TDP‐43/G3BP1 condensates through direct interaction with TDP‐43 to block liquid‐to‐solid transitions in phase‐separated TDP‐43/G3BP1 condensates.

We have shown that dnPDI, a dominant‐negative mutant, interacts with TDP‐43 but does not disassemble condensates. These findings suggest that binding alone is not sufficient to disassemble TDP‐43/G3BP1 condensates. This points to possible alternative mechanisms, such as redox regulation or disulfide bond formation. Our additional experiments demonstrated that 10 µm wild‐type PDI strongly slowed the in vitro phase separation of TDP‐43 (10 µm) but did not slow the in vitro phase separation of TDP‐43‐iodoacetamide (IAM) (10 µm) (Figure S10A). Here, the normalized amounts of free cysteine thiols in TDP‐43‐IAM and TDP‐43 were determined by Total Thiol Assay Kit with DTNB to be 0.0735 ± 0.0674 and 1 ± 0.0674, respectively, indicating that IAM almost completely blocked free cysteine thiols of TDP‐43 (Figure S10B). Quantification of droplet area revealed that both IAM and wild‐type PDI significantly decreased the area of TDP‐43 condensates (Figure S10C). However, not only did dnPDI not significantly reduce the area of TDP‐43 condensates, but wild‐type PDI did not significantly reduce the area of TDP‐43‐IAM condensates (Figure S10C). Therefore, wild‐type PDI strongly slows the in vitro phase separation of TDP‐43 through free cysteine residues of the protein, and this regulatory effect is abolished when the free cysteine residues of TDP‐43 are blocked by IAM, given that disulfide bonds in TDP‐43 have been shown to be critical for its phase separation [48].

### 2.6 Wild‐Type PDI Antagonizes Pathological Phosphorylation of TDP‐43 and TDP‐43 Pathological Aggregates Induced by H_2_O_2_

**FIGURE 5:**
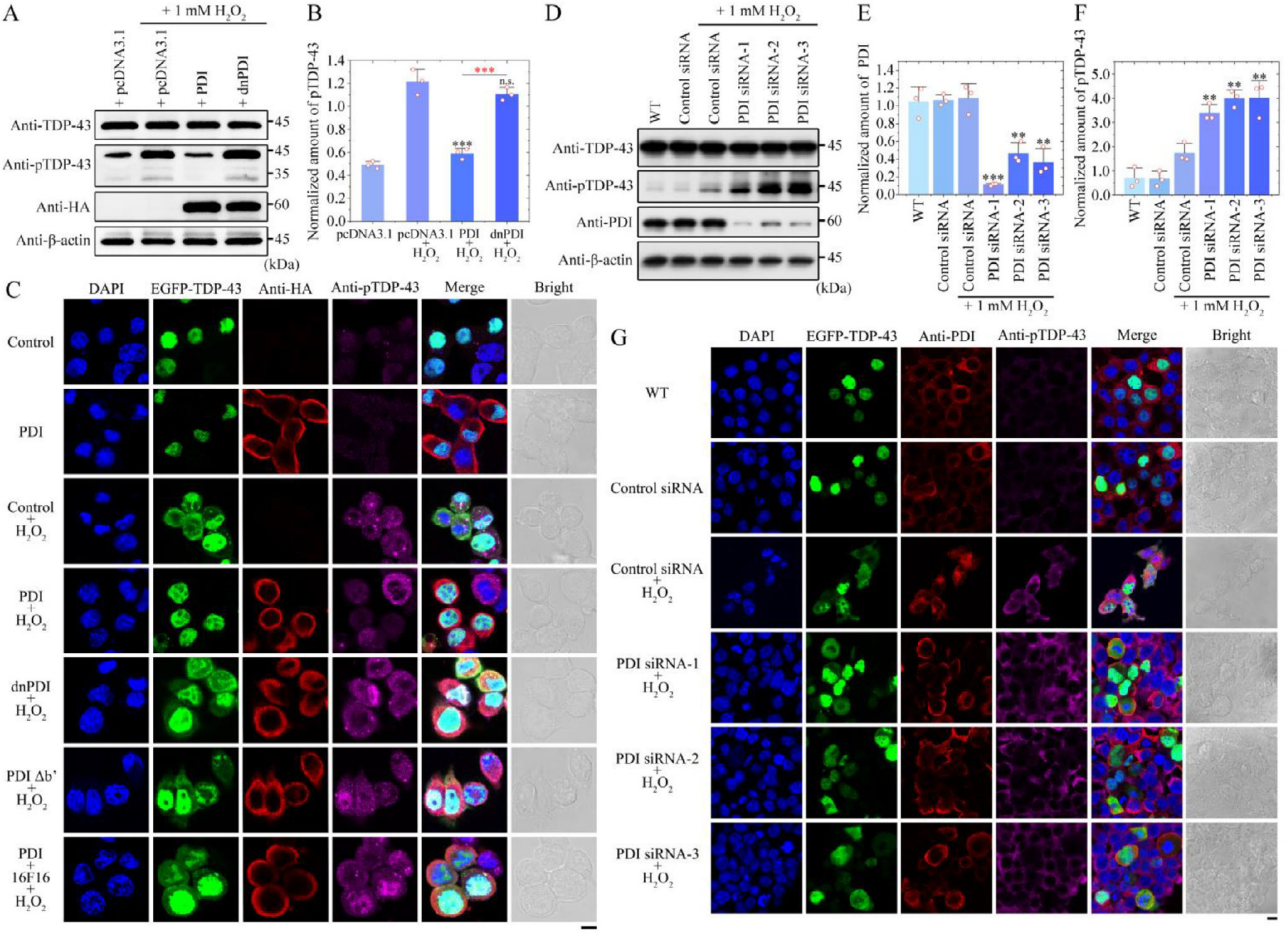
Wild‐type PDI antagonizes H_2_O_2_‐induced pathological phosphorylation of TDP‐43 through the *b′* domain of the enzyme. (A) Western blot for phosphorylated endogenous TDP‐43 (pTDP‐43) in N2a cells transiently expressing empty vector pcDNA3.1 treated with PBS for 1 h, transiently expressing pcDNA3.1 treated with 1 mm H_2_O_2_ for 1 h, and transiently expressing HA‐tagged wild‐type PDI or HA‐tagged dnPDI treated with 1 mm H_2_O_2_ for 1h. The cell lysates from the aforementioned cells were probed by anti‐TDP‐43, anti‐pTDP‐43 (pSer409/410), anti‐HA, and anti‐β‐actin antibodies. (B) The normalized amount of pTDP‐43 in the aforementioned cells (open red circles shown in scatter plots) is expressed as the mean ± SD (with error bars) of values obtained in three independent experiments. Wild‐type PDI + H_2_O_2_, *p* = 0.00083; and dnPDI + H_2_O_2_, *p* = 0.211 or 0.00026 (red). (C) Immunofluorescence imaging of N2a cells transiently expressing both EGFP‐TDP‐43 and pcDNA3.1 treated with PBS for 1 h (control), transiently expressing both EGFP‐TDP‐43 and HA‐tagged wild‐type PDI treated with PBS for 1 h (PDI), transiently expressing both EGFP‐TDP‐43 and pcDNA3.1 treated with 1 mm H_2_O_2_ for 1 h (control + H_2_O_2_), transiently expressing both EGFP‐TDP‐43 and HA‐tagged wild‐type PDI treated with 1 mm H_2_O_2_ for 1 h (PDI + H_2_O_2_), transiently expressing both EGFP‐TDP‐43 and HA‐tagged dnPDI also treated with 1 mm H_2_O_2_ for 1 h (dnPDI + H_2_O_2_), transiently expressing both EGFP‐TDP‐43 and HA‐tagged PDI Δ*b*′ mutant treated with 1 mm H_2_O_2_ for 1 h (PDI Δ*b*′ + H_2_O_2_), and transiently expressing both EGFP‐TDP‐43 and HA‐tagged wild‐type PDI treated with 5 µm PDI inhibitor 16F16 for 16 h and then 1 mm H_2_O_2_ for 1 h (PDI + 16F16 + H_2_O_2_), using antibodies against HA (red) and pTDP‐43 (magenta) and staining with DAPI (blue). EGFP‐TDP‐43 (green) was observed. (D) Western blot for phosphorylated endogenous TDP‐43 (pTDP‐43) in wild‐type (WT) HEK‐293T cells treated with PBS for 1 h, HEK‐293T cells transiently expressing negative control siRNA treated with PBS for 1 h, transiently expressing negative control siRNA treated with 1 mm H_2_O_2_ for 1 h, and transiently expressing PDI siRNA‐1, ‐2, or ‐3 treated with 1 mm H_2_O_2_ for 1 h. The cell lysates from the aforementioned cells were probed by anti‐TDP‐43, anti‐pTDP‐43 (pSer409/410), anti‐PDI, and anti‐β‐actin antibodies. (E) The normalized amount of PDI in the aforementioned cells (open red circles shown in scatter plots) is expressed as the mean ± SD (with error bars) of values obtained in three independent experiments. PDI siRNA‐1 + H_2_O_2_, *p* = 0.00051; PDI siRNA‐2 + H_2_O_2_, *p* = 0.00603; PDI siRNA‐3 + H_2_O_2_, *p *= 0.00505. (F) The normalized amount of pTDP‐43 in the aforementioned cells (open red circles shown in scatter plots) is expressed as the mean ± SD (with error bars) of values obtained in three independent experiments. PDI siRNA‐1 + H_2_O_2_, *p* = 0.00573; PDI siRNA‐2 + H_2_O_2_, *p* = 0.00184; and PDI siRNA‐3 + H_2_O_2_, *p* = 0.00876. Statistical analyses were performed using a two‐sided Student's *t*‐test. Values of *p* < 0.05 indicate statistically significant differences. The following notation is used throughout: *p < 0.05; **p < 0.01; and ***p < 0.001 relative to control (pcDNA3.1 + H_2_O_2_ or wild‐type PDI + H_2_O_2_) (B) or control (negative control siRNA + H_2_O_2_) (E,F). (G) immunofluorescence imaging of the same HEK‐293T cells as in (D), except for HEK‐293T cells transiently expressing EGFP‐TDP‐43, using antibodies against PDI (red) and pTDP‐43 (magenta) and staining with DAPI (blue). EGFP‐TDP‐43 (green) was observed. Scale bars: 10 µm (C,G).

Given that TDP‐43 selectively recruits wild‐type PDI into its phase‐separated condensate, which in turn slows down the in vitro phase separation of TDP‐43 (Figure 2; Figure S7) and disassembles TDP‐43/G3BP1 condensates by decreasing the interaction of cytoplasmic TDP‐43 with G3BP1 induced by H_2_O_2_ (Figure 4), we predicted that wild‐type PDI might further antagonize pathological phosphorylation and aggregation of TDP‐43 in neuronal cells under such oxidative stress conditions. We employed Western blotting, confocal microscopy, and immunogold electron microscopy [8, 14, 29, 39, 42] to test this hypothesis. To test the functional relevance of the observations that we made in vitro and in cells with TDP‐43 phase separation, we used the same experimental groups of N2a cells as those in Figure 4A–C and the same experimental groups of HEK‐293T cells as those in Figure S9A. We evaluated pathological phosphorylation of TDP‐43 using anti‐pSer409/410 antibody, the most common anti‐pTDP‐43 antibody [4, 17, 18, 37, 44, 45, 51]. The cell lysates from the above two cell lines were probed by the anti‐pTDP‐43 antibody, anti‐TDP‐43 antibody, anti‐HA antibody, and anti‐β‐actin antibody (Figure 5A; Figure S11A). Treatment of H_2_O_2_ strongly promoted pathological phosphorylation of endogenous TDP‐43 in the two cell lines (Figure 5A; Figure S11A). We then compared the amount of pathologically phosphorylated endogenous TDP‐43 in the two cell lines in the absence and presence of transiently expressed HA‐tagged PDI (Figure 5B; Figure S11B). Importantly, we found that under the H_2_O_2_‐induced oxidative stress condition, the amount of pathologically phosphorylated endogenous TDP‐43 in the two cell lines in the presence of wild‐type PDI (0.590 ± 0.045, *p* = 0.00083; 0.608 ± 0.045, *p* = 0.00095) was significantly lower than that in the absence of PDI (1.21 ± 0.11; 0.948 ± 0.018), but dnPDI did not significantly change the amount of pathologically phosphorylated endogenous TDP‐43 in the two cell lines (1.106 ± 0.058, *p* = 0.211; 0.947 ± 0.050, *p* = 0.977) (Figure 5B; Figure S11B). We next took confocal images of the above experimental groups of N2a and HEK‐293T cells, which were doubly immunostained with the anti‐HA antibody (red) and anti‐pTDP‐43 antibody (magenta) and stained with DAPI (blue) (Figure 5C; Figure S11C). Under H_2_O_2_‐induced oxidative stress conditions, EGFP‐TDP‐43 (green) was partly mislocalized from the nucleus to the cytoplasm of the two cell lines and was pathologically phosphorylated (magenta) (Figure 5C; Figure S11C). Importantly, we found that under pathological stress conditions, wild‐type PDI blocked cytoplasmic mislocalization and antagonized pathological phosphorylation of TDP‐43 in the two cell lines, but dnPDI and PDI Δ*b′* mutant, two negative controls, and abnormal forms of PDI, neither blocked the cytoplasmic mislocalization nor antagonized pathological phosphorylation of TDP‐43 (Figure 5C; Figure S11C). Thus, wild‐type PDI antagonizes H_2_O_2_‐induced pathological phosphorylation of TDP‐43 through the *b′* domain of the enzyme. It should be mentioned that the control results with PDI overexpression but without stress in Figure 5C and Figure S11C represent only a representative result, which shows that without 1 mm stimulation, TDP‐43 and PDI display almost no interaction.

To test whether the basal protein level of PDI could contribute to these effects, we then performed experiments using the inhibition of PDI to complement the overexpression experiments. We obtained confocal images of N2a cells transiently expressing both EGFP‐TDP‐43 and HA‐tagged wild‐type PDI treated with 5 µM PDI inhibitor 16F16 for 16 h and then 1 mm H_2_O_2_ for 1 h (Figure 5C). Under pathological stress conditions combined with the inhibition of PDI activity, more EGFP‐TDP‐43 (green) protein was mislocalized from the nucleus to the cytoplasm of N2a cells and was pathologically phosphorylated (magenta), and the inhibition of PDI activity markedly reduced its ability to antagonize pathological phosphorylation of TDP‐43 in N2a cells, further supporting that wild‐type PDI blocks cytoplasmic mislocalization and antagonizes pathological phosphorylation of TDP‐43 in N2a cells (Figure 5C).

We next performed experiments using knockdown or inhibition of the endogenous PDI to further complement the overexpression experiments. We transiently transfected siRNA targeting the human PDI gene *P4HB* to knock down the expression level of endogenous PDI in the HEK‐293T cell line (Figure 5D,E), thereby investigating the effect of knockdown of the endogenous PDI on pathological phosphorylation of TDP‐43 (Figure 5D–G). Wild‐type HEK‐293T cells, HEK‐293T cells transiently expressing negative control siRNA treated with PBS or 1 mm H_2_O_2_ for 1 h, and HEK‐293T cells transiently expressing PDI siRNA‐1, ‐2, or ‐3 treated with 1 mm H_2_O_2_ for 1 h were used (Figure 5D–G). The cell lysates from the aforementioned cells were probed by anti‐TDP‐43, anti‐pTDP‐43 (pSer409/410), anti‐PDI, and anti‐β‐actin antibodies (Figure 5D). We found that transient transfection of siRNA did not change the total level of endogenous TDP‐43 in HEK‐293T cells (Figure 5D). Under the H_2_O_2_‐induced oxidative stress condition, HEK‐293T cells transiently transfected with PDI siRNA‐1, ‐2, and ‐3 all showed significant decreases in the expression level of PDI (PDI‐siRNA‐1: 0.110 ± 0.012, *p* = 0.00051; PDI‐siRNA‐2: 0.47 ± 0.12, *p* = 0.00603; PDI‐siRNA‐3: 0.37 ± 0.15, *p* = 0.00505) when compared to HEK‐293T cells transiently transfected with negative control siRNA (1.09 ± 0.16), whereas transient transfection of negative control siRNA did not change the expression level of PDI in HEK‐293T cells, indicating that the siRNA used in this study did significantly inhibit the expression of the human PDI gene (Figure 5E). Treatment of H_2_O_2_ significantly promoted pathological phosphorylation of endogenous TDP‐43 in HEK‐293T cells (1.74 ± 0.40 vs. 0.67 ± 0.32, *p* = 0.0226), and importantly, knockdown of the endogenous PDI more significantly promoted H_2_O_2_‐induced pathological phosphorylation of endogenous TDP‐43 in HEK‐293T cells than the control group (PDI‐siRNA‐1: 3.39 ± 0.35 vs. 1.74 ± 0.40, *p* = 0.00573; PDI‐siRNA‐2: 3.99 ± 0.35 vs. 1.74 ± 0.40, *p* = 0.00184; PDI‐siRNA‐3: 4.01 ± 0.72 vs. 1.74 ± 0.40, *p* = 0.00876) (Figure 5D,F). We switched from the anti‐pTDP‐43 (pSer409/410) polyclonal antibody (Cosmo, CAC‐TIP‐PTD‐P07, 1:1000) to the anti‐pTDP‐43 (pS409/410) monoclonal antibody (Proteintech, 80007‐1‐RR,1:1000), which is more suitable for detecting pathological phosphorylation. We used PDI inhibitor 16F16 to reduce the enzymatic activity of endogenous PDI in the HEK‐293T cell line (Figure S11D), thereby investigating the effect of inhibition of the endogenous PDI on pathological phosphorylation of endogenous TDP‐43 (Figure S11D,E). Wild‐type HEK‐293T cells treated with PBS for 1 h, treated with 1 mm H_2_O_2_ for 1 h, and treated with 5 µm PDI inhibitor 16F16 for 16 h and then 1 mm H_2_O_2_ for 1 h were used (Figure S11D,E). The cell lysates from the aforementioned cells were probed by anti‐TDP‐43, anti‐pTDP‐43 (pSer409/410) (the pTDP‐43 Proteintech 80007‐1 monoclonal antibody), anti‐PDI, and anti‐β‐actin antibodies. Treatment of H_2_O_2_ significantly promoted pathological phosphorylation of endogenous TDP‐43 in HEK‐293T cells (0.0767 ± 0.0045 vs. 0.0378 ± 0.0017, *p* = 0.00015), and importantly, inhibition of the endogenous PDI more significantly promoted H_2_O_2_‐induced pathological phosphorylation of endogenous TDP‐43 in HEK‐293T cells than the control group (control + 16F16 + H_2_O_2_: 0.129 ± 0.009 vs. 0.0767 ± 0.0045, *p* = 0.00076) (Figure S11D,E). We show that endogenous PDI, through its specific interaction with endogenous TDP‐43, antagonizes pathological phosphorylation of endogenous TDP‐43 induced by H_2_O_2_ (Figure 5D,F; Figure S11D,E). We then took confocal images of the above experimental groups of HEK‐293T cells, which were doubly immunostained with the anti‐PDI antibody (red) and anti‐pTDP‐43 antibody (magenta) and stained with DAPI (blue) (Figure 5G; Figure S11F). Under the H_2_O_2_‐induced oxidative stress condition, EGFP‐TDP‐43 (green) was partly mislocalized from the nucleus to the cytoplasm of the cells and was pathologically phosphorylated (magenta) (Figure 5G; Figure S11F). Knockdown or inhibition of the endogenous PDI more strongly promoted H_2_O_2_‐induced pathological phosphorylation of TDP‐43 in HEK‐293T cells than the control group (Figure 5G; Figure S11F), further supporting that wild‐type PDI antagonizes pathological phosphorylation of TDP‐43 induced by H_2_O_2_ in HEK‐293T cells. Importantly, knockdown or inhibition of the endogenous PDI significantly reduced not only its protective effect on maintaining nuclear localization of TDP‐43 but also its ability to antagonize pathological phosphorylation of TDP‐43 induced by oxidative stress in HEK‐293T cells (Figure 5D–G; Figure S11F), thereby providing evidence that what we observed could be happening in endogenous conditions. It should be mentioned that the PDI immunofluorescence signal is reduced in the PDI‐siRNA groups compared to the control group (Figure 5G). In the control group, most of the cells have abundant immunofluorescence signal for PDI. In the PDI‐siRNA groups, however, only some cells have abundant immunofluorescence signal for PDI (Figure 5G).

The aforementioned results show a significant dephosphorylation effect of PDI (Figure 5A,D), although the enzyme is not known as a phosphatase. It has been reported that the Tau‐tubulin kinases 1 (TTBK1) directly phosphorylates TDP‐43 and promotes the accumulation of pathological TDP‐43 [68]. Our RT‐qPCR experiments demonstrated that wild‐type PDI significantly inhibited H_2_O_2_‐induced elevation of transcript expression levels of the *TTBK1* gene in HEK‐293T cells, but dnPDI and PDI Δ*b′* mutant, two negative controls, and abnormal forms of PDI did not significantly inhibit H_2_O_2_‐induced elevation of transcript expression levels of the *TTBK1* gene (Figure S11G). Therefore, wild‐type PDI antagonizes pathological phosphorylation of TDP‐43 induced by H_2_O_2_ by inhibiting oxidative stress‐induced elevation of transcript expression levels of the *TTBK1* gene in cells, but not by acting as a phosphatase.

**FIGURE 6:**
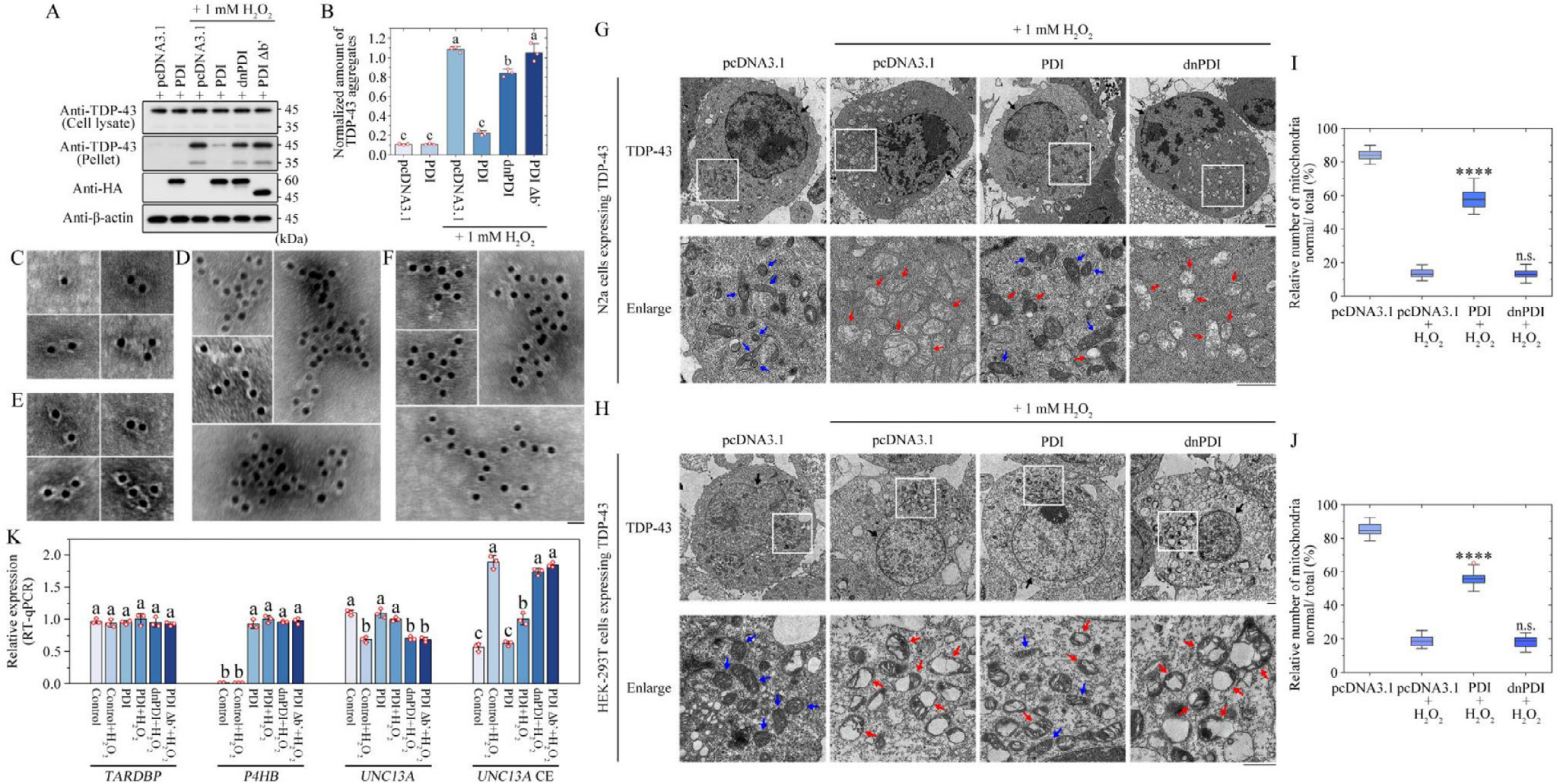
Wild‐type PDI not only antagonizes TDP‐43 pathological aggregates induced by H_2_O_2_ but also suppresses mitochondrial damage and *UNC13A* cryptic splicing in stressed cells through the *b*′ domain of the enzyme. (A) Western blot for endogenous TDP‐43 in the sarkosyl‐insoluble pellets (including insoluble full‐length TDP‐43 and insoluble C‐terminal TDP‐43 fragment of 35 kDa) and the corresponding cell lysates from N2a cells transiently expressing empty vector pcDNA3.1 treated with PBS for 1 h, transiently expressing HA‐tagged wild‐type PDI treated with PBS for 1 h, transiently expressing pcDNA3.1 treated with 1 mm H_2_O_2_ for 1 h, and transiently expressing HA‐tagged wild‐type PDI or HA‐tagged dnPDI or HA‐tagged PDI Δ*b*′ mutant treated with 1 mm H_2_O_2_ for 1 h, using anti‐TDP‐43 antibody. β‐actin served as the protein loading control. (B) The normalized amount of TDP‐43 aggregates in the aforementioned cells (open red circles shown in scatter plots) is expressed as the mean ± SD (with error bars) of values obtained in three independent experiments. Wild‐type PDI significantly antagonizes TDP‐43 pathological aggregates induced by H_2_O_2_, but PDI Δ*b*′ mutant does not significantly antagonize, and dnPDI only mildly antagonizes TDP‐43 pathological aggregates. (C–F) Immunogold electron microscopy of TDP‐43 aggregates purified from the same N2a cells transiently expressing pcDNA3.1 (C, D), wild‐type PDI (E), or dnPDI (F) treated with PBS (C) or 1 mm H_2_O_2_ (D–F) for 1 h as in Figure 5A, and labeled by gold particles conjugated with anti‐TDP‐43 antibody. Scale bar: 20 nm. (G,H) Wild‐type PDI suppressed H_2_O_2_‐induced mitochondrial damage in N2a (G) and HEK‐293T (H) cells stably expressing TDP‐43. TEM images of the stable TDP‐43 overexpressed cell lines, transiently expressing the empty vector pcDNA3.1 treated with PBS for 1 h, transiently expressing the empty vector pcDNA3.1 treated with 1 mm H_2_O_2_ for 1 h, and transiently expressing wild‐type PDI or dnPDI also treated with 1 mm H_2_O_2_ for 1 h. The enlarged regions (bottom) show 14‐fold enlarged images from the top and display the detailed structures of mitochondria in cells. Nuclei and normal mitochondria in cells are highlighted using black arrows and blue arrows, respectively. Abnormal mitochondria in cells with mitochondrial vacuolization are highlighted by red arrows. The samples were negatively stained using 2% uranyl acetate and lead citrate. Scale bars, 1 µm. (I,J) Wild‐type PDI significantly decreased H_2_O_2_‐induced mitochondrial damage in N2a (I) and HEK‐293T (J) cells stably expressing TDP‐43. Box plots analyzing the relative number of mitochondria (normal/total) in the aforementioned cells and showing the quantification of TEM images in *n* = 30 cells examined over 3 independent experiments. The experiments were performed in a blinded manner, and an average of 42 to 57 mitochondria (I) or an average of 19 to 28 mitochondria (J) were present in each cell. The boxes (blue) extend from the 25th to 75th percentiles (quantiles 1 and 3). The minimum, maximum, center, and bounds of the box represent the Q1 – (1.5 × IQR), Q3 + (1.5 × IQR), median (black line), and quantiles 1 and 3, respectively. Wild‐type PDI + H_2_O_2_, *p* = 8.5 × 10^−44^ (I) or 1.1 × 10^−43^ (J); and dnPDI + H_2_O_2_, *p* = 0.973 (I) or 0.982 (J). (I,J) Statistical analyses were performed using a two‐sided Student's *t*‐test. Values of *p *< 0.05 indicate statistically significant differences. The following notation is used throughout: *p < 0.05; **p < 0.01; ***p < 0.001; and ****p < 0.0001 relative to control (pcDNA3.1 + H_2_O_2_ or wild‐type PDI + H_2_O_2_). n.s., not significant. (K) *UNC13A* minigene experiments demonstrated that wild‐type PDI suppressed H_2_O_2_‐induced *UNC13A* cryptic splicing in HEK‐293T cells. Relative transcript expression levels of the human *TARDBP* gene, the human *P4HB* gene, the *UNC13A* gene, and *UNC13A* cryptic exon (*UNC13A* CE) associated with pathological aggregation of TDP‐43 in HEK‐293T cells transiently expressing the empty vector pcDNA3.1 treated with PBS (control) or 1 mm H_2_O_2_ (control + H_2_O_2_) for 1 h, transiently expressing wild‐type PDI treated with PBS (PDI) or 1 mm H_2_O_2_ (PDI + H_2_O_2_) for 1 h, and transiently expressing dnPDI (dnPDI + H_2_O_2_) or PDI Δ*b*′ mutant (PDI Δ*b*′ + H_2_O_2_) also treated with 1 mm H_2_O_2_ for 1 h. The relative transcript expression levels of the four genes in the aforementioned cells are expressed as the mean ± SD (with error bars) of values obtained in three biologically independent experiments. (B,K) Statistical analyses were performed using one‐way ANOVA followed by Bonferroni multiple comparison tests, and different letters indicate significant differences at the level of *p* < 0.05.

The sarkosyl‐insoluble pellets from the same N2a cells as in Figure S8A were probed with anti‐TDP‐43 antibody, and the corresponding cell lysates were probed using anti‐TDP‐43 antibody, anti‐HA antibody, and anti‐β‐actin antibody (Figure 6A). Treatment of H_2_O_2_ strongly promoted pathological aggregation of the endogenous TDP‐43 in N2a cells (Figure 6A). We then compared the amount of endogenous TDP‐43 aggregates (including insoluble full‐length TDP‐43 and insoluble C‐terminal TDP‐43 fragment of 35 kDa in Figure 6A) in N2a cells in the absence and presence of transiently expressed HA‐tagged PDI (Figure 6B). Importantly, we found that under pathological stress conditions, the amount of endogenous TDP‐43 aggregates in N2a cells in the presence of HA‐tagged wild‐type PDI (0.222 ± 0.026) was significantly lower than that in the absence of PDI (1.084 ± 0.028), but PDI Δ*b′* mutant did not significantly change the amount of endogenous TDP‐43 aggregates in N2a cells (1.050 ± 0.094), and HA‐tagged dnPDI only mildly changed the amount of endogenous TDP‐43 aggregates in N2a cells (0.842 ± 0.041) (Figure 6B). Thus, wild‐type PDI antagonizes TDP‐43 pathological aggregates induced by H_2_O_2_ through the b’ domain of the enzyme. To ascertain the nature of oxidative stress‐induced pathological aggregates of TDP‐43 in neuronal cells, we conducted immunogold electron microscopy. The same N2a cells transiently expressing pcDNA3.1 (Figure 6C,D), wild‐type PDI (Figure 6E), or dnPDI (Figure 6F) were treated with PBS (Figure 6C) or with 1 mm H_2_O_2_ (Figure 6D–F) as in Figure 5A, and labeled by gold particles conjugated with anti‐TDP‐43 antibody. The amyloid fibrils in the above cell samples were recognized by anti‐TDP‐43 antibody and decorated with 10‐nm gold labels, and the neuronal cells in the presence of wild‐type PDI produced much less TDP‐43 amyloid fibrils (Figure 6E) than those in the absence of PDI (Figure 6D), suggesting that wild‐type PDI might function as a disaggregase to reverse ALS‐linked TDP‐43 amyloid fibrils. In sharp contrast, dnPDI did not significantly change the amount of TDP‐43 amyloid fibrils (Figure 6F). Thus, wild‐type PDI further blocks pathological aggregation of TDP‐43 induced by H_2_O_2_.

We have shown that dnPDI, a dominant‐negative mutant, interacts with TDP‐43 but does not antagonize pathological phosphorylation and aggregation. These findings suggest that binding alone is not sufficient to counteract pathological phosphorylation and antagonize TDP‐43 pathological aggregates.

### 2.7 Wild‐Type PDI Significantly Decreased H_2_O_2_‐Induced Mitochondrial Damage in Cells Stably Expressing TDP‐43

Given that wild‐type PDI further blocks pathological phosphorylation and aggregation of TDP‐43 induced by H_2_O_2_ (Figures 5, 6A–F; Figure S11), we predicted that wild‐type PDI might suppress TDP‐43 aggregation‐induced mitochondrial impairment. We next used ultrathin section transmission electron microscopy (TEM) [9, 58, 59] to test this hypothesis. To further test the functional relevance of the observations that we made in vitro and in cells with TDP‐43 phase separation, we used N2a and HEK‐293T cells stably expressing TDP‐43 and transiently expressing empty vector pcDNA3.1 treated with PBS, and the stable N2a and HEK‐293T cells transiently expressing pcDNA3.1 treated with 1 mm H_2_O_2_ for 1 h, transiently expressing wild‐type PDI treated with 1 mm H_2_O_2_ for 1 h, and transiently expressing dnPDI also treated with 1 mm H_2_O_2_ for 1 h, using N2a and HEK‐293T cells transiently expressing pcDNA3.1 treated with 1 mm H_2_O_2_ for 1 h as controls (Figure 6G–J). The morphology of mitochondria in the above cells was examined by ultrathin section TEM (Figure 6G,H). The morphology of normal mitochondria in N2a and HEK‐293T cells stably expressing TDP‐43 and transiently expressing pcDNA3.1, which are highlighted by blue arrows, was tubular or round (Figure 6G,H). Treatment of H_2_O_2_ caused severe mitochondrial impairment in N2a and HEK‐293T cells stably expressing TDP‐43, and most of the mitochondria in the cells became swollen and vacuolized, with mitochondrial cristae rupturing and disappearance, which are highlighted by red arrows, and such mitochondrial impairment was caused by TDP‐43 aggregation (Figure 6G,H). Importantly, we found that wild‐type PDI suppressed oxidative stress‐induced mitochondrial damage in N2a and HEK‐293T cells stably expressing TDP‐43 and more than half of the mitochondria in the cells were normal in appearance (highlighted by blue arrows) (Figure 6G,H). By contrast, dnPDI did not suppress oxidative stress‐induced mitochondrial impairment in N2a and HEK‐293T cells stably expressing TDP‐43 (Figure 6G,H). We then compared the number of normal mitochondria in N2a and HEK‐293T cells stably expressing TDP‐43 in the absence and presence of transiently expressed PDI (Figure 6I,J). Importantly, wild‐type PDI significantly decreased H_2_O_2_‐induced mitochondrial damage in N2a and HEK‐293T cells stably expressing TDP‐43 (*p* = 8.5 × 10^−44^ and 1.1 × 10^−43^), but dnPDI did not significantly decrease H_2_O_2_‐induced mitochondrial damage in both cells stably expressing TDP‐43 (*p* = 0.973 and 0.982) (Figure 6I,J). Therefore, wild‐type PDI suppresses mitochondrial impairment induced by TDP‐43 aggregation, possibly via specific interactions with TDP‐43.

Increased levels of oxidative stress are observed in the brain of patients with ALS [66, 67]. We demonstrate that H_2_O_2_ induces TDP‐43 translocation from the nucleus to the cytoplasm (Figure S8), suggesting that oxidative stress may lead to TDP‐43 loss of function. Furthermore, Figures 5 and 6A–F support that H_2_O_2_ induces pathological changes of TDP‐43. It has been reported that pathological changes of TDP‐43 lead to a reduction in UNC13A protein expression and a significant increase in *UNC13A* cryptic exon (*UNC13A* CE) [69, 70]. To validate this more convincingly, we conducted *UNC13A* minigene experiments (Figure 6K). Our *UNC13A* minigene experiments demonstrated that pathological changes of TDP‐43 induced by H_2_O_2_ resulted in a significant reduction in transcript expression levels of the *UNC13A* gene and a more significant increase in *UNC13A* CE than the control group (Figure 6K). These results indicate that *UNC13A* cryptic splicing occurs under H_2_O_2_ stress, thereby confirming that H_2_O_2_ alone is sufficient to cause TDP‐43 loss of function. Importantly, we found that wild‐type PDI significantly suppressed H_2_O_2_‐induced *UNC13A* cryptic splicing in HEK‐293T cells, but dnPDI and PDI Δ*b′* mutant, two negative controls, and abnormal forms of PDI, did not significantly suppress H_2_O_2_‐induced *UNC13A* cryptic splicing (Figure 6K). Therefore, wild‐type PDI not only antagonizes TDP‐43 pathological aggregates induced by H_2_O_2_ but also suppresses mitochondrial damage and *UNC13A* cryptic splicing in stressed cells through the *b′* domain of the enzyme. Since we have shown that wild‐type PDI inhibits TDP‐43 pathology, the aforementioned experiments provide more direct evidence for the regulatory role of PDI in maintaining TDP‐43 function and extend its protective effect from nuclear‐cytoplasmic localization, reduced phosphorylation, and reduced insoluble TDP‐43 to functional preservation.

## 3 Discussion

TDP‐43 dysfunction, characterized by cytoplasmic mislocalization and aggregation of the protein, is not only responsible for neuronal cell death in ALS, FTLD‐TDP, and other TDP‐43 proteinopathies but also observed in the brain of AD patients with TDP‐43 pathology [1, 2, 3, 4, 5, 6, 10, 11, 12, 13, 14, 15, 16, 17, 18, 71]. In this study, we detected a much higher level of cytoplasmic TDP‐43 in ALS and AD‐TDP brains compared to healthy, Castleman disease, and PSP controls. Accumulating pieces of evidence point to a crucial role of oxidative stress in ALS pathogenesis [50, 51, 66, 67]. Here we show that oxidative stress causes TDP‐43 to mislocalize and accumulate in the cytoplasm of neuronal cells; promotes subsequent pathological phosphorylation, fragmentation, and aggregation of TDP‐43; and causes severe mitochondrial impairment in ALS. Moreover, we show that these effects are mediated, at least in part, by direct interactions of TDP‐43 with PDI.

Several in vivo co‐IP studies have described physical interactions between TDP‐43 and different classes of proteins in neurons and neuronal cells, among which amyloid‐β, Rho guanine nucleotide exchange factor, Hsp90 and its co‐chaperone Sti1, histone deacetylase 1 and Hsp70, and Grp78, are highlighted as potential TDP‐43 partners [14, 72, 73, 74, 75]. PDI is partly mislocalized from the ER to the cytoplasm under ER stress [22, 23, 24, 25, 26, 27, 28, 29, 30], and wild‐type PDI plays important roles in preventing protein aggregation in ALS pathogenesis [22, 23, 25]. Our study indicates that PDI regulates pathological functions of cytoplasmic TDP‐43 in neurons via multiple mechanisms, the first being physical interaction with TDP‐43. Using human brain samples, cell culture models, biochemical reconstitution assays, and imaging, we report that PDI binds to TDP‐43, suppresses its phase separation, disassembles TDP‐43/G3BP1 stress granules, and counteracts TDP‐43 phosphorylation, aggregation, and associated mitochondrial toxicity.

Our study also advances the understanding of the role of phase separation of proteins in neurodegenerative diseases. We show that TDP‐43 undergoes phase separation in vitro and within stressed cells and that recruitment of wild‐type PDI strongly impedes such phase separation. In contrast, recruitment of an abnormal form of PDI mediates TDP‐43 condensation, rendering the resulting liquid droplets fibril‐like. The recruitment of wild‐type PDI into TDP‐43 condensates weakens the interaction between TDP‐43 and G3BP1 under stress, reducing TDP‐43 condensation and aggregation. It has been reported that the in vivo phase separation of TDP‐43 is modulated by two types of molecular chaperones (Hsp70 and HSPB1) [39, 41]. Intriguingly, TDP‐43 condensates selectively recruit HSPB1 in cells upon stress, which in turn inhibits TDP‐43 assembly into fibrils, and the combined activities of HSPB1, BAG2, and HSPA1A facilitate the disassembly of TDP‐43 condensates within stressed cells [41]. It should be mentioned that experimental disruption of G3BP1 dimerization, ubiquitination of G3BP1, and two G3BP inhibitors all result in the disassembly of protein condensates in cells [61, 62, 64]. Thus, the specific properties of proteins included in TDP‐43 condensates influence the liquidness and organization of TDP‐43 condensates (this work) [39, 41], and targeting of the core protein G3BP1 weakens the stress granule–specific interactions, resulting in the disassembly of protein condensates in cells (this work) [62, 64].

**FIGURE 7:**
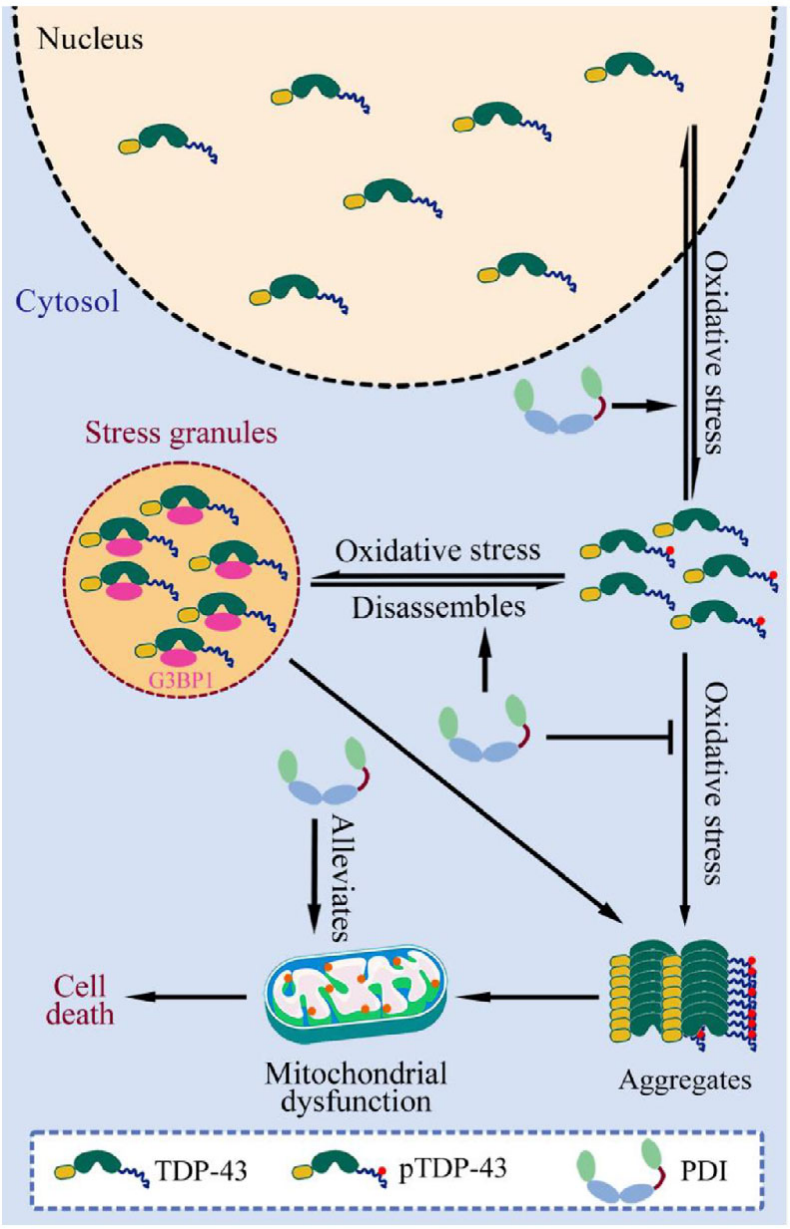
A hypothetical model shows how wild‐type PDI disassembles TDP‐43/G3BP1 condensates and antagonizes TDP‐43 pathological aggregates. Under oxidative stress conditions, TDP‐43 (N‐terminal domain, yellow; C‐terminal domain, ultramarine; and two RNA recognition motifs, dark green) is mostly mislocalized from the nucleus (light yellow) to the cytosol (pale blue) and is pathologically phosphorylated (red), but wild‐type PDI (domains *a* and *a′*, green; domains *b* and *b′*, blue; and the x linker, dark red) blocks cytoplasmic mislocalization and pathological phosphorylation of TDP‐43 (pTDP‐43). Then, TDP‐43 selectively interacts with G3BP1 (magenta ellipsoid) to assemble stress granules (orange balls), but wild‐type PDI disassembles TDP‐43/G3BP1 condensates via specific interactions with TDP‐43. Finally, pTDP‐43 and stress granules are assembled into amyloid fibrils, resulting in mitochondrial dysfunction and neuronal cell death in patients with ALS and AD‐TDP, but wild‐type PDI antagonizes pathological aggregation of TDP‐43, suppresses mitochondrial dysfunction and TDP‐43 toxicity, and maintains TDP‐43 function.

In summary, our results underpin a model of how wild‐type PDI disassembles TDP‐43/G3BP1 condensates, antagonizes TDP‐43 pathological aggregates, and suppresses mitochondrial dysfunction in ALS (Figure 7). Under pathological stress conditions, TDP‐43, the major component of pathological inclusions in most patients with ALS and FTLD‐TDP and in up to 57% of patients with AD [1, 2, 3, 4, 5, 6, 10, 11, 12, 13, 14, 15, 16, 17, 18], is partly mislocalized from the nucleus to the cytoplasm of neuronal cells, is pathologically phosphorylated, and selectively interacts with wild‐type PDI; this interaction in turn blocks cytoplasmic mislocalization and antagonizes pathological phosphorylation of TDP‐43 (Figure 7). Then, TDP‐43 selectively interacts with G3BP1 to assemble TDP‐43 condensates, but wild‐type PDI competes with G3BP1 and disassembles TDP‐43 condensates via specific interactions with TDP‐43 (Figure 7). In response to oxidative stress, TDP‐43 recruits and interacts with proteins of the ER and endosomal‐extracellular transport pathways [75], such as wild‐type PDI (this work). Finally, in the presence of abnormal forms of PDI, PDI loses its activity, pTDP‐43 and stress granules are assembled into amyloid fibrils, resulting in mitochondrial dysfunction and neuronal cell death in patients with ALS and AD‐TDP, but wild‐type PDI clears pathological insoluble TDP‐43, suppresses mitochondrial dysfunction and TDP‐43 toxicity, and maintains TDP‐43 function (Figure 7). Because PDI has such a strong effect on clearing pathological insoluble TDP‐43, we plan to use a CRISPR activation system to specifically activate endogenous PDI expression in the near future. Moreover, this finding has potential therapeutic significance, and phase separation alone may not be sufficient to explain such a remarkable effect. Therefore, accumulation or overexpression of cytoplasmic TDP‐43 is toxic to neurons via enhancing not only TDP‐43/G3BP1 condensates but also pathological phosphorylation and aggregation of TDP‐43 under oxidative stress conditions. We show that only wild‐type PDI disassembles TDP‐43/G3BP1 condensates and antagonizes TDP‐43 pathological aggregates, indicating the contribution of the enzyme activity of PDI. Full‐length TDP‐43 is a target protein for PDI. Very recently, Yan and co‐workers reported in *Cell* that under oxidative stress conditions, the exposed Cys173 and Cys175 in the RNA‐recognition motif 1 (RRM1) likely favor disulfide bond formation of TDP‐43 and thereby favor TDP‐43 condensation [48]. These results provide insights on the regulation of PDI on pathological functions of cytoplasmic TDP‐43 via phase separation of the protein inhibited by wild‐type PDI, but not abnormal forms of PDI, which has important implications in ALS etiology. The observation of many colocalized dots of TDP‐43 and PDI in the cytoplasm of a group of ALS patients and AD patients provides clinical relevance. The selective interaction of wild‐type PDI with TDP‐43 upon stress will be valuable to understanding the functional basis underlying phase separation of proteins and inspiring future research on protein condensation diseases caused by abnormal liquid‐like or solid‐like states of proteins [76] and regulated by protein interaction partners [39, 41, 77].

## 4 Experimental Section

### 4.1 Ethics Statement

This study complied with all relevant ethical regulations and is based on analyses of brain samples from two patients with amyotrophic lateral sclerosis, six patients with Alzheimer's disease, three healthy individuals, one patient with Castleman disease, and one patient with progressive supranuclear palsy. The patient characteristics are described in Table 1. Postmortem human brain tissue samples were obtained from the National Human Brain Bank for Development and Function, Institute of Basic Medical Sciences Chinese Academy of Medical Sciences, School of Basic Medicine Peking Union Medical College (Case 1 and Control 4), the Department of Neurology, Peking University Third Hospital (Case 2), and the Department of Neurology, Xiangya Hospital, Central South University (Cases 3 to 8, and Controls 1 to 3 and 5). Their use in this study was approved by the ethical review processes at each institution, including ethical approval from relevant authorities at Wuhan University. Informed consent was obtained from the patients or their next of kin. We obtained consent to publish information that identified individuals (including three or more indirect identifiers such as exact age, sex, and medical center the study participants attended or rare diagnoses). The studies were approved by the Wuhan University Ethics Committee (WHU‐LFMD‐2024100).

### 4.2 Double Immunofluorescence Staining of Pathological Samples

Sections of the precentral gyrus (Case 1, Case 2, and Control 4), substantia nigra (Case 2), and hippocampus and temporal lobe (Cases 3‐8 and Controls 1‐3, 5) were formalin‐fixed and paraffin‐embedded for in situ staining. The 5 µm paraffin‐embedded sections were cut, deparaffinized in xylene, and rehydrated in a descending series of ethanol. Antigen retrieval was carried out using an Improved Citrate Antigen Retrieval Solution (Beyotime, P0083) at 95°C–100°C for 20 min, after which the samples were cooled to room temperature. After being washed three times with PBS for 3 min, the sections were marked with hydrophobic circles using an immunohistochemical pen. After being permeabilized with cold 0.3% Triton X‐100 in PBS at room temperature for 15 min, the sections were blocked with 5% BSA at room temperature for 1 h, followed by overnight incubation with primary antibodies, diluted in PBS containing 5% BSA in a humidified chamber at 4°C. After three washes with PBS for 5 min, fluorescence‐conjugated secondary antibodies were applied to the cover slip and incubated in a dark room for 1 h. DAPI was then applied at the proper dilution; after being washed three times with PBS for 5 min and mounted with antifade mounting medium (Beyotime, P0126), the samples were subjected to a Leica TCS SP8 laser scanning confocal microscope (Wetzlar, Germany). The following primary antibodies were used: rabbit anti‐TDP‐43 polyclonal antibody (Proteintech, 10782‐2‐AP, 1:200), mouse anti‐P4HB monoclonal antibody (anti‐PDI antibody, Abcam, ab2792, 1:200), rabbit anti‐ER polyclonal antibody (Proteintech, 21244‐1‐AP, 1: 100), mouse anti‐G3BP1 monoclonal antibody (Proteintech, 66486‐1‐Ig, 1: 200), normal rabbit IgG (Beyotime, A7016, 1: 200), normal mouse IgG (Beyotime, A7028, 1: 200), Alexa Fluor 488‐labeled goat anti‐rabbit IgG (H + L) (Beyotime, A0423, 1: 500), and Alexa Fluor 555‐labeled donkey anti‐mouse IgG (H + L) (Beyotime, A0460, 1: 500).

### 4.3 Protein Purification

The plasmid‐encoding human full‐length TDP‐43 was a kind gift from Dr. H.‐N. Du (College of Life Sciences, Wuhan University). A recombinant, full‐length wild‐type human TDP‐43 (residues 1–414) containing a tag of six histidine residues (polyhistidine tag) at its C‐terminal domain was constructed into the prokaryotic expression vector pET22b and expressed in *Escherichia coli* (*E. coli*) BL21 (DE3) cells (Novagen, Merck, Darmstadt, Germany). The TDP‐43 protein was purified to homogeneity by nickel affinity chromatography as described by Vega et al. [78]. After purification, the refolded TDP‐43 protein was dialyzed against 50 mm Tris‐HCl buffer (pH 8) containing 150 mm NaCl four times to remove the detergents and salts, concentrated, and then centrifuged at 17 000 *g* for 30 min at 4°C to remove large aggregates. The supernatant of the refolded TDP‐43 protein was freshly used or stored at −80°C. SDS‐PAGE was used to confirm that the purified human TDP‐43 protein was present as a single protein. We used a BCA protein assay kit (Beyotime, P0012) to determine the concentration of the human TDP‐43 protein.

A plasmid‐encoding human full‐length PDI was a kind gift from Dr. L. W. Ruddock (Faculty of Biochemistry and Molecular Medicine, University of Oulu). The gene for PDI 1‐491 was constructed in the prokaryotic expression vector pET23, and the PDI mutants dnPDI and PDI Δ*b′* were constructed by site‐directed mutagenesis using a wild‐type PDI template; the primers used are listed in Table S1. All PDI plasmids were transformed into *E. coli*. Recombinant full‐length wild‐type human PDI (residues 1–491) and its variants dnPDI and PDI Δ*b′* were expressed from the vector pET23 in *E. coli* BL21 (DE3) Codon plus‐RIL cells (Novagen, Merck, Darmstadt, Germany). PDI proteins were purified to homogeneity by nickel affinity chromatography as described by Wang et al. [79]. The eluted fractions were dialyzed against 50 mm Tris‐HCl buffer (pH 8) containing 150 mm NaCl twice to remove EDTA. The PDI proteins were freshly used or stored at −80°C. SDS‐PAGE was used to confirm that the purified human PDI proteins were from a single species. We used a UV‐2550 Probe spectrophotometer (Shimadzu, Kyoto, Japan) to determine the concentrations of wild‐type human PDI, dnPDI, and PDI Δ*b′* using their absorbances at 214 nm with a standard calibration curve drawn from BSA.

A plasmid‐encoding human full‐length G3BP1 was a kind gift from Dr. Y. Liu (College of Life Sciences, Wuhan University). A recombinant, full‐length wild‐type human G3BP1 (residues 1–466) containing a tag of six histidine residues (polyhistidine tag) at its C‐terminal domain was constructed into the prokaryotic expression vector pET28a and expressed in *E. coli* BL21 (DE3) cells (Novagen, Merck, Darmstadt, Germany). The G3BP1 protein was purified to homogeneity by nickel affinity chromatography. In brief, the cells were grown at 37°C until the optical density at 600 nm reached 0.6, induced with 1 mM IPTG and further grown at 16°C for 16 h. The cells were subsequently harvested by centrifugation (12 000 *g* for 5 min) and resuspended in 20 mm Na_3_PO_4_ buffer (pH 7.3) containing 1 mm DTT and 1 mm PMSF. The cell suspension was lysed by ultrasonication for 30 min on ice and then centrifuged at 17 000 *g* for 30 min at 4°C. The supernatant was loaded onto a nickel affinity column and preequilibrated with 20 mm Na_3_PO_4_ buffer (pH 7.3) at a flow rate of 1 mL/min. After loading, the column was washed with 20 mm Na_3_PO_4_ buffer (pH 7.3) and 20 mm Na_3_PO_4_ buffer (pH 7.3) containing 50 mm imidazole. Finally, the protein was eluted with 20 mm Na_3_PO_4_ buffer (pH 7.3) containing 300 mm imidazole. The purified protein was analyzed by SDS‐PAGE and dialyzed against 50 mm Tris‐HCl buffer (pH 8) containing 150 mm NaCl twice to remove imidazole. The concentration of G3BP1 was determined according to its absorbance at 214 nm with a standard calibration curve drawn from BSA. The G3BP1 protein was freshly used or stored at −80°C.

### 4.4 Biotin Pull‐Down Assay

For biotin labeling, the freshly bacterial‐purified wild‐type human TDP‐43 was incubated with biotin‐NHS ester (MCE, HY‐D0802) at a TDP‐43: biotin‐NHS ester molar ratio of 1: 20 at 4°C for 2 h. Free biotin‐NHS was removed by ultrafiltration using a 10K centrifugal filter (Millipore).

For the biotin pull‐down assay, biotin or biotin‐labeled TDP‐43 were incubated with streptavidin magnetic beads (Beyotime; P2151) at 4°C for at least 4 h. After being bound to streptavidin magnetic beads, the probe‐beads were incubated with wild‐type PDI, dnPDI, or PDI Δ*b′* overnight at 4°C. After washing with ice‐cold PBS containing 1% (v/v) NP‐40 at least 15 times, the proteins pulled down by the probed‐coated beads were analyzed by Western blot. The following antibodies were used: rabbit anti‐TDP‐43 polyclonal antibody (Proteintech, 10782‐2‐AP, 1: 2000), mouse anti‐P4HB monoclonal antibody (anti‐PDI antibody, Abcam, ab2792, 1: 1000). Pre‐clearing the wild‐type PDI, dnPDI, or PDI Δ*b′* samples with streptavidin magnetic beads was necessary to reduce non‐specific binding before the binding complex was assembled.

### 4.5 Characterization of SNO‐PDI by Fluorescence Spectroscopy

S‐nitrosocysteine was synthesized as described in detail below. Cysteine was dissolved in 0.625 m HCl at 0°C to a final concentration of 625 mm. An equimolar amount of NaNO_2_ was added, and the mixture was stirred at 0°C for 40 min. A 2.5‐fold volume of acetone was then added, and the new mixture was stirred at 0°C for 20 min, followed by filtration of the precipitate. The S‐nitrosocysteine solution was washed once with 80% acetone, twice with 100% acetone, three times with diethyl ether, and finally, the solution was freeze‐dried. To produce SNO‐PDI, wild‐type PDI and S‐nitrosocysteine were cultured at a molar ratio of 1:100, incubated for 60 min in the dark at 4°C, concentrated, and washed with 50 mm Tris‐HCl buffer (pH 8) containing 150 mm NaCl to remove residual S‐nitrosocysteine using centrifugal filters. SNO‐PDI was then assessed by the release of NO, which causes the conversion of 2,3‐diaminonaphthalene (DAN) into the fluorescent compound 2,3‐naphthyltriazole (NAT). In brief, Tris‐HCl buffer (pH 8) (control) and Tris‐HCl buffer (pH 8) containing wild‐type PDI or purified SNO‐PDI were incubated with a solution containing 100 µm DAN and 100 µm CuSO_4_ in the dark at room temperature for 30 min. Then, 1 m NaOH was added to each tube to stop the reaction. The NO‐dependent S‐nitrosylation of DAN to yield its highly fluorescent S‐nitrosylated derivative, NAT, was quantified by measuring the fluorescence of each sample using an excitation wavelength of 375 nm and an emission wavelength of 450 nm. In other words, the fluorescence intensity of NAT in the above samples was measured using excitation at 375 nm and emission at 450 nm.

### 4.6 Liquid‐Droplet Formation

The freshly bacterial‐purified wild‐type human TDP‐43 was incubated with 5(6)‐TAMRA SE (Shanghai Dibai, 246256‐50‐8, red fluorescence, excitation at 552 nm) at a TDP‐43: TAMRA molar ratio of 1: 3 in the dark at 4°C for 1 h; the freshly bacterial‐purified wild‐type human PDI and two abnormal forms of PDI (dnPDI and SNO‐PDI), along with BSA, a negative control, were incubated with 5(6)‐FITC (Shanghai Dibai, 27072‐45‐3, green fluorescence, excitation at 488 nm) at a PDI/BSA: FITC mass ratio of 1: 50–80 in the dark at 4°C for at least 8 h; the freshly bacterial‐purified wild‐type human G3BP1 was incubated with Cy5‐SE (MCE, 146368‐14‐1, magenta fluorescence, excitation at 638 nm) at a G3BP1:CY5 molar ratio of 1:10 in the dark at 4°C for 1 h. These labeled proteins were filtered, concentrated in a centrifugal filter (Millipore), and diluted in 50 mm Tris‐HCl buffer (pH 8) containing 150 mm NaCl. To block free cysteine thiols in TDP‐43, the freshly bacterial‐purified wild‐type human TDP‐43 was incubated with 20 mm iodoacetamide (IAM) (MCE, HY‐34477) at 4°C for 1 h, and BeyoDesalt G‐25 Spin (Beyotime, P2613) was used to remove residual IAM. TDP‐43‐IAM was then assessed by Total Thiol Assay Kit with DTNB (Beyotime, S0141) following the manufacturer's protocol.

Next, 10 µm TDP‐43 labeled with TAMRA was incubated with Tris‐HCl buffer (pH 8) containing 150 mm NaCl and 10% (w/v) PEG 3350 or with the same buffer supplemented with 10 µm wild‐type PDI, dnPDI, or SNO‐PDI labeled with FITC on ice to induce phase separation, and FITC‐labeled BSA served as a control. TDP‐43 demixed droplets (red) coacervated with PDI or BSA labeled with FITC (green) were observed by a Leica TCS SP8 laser scanning confocal microscope (Wetzlar, Germany) with excitation at 552 and 488 nm, respectively. In total, 2.5, 5, 7.5, or 10 µm TDP‐43 labeled with TAMRA was incubated with Tris‐HCl buffer (pH 8) containing 150 mm NaCl and 10% PEG 3350 or with the same buffer supplemented with 10 µm wild‐type PDI, dnPDI, or SNO‐PDI on ice to induce phase separation. Liquid droplets of TDP‐43 were observed by a Leica TCS SP8 laser scanning confocal microscope (Wetzlar, Germany) with excitation at 552 nm. TDP‐43 or TDP‐43‐IAM (10 µm) labeled with TAMRA were incubated with Tris‐HCl buffer (pH 8) containing 150 mm NaCl and 10% (w/v) PEG 3350 or with the same buffer supplemented with 10 µm wild‐type PDI or dnPDI on ice to induce phase separation. Condensates of TDP‐43 were observed by a Leica TCS SP8 laser scanning confocal microscope (Wetzlar, Germany) with excitation at 552 nm.

TDP‐43 (10 µm) labeled with TAMRA and 5 µm G3BP1 labeled with CY5 were incubated with Tris‐HCl buffer (pH 8) containing 150 mm NaCl and 10% (w/v) PEG 3350 or with the same buffer supplemented with 10 µm wild‐type PDI or dnPDI labeled with FITC on ice to induce phase separation. Co‐condensates of G3BP1 and TDP‐43 coacervated with PDI were observed by a Leica TCS SP8 laser scanning confocal microscope (Wetzlar, Germany) with excitation at 638, 552, and 488 nm, respectively. All phase separation experiments were performed at least three times and were reproducible.

### 4.7 Turbidity Assays

In total, 0.50, 1, 2, 3, 4, 5, 6, 7, 8, 9, and 10 µm wild‐type human TDP‐43) were incubated with Tris‐HCl buffer (pH 8) containing 150 mm NaCl and 10% PEG 3350 or incubated with the same buffer further containing 10 µm wild‐type human PDI, dnPDI, or SNO‐PDI on ice to induce phase separation. The turbidity of TDP‐43 condensates was measured at 600 nm and 25°C using a NanoDrop OneC Microvolume UV–vis Spectrophotometer (Thermo Fisher Scientific), and turbidity changes for TDP‐43 phase separation in the absence of PDI were used as controls. All turbidity assays were repeated at least three times and were reproducible.

### 4.8 Fluorescence Recovery After Photobleaching (FRAP)

In total, 10 µm wild‐type human TDP‐43 labeled with TAMRA was incubated with Tris‐HCl buffer (pH 8) containing 150 mm NaCl and 10% (w/v) PEG 3350 or with the same buffer containing 10 µm wild‐type human PDI, dnPDI, or SNO‐PDI on ice to induce phase separation. For co‐condensates of G3BP1 and TDP‐43, 10 µm TAMRA‐labeled wild‐type human TDP‐43 and 5 µm G3BP1) were incubated with Tris‐HCl buffer (pH 8) containing 150 mm NaCl and 10% (w/v) PEG 3350 or with the same buffer containing 10 µm wild‐type human PDI or dnPDI on ice to induce phase separation. Liquid droplets were observed by a Zeiss LSM 880 laser scanning confocal microscope equipped with an Airyscan detector (Carl Zeiss, Germany) with excitation at 561 nm. We chose TDP‐43 droplets with a size of ∼5 µm for FRAP experiments. For each droplet, a square was bleached at 70%–80% transmission once, and postbleaching time‐lapse images were collected (250 or 200 frames, 1000 ms per frame). Images were analyzed using Zen (LSM 880 confocal microscope manufacturer's software). All FRAP experiments were repeated three times, and the results were reproducible.

### 4.9 PDI siRNA

Three different PDI siRNAs were used for the experiments together with a negative control siRNA. PDI siRNA‐1: 5’‐ GCAAGAUCCUGUUCAUCUU(dT)(dT)‐3’; PDI siRNA‐2: 5’‐CUGCAAAACUGAAGGCAGAUU‐3’; PDI siRNA‐3: 5’‐CCUUUGCUAGCGAAUCUCAGAGCC‐3’. All siRNAs were synthesized by Tsingke (China).

### 4.10 Cell Culture and Transfection

N2a neuroblastoma cells (RRID: CVCL 0470; catalog number GDC0162) and HEK‐293T cells (RRID: CVCL 0063; catalog number GDC0187) were purchased from the China Center for Type Culture Collection (CCTCC, Wuhan, China) on March 2, 2022 and January 12, 2022, respectively, and cultured in minimum essential media and Dulbecco's modified Eagle's medium (Gibco, Invitrogen), respectively, supplemented with 10% (v/v) fetal bovine serum (Gibco), 100 U/mL streptomycin, and 100 U/mL penicillin in 5% CO_2_ at 37°C. We hereby confirm that the two cell lines were contamination‐free (see “Register of Misidentified Cell Lines, International Cell Line Authentication Committee”). All the plasmids and siRNA transient transfections were performed using Lipofectamine 2000 (Invitrogen) according to the manufacturer's instructions. In brief, after one day of culture, 2 µg of plasmid or 500 nmol of siRNA and 5 µL of Lipofectamine 2000 (Invitrogen) were diluted in 200 µL of Opti‐MEM (Gibco) in each well of a 6‐well plate, incubated in 5% CO_2_ at 37°C for 4–6 h, and then cultured in proper cell culture medium for 36–48 h. N2a or HEK‐293T cells stably expressing wild‐type human TDP‐43 were generated with a lentiviral vector construction system (phage‐puro). The target DNA fragments were inserted into the lentiviral vector, and plasmids containing target DNA, pVSVG, and p976 were packaged in HEK‐293T cells at a ratio of 2:1:1 by Lipofectamine 2000 (Invitrogen). The ratio of liposomes to DNA was 2:1. After 48 h of transfection, the viruses were harvested and filtered, and then N2a or HEK‐293T cells were infected with the packaged lentivirus twice for 12 h each at 12‐h intervals. To establish stable cell lines, puromycin was used to screen for overexpressed cells. The expression of each protein was detected by Western blot. Western blot analysis with the anti‐TDP‐43 antibody confirmed that the stable TDP‐43 overexpressing cell lines exhibited an increase in the abundance of TDP‐43 compared with the controls.

### 4.11 Western Blotting

For analysis by Western blotting, N2a and HEK‐293T cells grown in a 6‐well plate for one day were transfected and treated as indicated. The cells were washed twice with ice‐cold PBS and lysed in 300 µL (per well) of cell lysis buffer containing 1× protease inhibitor cocktail (Target Mol). The amount of loaded protein was normalized using a BCA protein quantification kit (Beyotime, P0012). The cell lysates were boiled in SDS‐PAGE loading buffer for 10 min and then subjected to SDS‐PAGE and probed with the following specific antibodies: mouse anti‐TDP‐43 monoclonal antibody (Abcam, ab57105,1:1000), rabbit anti‐TDP‐43 polyclonal antibody (Proteintech, 10782‐2‐AP, 1: 2000), rabbit anti‐pTDP‐43 (pS409/410) polyclonal antibody (Proteintech, 22309‐1‐AP,1: 1000), rabbit anti‐pTDP‐43 (pS409/410) monoclonal antibody (Proteintech, 80007‐1‐RR,1: 1000), mouse anti‐P4HB monoclonal antibody (anti‐PDI antibody, Abcam, ab2792, 1:1000), rabbit anti‐HA tag polyclonal antibody‐ChIP Grade (Abcam, ab9110, 1: 1000), mouse anti‐HA tag monoclonal antibody (Proteintech, 66006‐2‐Ig, 1: 5000), rabbit anti‐Histone‐H3 polyclonal antibody (Proteintech, 17168‐1‐AP, 1: 1000), mouse anti‐G3BP1 monoclonal antibody (Proteintech, 66486‐1‐Ig, 1: 5000), mouse anti‐β‐actin monoclonal antibody (Proteintech, 66009‐1‐Ig, 1: 5000), HRP‐labeled goat anti‐rabbit IgG (H+L) (Beyotime, A0208, 1: 5000), and HRP‐labeled goat anti‐mouse IgG (H+L) (Beyotime, A0216, 1: 5000).

### 4.12 Coimmunoprecipitation

To confirm the interaction and interaction region between TDP‐43 and PDI, cells were transfected as indicated according to the manufacturer's instructions. After one day of culture and 1 h of treatment with PBS or 1 mm H_2_O_2_ at 37°C, the cells were harvested by centrifugation at 100 *g* at 4°C for 5 min and ruptured on ice for 20 min with cell lysis buffer containing 1× protease inhibitor cocktail (Target Mol). The cell lysates were then centrifuged at 17 000 *g* at 4°C for 20 min to remove cell debris. The collected supernatant was set aside for subsequent analysis. Each sample was divided and immunoprecipitated with 1 µL of 1 mg/mL mouse anti‐TDP‐43 antibody or mouse IgG on a rotator overnight at 4°C. The complexes were pulled down by incubation with Protein G Agarose beads (20 µL, Fast Flow for IP) (Beyotime) on a rotator at 4°C for 4 h. The beads were washed five times with PBS, boiled in SDS‐PAGE loading buffer for 10 min, subjected to SDS‐PAGE, and then subjected to Western blot using a rabbit anti‐HA antibody. Nonspecific IgG served as a negative control for immunoprecipitation. Other reserved cell lysates were boiled in SDS‐PAGE loading buffer, probed by Western blotting using anti‐HA, anti‐TDP‐43, and anti‐β‐actin antibodies, respectively, and served as the input controls, which represented the total PDI, dnPDI, or PDI Δ*b′* content, the total TDP‐43 content, and the total protein content in cell lysates, respectively. The same groups treated with PBS were used as negative controls. The following antibodies were used: mouse anti‐TDP‐43 monoclonal antibody (Abcam, ab57105,1: 1000), rabbit anti‐HA tag polyclonal antibody‐ChIP Grade (Abcam, ab9110, 1: 1000), mouse anti‐β‐actin monoclonal antibody (Proteintech, 66009‐1‐Ig, 1: 5000), HRP‐labeled goat anti‐rabbit IgG (H+L) (Beyotime, A0208, 1: 5000), and HRP‐labeled goat anti‐mouse IgG (H+L) (Beyotime, A0216, 1: 5000).

To confirm the disassembly of stress granules, N2a and HEK‐293T cells grown in a 6‐well plate were transfected and treated as indicated. After one day of culture and 1 h of treatment with 1 mm H_2_O_2_ or PBS at 37°C, the cells were harvested by centrifugation at 100 *g* at 4°C for 5 min and ruptured on ice for 20 min with cell lysis buffer containing 1× protease inhibitor cocktail (Target Mol). The cell lysates were then centrifuged at 17 000 *g* at 4°C for 20 min to remove cell debris. The collected supernatant was set aside for subsequent analysis. Each sample was divided and immunoprecipitated with 1 µL of 1 mg/mL rabbit anti‐TDP‐43 antibody or rabbit IgG on a rotator overnight at 4°C. The complexes were pulled down by incubation with Protein G Agarose beads (20 µL, Fast Flow for IP) (Beyotime) on a rotator at 4°C for 4 h. The beads were washed five times with PBS, boiled in SDS‐PAGE loading buffer for 10 min, subjected to SDS‐PAGE, and then subjected to Western blotting using a mouse anti‐G3BP1 antibody. Nonspecific IgG served as a negative control for immunoprecipitation. Other reserved cell lysates were boiled in SDS‐PAGE loading buffer, probed by Western blotting using anti‐G3BP1, anti‐HA, anti‐TDP‐43, and anti‐β‐actin antibodies, which served as the input controls and represented the total G3BP1 content, the total PDI or dnPDI content, the total TDP‐43 content, and the total protein content in cell lysates, respectively. The following antibodies were used: rabbit anti‐TDP‐43 polyclonal antibody (Proteintech, 10782‐2‐AP, 1:2000), mouse anti‐HA tag monoclonal antibody (Proteintech, 66006‐2‐Ig, 1: 5000), mouse anti‐G3BP1 monoclonal antibody (Proteintech, 66486‐1‐Ig, 1: 5000), mouse anti‐β‐actin monoclonal antibody (Proteintech, 66009‐1‐Ig, 1: 5000), HRP‐labeled goat anti‐rabbit IgG (H + L) (Beyotime, A0208, 1: 5000), and HRP‐labeled goat anti‐mouse IgG (H+L) (Beyotime, A0216, 1: 5000).

### 4.13 Bimolecular Fluorescence Complementation Assay

The human *TARDBP* gene was cloned and inserted into the pEGFP‐N1 vector, the fusion gene was truncated at amino acid 172 of EGFP using a stop codon, and the pEGFP‐TDP‐43‐GN172 vector was subsequently obtained. In addition, the human *P4HB* gene was cloned and inserted into the pcDNA3.1 vector, the fused HA‐tag and EGFP_155‐238_ genes were inserted into the *P4HB* gene between the signal peptide and the amino acid coding sequence, and the HA‐tagged pcDNA3.1‐PDI‐GC155 vector was subsequently obtained. The pcDNA3.1‐dnPDI‐GC155 vector was obtained by mutating four cysteines (Cys53, Cys56, Cys397, and Cys400) of pcDNA3.1‐PDI‐GC155 to Ala. The fusion expression vectors were confirmed by DNA sequencing, and the expression of the fusion proteins was detected by Western blotting. One day before transfection, HEK‐293T cells were plated on glass‐bottom culture dishes. The cells were then transiently co‐transfected with pEGFP‐TDP‐43‐GN172 and pcDNA3.1‐PDI‐GC155 or pcDNA3.1‐dnPDI‐GC155 according to the manufacturer's instructions. After one day of culture and 1 h of treatment with 1 mm H_2_O_2_, living cells were stained with Hoechst 33342 (*blue*) and ER Staining Kit (*red*) (Abcam, ab139482), and were observed by a Leica TCS SP8 laser scanning confocal microscope (Wetzlar, Germany) with excitation at 405, 561, and 488 nm, respectively. Confocal microscopy was also used to visualize the fluorescence of the following control cells: living HEK‐293T cells transiently expressing both the pEGFP‐TDP‐43‐GN172 and pcDNA3.1‐PDI‐GC155 or pcDNA3.1‐dnPDI‐GC155 constructs without H_2_O_2_ treatment; living HEK‐293T cells transiently expressing both the pEGFP‐TDP‐43‐GN172 and pcDNA3.1‐GC155 constructs; and cells transiently expressing both the pEGFP‐GN172 and pcDNA3.1‐PDI‐GC155, pcDNA3.1‐dnPDI‐GC155, or pcDNA3.1‐GC155 constructs treated with 1 mm H_2_O_2_ for 1 h.

### 4.14 Nuclear and Cytoplasmic Protein Fractionation

N2a cells grown in a 6‐well plate for the day were transfected as indicated. Cells were washed three times with ice‐cold PBS and lysed in 300 µL (per well) cell lysis buffer containing 1× protease inhibitor cocktail (Target Mol). The nuclear and cytoplasmic proteins were isolated from cells using the Nuclear and Cytoplasmic Protein Extraction Kit (Beyotime, P0027) according to the manufacturer's instructions. The amount of loaded protein was normalized using a BCA Protein Quantification kit (Beyotime, P0012) and probed with a Western blot. The following antibodies were used: rabbit anti‐TDP‐43 polyclonal antibody (Proteintech, 10782‐2‐AP, 1: 2000), mouse anti‐HA tag monoclonal antibody (Proteintech, 66006‐2‐Ig, 1:5000), rabbit anti‐Histone‐H3 polyclonal antibody (Proteintech, 17168‐1‐AP, 1: 1000), mouse anti‐β‐actin monoclonal antibody (Proteintech, 66009‐1‐Ig, 1: 5000), HRP‐labeled goat anti‐rabbit IgG (H + L) (Beyotime, A0208, 1: 5000), and HRP‐labeled goat anti‐mouse IgG (H + L) (Beyotime, A0216, 1: 5000).

### 4.15 Immunofluorescence Staining

N2a and HEK‐293T cells were plated on coverslips in a 12‐well plate for one day and then transfected as indicated. The cells were washed three times with PBS, fixed with 4% (w/v) paraformaldehyde in PBS for 30 min, and then permeabilized with 0.25% Triton X‐100 in PBS at room temperature for 5 min. The cells were then blocked with 5% BSA in PBS at 37°C for 30 min and incubated with the appropriate dilution ratio of primary antibodies at 37°C for 2 h. After three washes with PBS for 5 min, fluorescence‐conjugated secondary antibodies at appropriate dilution ratios were applied to the samples and incubated in a dark room for 45 min. DAPI was then applied at the proper dilution; after being washed three times with PBS for 5 min and mounted with antifade mounting medium (Beyotime, P0126), the cells were subjected to a Leica TCS SP8 laser scanning confocal microscope (Wetzlar, Germany). The following antibodies were used: mouse anti‐HA tag monoclonal antibody (Proteintech, 66006‐2‐Ig, 1: 500), mouse anti‐P4HB monoclonal antibody (anti‐PDI antibody, Abcam, ab2792, 1: 500), rabbit anti‐G3BP1 polyclonal antibody (Proteintech, 13057‐2‐AP, 1: 500), rabbit anti‐pTDP‐43 (pSer409/410) polyclonal antibody (Cosmo, CAC‐TIP‐PTD‐P07, 1: 1000), Alexa Fluor 555‐labeled donkey anti‐mouse IgG (H + L) (Beyotime, A0460, 1: 1000), and Alexa Fluor 647‐labeled goat anti‐rabbit IgG (H + L) (Beyotime, A0468, 1: 1000).

### 4.16 Sarkosyl‐Insoluble Western Blotting

Sarkosyl‐insoluble Western blotting was used to investigate oxidative stress‐induced pathological aggregation of TDP‐43 in neuronal cells. N2a cells were transiently transfected with empty vector pcDNA3.1, pcDNA3.1‐HA‐PDI, pcDNA3.1‐HA‐dnPDI, or pcDNA3.1‐HA‐PDI Δ*b′* according to the manufacturer's instructions. After one‐day culture and 1‐h treatment with 1 mm H_2_O_2_ or PBS at 37°C, cells were harvested by centrifugation at 100 *g* at 4°C for 5 min and ruptured on ice for 20 min with cell lysis buffer containing 1× protease inhibitor cocktail (Target Mol). The cell lysates were then centrifuged at 17 000 *g* at 4°C for 20 min to remove cell debris. The amount of loaded protein was normalized using a BCA Protein Quantification kit (Beyotime). A part of the supernatant was incubated with 1% sarkosyl for 30 min at room temperature. The mixture was then ultracentrifuged at 150 000 *g* for 30 min, and the supernatant was carefully removed and washed once with PBS. The sarkosyl‐insoluble pellets were boiled in the SDS‐PAGE loading buffer for 10 min. The remaining of the supernatant, which served as the total protein sample, was also boiled in the SDS‐PAGE loading buffer for 10 min. The samples were separated by 12.5% SDS‐PAGE and then Western blotted. The sarkosyl‐insoluble pellets from the above cells were probed with the anti‐TDP‐43 antibody, and the corresponding cell lysates were probed using the anti‐TDP‐43 antibody, anti‐HA antibody, and anti‐β‐actin antibody, respectively, which served as the input controls. For calculating the amounts of sarkosyl‐insoluble TDP‐43, the ImageJ software (NIH) was used to assess the densitometry of TDP‐43 bands, including the insoluble full‐length TDP‐43 band and the band of insoluble C‐terminal TDP‐43 fragment of 35 kDa. The normalized amount of insoluble TDP‐43 aggregates in the above cells was determined as a ratio of the density of insoluble TDP‐43 aggregate bands over that of the total TDP‐43 bands in cell lysates. The following antibodies were used: rabbit anti‐TDP‐43 polyclonal antibody (Proteintech, 10782‐2‐AP, 1: 2000), mouse anti‐HA tag monoclonal antibody (Proteintech, 66006‐2‐Ig, 1:5000), mouse anti‐β‐actin monoclonal antibody (Proteintech, 66009‐1‐Ig, 1:5000), HRP‐labeled goat anti‐rabbit IgG (H + L) (Beyotime, A0208, 1:5000), and HRP‐labeled goat anti‐mouse IgG (H + L) (Beyotime, A0216, 1:5000).

### 4.17 Immunogold Electron Microscopy of TDP‐43 Aggregates

N2a cells were transiently transfected with empty vector pcDNA3.1, pcDNA3.1‐HA‐PDI, or pcDNA3.1‐HA‐dnPDI according to the manufacturer's instructions. After one‐day culture and 1‐h treatment with 1 mm H_2_O_2_ or PBS at 37°C, the above N2a cells were washed twice with cold PBS, digested with trypsin‐EDTA solution (Beyotime, C0201), resuspended with 1 mL PBS, and centrifuged at 100 *g* for 5 min at 4°C to harvest the cells. The cells were then resuspended with 450 µL of 10% sucrose solution in 10 mm Tris‐HCl buffer (pH 7.4) containing 1 mm EGTA, 0.8 m NaCl, and 1× protease inhibitor cocktail (Target Mol). The mixtures were sonicated 5 times on ice at 50 W and 5 s/5 s, and then centrifuged at 17 000 *g* for 20 min at 4°C to remove the cell debris. A part of the supernatant was incubated with 1% Sarkosyl for 30 min at room temperature. The mixture was then ultracentrifuged at 150 000 *g* for 30 min, and the supernatant was carefully removed and washed once with PBS. The sarkosyl‐insoluble pellets were resuspended in PBS (50 µL). Sample aliquots of 10 µL were absorbed onto nickel grids for 1 min, and then washed three times with water for 30 s. Samples on grids were incubated with rabbit anti‐TDP‐43 polyclonal antibody (Proteintech, 10782‐2‐AP, 1:1000) for 30 min at room temperature and then blocked with 0.1% BSA in PBS for 15 min. 10‐nm gold‐labeled homologous secondary antibody (goat anti‐rabbit IgG H&L, Abcam, ab27234, 1:20) was used to incubate the grids for 20 min at room temperature. Unbound gold‐labeled homologous secondary antibodies were removed by washing with 200 µL of water drop by drop. Samples on grids were then stained with 2% (w/v) uranyl acetate for 1 min. The stained samples were examined using a JEM‐1400 Plus transmission electron microscope (JEOL) operating at 100 kV.

### 4.18 Ultrathin Section TEM

N2a cells stably expressing TDP‐43 were transiently transfected with empty vector pcDNA3.1, pcDNA3.1‐HA‐PDI, or pcDNA3.1‐HA‐dnPDI. After one‐day culture and 1‐h treatment with 1 mm H_2_O_2_ or PBS at 37°C, cells were prefixed with 3% paraformaldehyde and 1.5% glutaraldehyde in PBS, then harvested and postfixed in 1% osmium tetroxide for 1 h using an ice bath; the samples were then dehydrated in graded acetone and embedded in 812 resins. Ultrathin sections of the cells were prepared using a Leica Ultracut S Microtome and negatively stained using 2% uranyl acetate and lead citrate. The doubly stained ultrathin sections of cells were examined using a JEM‐1400 Plus transmission electron microscope (JEOL) operating at 100 kV. All experiments were further confirmed by biological repeats.

### 4.19 RNA Extraction, cDNA Synthesis, and RT‐qPCR

Total RNA was extracted from HEK‐293T cells with Trizol (Beyotime, R0016) according to the manufacturer's instructions. RNA concentrations were measured by NanoDrop OneC Microvolume UV–vis Spectrophotometer (Thermo Fisher Scientific), and 1000 ng of RNA was used for reverse transcription. cDNA was synthesized by ABScript Neo RT Master Mix for qPCR with gDNA Remover (Abclone, RK20433) following the manufacturer's protocol. qPCR was run with cDNA input in a 10 µL reaction using 2× Universal SYBR Green Fast qPCR Mix (Abclone, RK21203) with readout on a BIO‐RAD CFX connect using standard cycling parameters (95°C for 3 min, 40 cycles of 95°C for 5 s and 60°C for 30 s), followed by standard dissociation (ramp from 65°C to 95°C at 0.1°C/s). ΔΔ*Ct* was calculated with the housekeeper gene *ACTB* as control and (PDI + H_2_O_2_) groups as reference. The primer sequences are listed in Table S1.

### 4.20 Statistical Analysis

The data shown for each experiment were based on at least three technical replicates, as indicated in individual figure legends. Data are presented as the mean ± SD, and *p*‐values were determined using two‐sided Student's *t*‐tests. Differences were considered statistically significant when *p* < 0.05. Statistical analyses were performed using one‐way ANOVA followed by Bonferroni multiple comparison tests, and different letters indicate significant differences at the level of *p* < 0.05. All experiments were further confirmed by biological repeats.

## Author Contributions

Jia‐Qi Liu: data curation, formal analysis, investigation, methodology, software, validation, visualization, and writing the original draft preparation. Hao Liu: data curation, investigation, methodology, validation, and visualization. Yu‐Xuan Sun: investigation, methodology, validation, and visualization. Yuying Li: investigation, methodology, and validation. Xiangyi Liu: methodology and resources. Li‐Qiang Wang: funding acquisition, investigation, and methodology. Zhaofei Yang: methodology and resources. Qi Fu: methodology and resources. Xiaojiao Xu: methodology and resources. Jie Chen: project administration, resources, and software. Yingshuang Zhang: methodology and resources. Jun Zhou: Methodology and Resources. Weidong Le: funding acquisition, resources, and writing the original draft preparation. Mengchao Cui: supervision, funding acquisition, methodology, resources, validation, and writing the original draft preparation. Yi Liang: conceptualization, data curation, formal analysis, funding acquisition, investigation, methodology, project administration, supervision, validation, visualization, writing the original draft preparation, and writing the reviewing and editing.

## Funding

This work was supported by funding from the National Natural Science Foundation of China Grant 32271326 to Y. L., the National Key Research and Development Program of China Grant 2024YFA1307300 to Y. L., and the National Natural Science Foundation of China Grant 32071212 to Y. L. L.‐Q.W. acknowledges financial support from the Noncommunicable Chronic Diseases‐National Science and Technology Major Project Grant 2023ZD0507202. M.C. was supported by funding from the National Natural Science Foundation of China Grants U1967221 and 22022601. W.L. and J.Z. were supported by funding from the National Natural Science Foundation of China Grants 82271524 and 32371360, respectively.

## Conflicts of Interest

The authors declare no conflicts of interest.

## Supporting information




**Supporting File 1**: advs75718‐sup‐0001‐SuppMat.doc.

**Supporting File 2**: advs75718‐sup‐0002‐Data.zip.

## Data Availability

All data generated or analyzed during this study are included in this article or available from the corresponding author upon request.

## References

[1] T. J. Cohen , V. M. Y. Lee , and J. Q. Trojanowski , “TDP‐43 Functions and Pathogenic Mechanisms Implicated in TDP‐43 Proteinopathies,” Trends in Molecular Medicine 17, no. 11 (2011): 659–667, 10.1016/j.molmed.2011.06.004. 21783422 PMC3202652

[2] M. Neumann , D. M. Sampathu , L. K. Kwong , et al., “Ubiquitinated TDP‐43 in Frontotemporal Lobar Degeneration and Amyotrophic Lateral Sclerosis,” Science 314, no. 5796 (2006): 130–133, 10.1126/science.1134108. 17023659

[3] X. Wang , Y. Hu , and R. Xu , “The Pathogenic Mechanism of TAR DNA‐Binding Protein 43 (TDP‐43) in Amyotrophic Lateral Sclerosis,” Neural Regeneration Research 19, no. 4 (2024): 800–806, 10.4103/1673-5374.382233. 37843214 PMC10664110

[4] Y. Iguchi , Y. Takahashi , J. Li , et al., “IκB Kinase Phosphorylates Cytoplasmic TDP‐43 and Promotes Its Proteasome Degradation,” Journal of Cell Biology 223, no. 2 (2024): 202302048, 10.1083/jcb.202302048. PMC10783433 38197897

[5] A. E. Perlegos , J. Durkin , S. J. Belfer , et al., “TDP‐43 Impairs Sleep in Drosophila Through Ataxin‐2 –Dependent Metabolic Disturbance,” Science Advances 10, no. 2 (2024): adj4457, 10.1126/sciadv.adj4457. PMC10780954 38198547

[6] K. Sharma , F. Stockert , J. Shenoy , et al., “Cryo‐EM Observation of the Amyloid Key Structure of Polymorphic TDP‐43 Amyloid Fibrils,” Nature Communications 15, no. 1 (2024): 486, 10.1038/s41467-023-44489-0. PMC10784485 38212334

[7] J. I. Ayers and D. R. Borchelt , “Phenotypic Diversity in ALS and the Role of Poly‐Conformational Protein Misfolding,” Acta Neuropathologica 142, no. 1 (2021): 41–55, 10.1007/s00401-020-02222-x. 32930869 PMC7956917

[8] W.‐C. Xu , J.‐Z. Liang , C. Li , et al., “Pathological Hydrogen Peroxide Triggers the Fibrillization of Wild‐Type SOD1 via Sulfenic Acid Modification of Cys‐111,” Cell Death & Disease 9, no. 2 (2018): 67, 10.1038/s41419-017-0106-4. 29358575 PMC5833406

[9] L.‐Q. Wang , Y. Ma , H.‐Y. Yuan , et al., “Cryo‐EM Structure of an Amyloid Fibril Formed by Full‐Length Human SOD1 Reveals Its Conformational Conversion,” Nature Communications 13, no. 1 (2022): 3491, 10.1038/s41467-022-31240-4. PMC9205981 35715417

[10] P. Foulds , E. McAuley , L. Gibbons , et al., “TDP‐43 Protein in Plasma May Index TDP‐43 Brain Pathology in Alzheimer's Disease and Frontotemporal Lobar Degeneration,” Acta Neuropathologica 116, no. 2 (2008): 141–146, 10.1007/s00401-008-0389-8. 18506455 PMC2464623

[11] W. T. Hu , K. A. Josephs , D. S. Knopman , et al., “Temporal Lobar Predominance of TDP‐43 Neuronal Cytoplasmic Inclusions in Alzheimer Disease,” Acta Neuropathologica 116, no. 2 (2008): 215–220, 10.1007/s00401-008-0400-4. 18592255 PMC3404722

[12] K. A. Josephs , M. E. Murray , J. L. Whitwell , et al., “Staging TDP‐43 Pathology in Alzheimer's Disease,” Acta Neuropathologica 127, no. 3 (2014): 441–450, 10.1007/s00401-013-1211-9. 24240737 PMC3944799

[13] B. D. James , R. S. Wilson , P. A. Boyle , J. Q. Trojanowski , D. A. Bennett , and J. A. Schneider , “TDP‐43 Stage, Mixed Pathologies, and Clinical Alzheimer's‐Type Dementia,” Brain 139, no. 11 (2016): 2983–2993, 10.1093/brain/aww224. 27694152 PMC5091047

[14] Y.‐H. Shih , L.‐H. Tu , T.‐Y. Chang , et al., “TDP‐43 Interacts With Amyloid‐β, Inhibits Fibrillization, and Worsens Pathology in a Model of Alzheimer's Disease,” Nature Communications 11, no. 1 (2020): 5950, 10.1038/s41467-020-19786-7. PMC7683652 33230138

[15] A. Meneses , S. Koga , J. O'Leary , D. W. Dickson , G. Bu , and N. Zhao , “TDP‐43 Pathology in Alzheimer's Disease,” Molecular Neurodegeneration 16, no. 1 (2021): 84, 10.1186/s13024-021-00503-x. 34930382 PMC8691026

[16] A. F. Carlos , N. Tosakulwong , S. D. Weigand , et al., “TDP‐43 Pathology Effect on Volume and Flortaucipir Uptake in Alzheimer's Disease,” Alzheimer's & Dementia 19, no. 6 (2022): 2343–2354, 10.1002/alz.12878. PMC10239529 36463537

[17] V. Estades Ayuso , S. Pickles , T. Todd , et al., “TDP‐43‐Regulated Cryptic RNAs Accumulate in Alzheimer's Disease Brains,” Molecular Neurodegeneration 18, no. 1 (2023): 57, 10.1186/s13024-023-00646-z. 37605276 PMC10441763

[18] A. R. Agra Almeida Quadros , Z. Li , X. Wang , et al., “Cryptic Splicing of Stathmin‐2 and UNC13A mRNAs Is a Pathological Hallmark of TDP‐43‐Associated Alzheimer's Disease,” Acta Neuropathologica 147, no. 1 (2024): 9, 10.1007/s00401-023-02655-0. 38175301 PMC10766724

[19] P. Kosuri , J. Alegre‐Cebollada , J. Feng , et al., “Protein Folding Drives Disulfide Formation,” Cell 151, no. 4 (2012): 794–806, 10.1016/j.cell.2012.09.036. 23141538 PMC3506382

[20] C. Wang , W. Li , J. Ren , et al., “Structural Insights Into the Redox‐Regulated Dynamic Conformations of Human Protein Disulfide Isomerase,” Antioxidants & Redox Signaling 19, no. 1 (2013): 36–45, 10.1089/ars.2012.4630. 22657537

[21] T. Uehara , T. Nakamura , D. Yao , et al., “S‐Nitrosylated Protein‐Disulphide Isomerase Links Protein Misfolding to Neurodegeneration,” Nature 441, no. 7092 (2006): 513–517, 10.1038/nature04782. 16724068

[22] A. K. Walker , M. A. Farg , C. R. Bye , C. A. McLean , M. K. Horne , and J. D. Atkin , “Protein Disulphide Isomerase Protects Against Protein Aggregation and Is S‐Nitrosylated in Amyotrophic Lateral Sclerosis,” Brain 133, no. Pt 1 (2010): 105–116, 10.1093/brain/awp267. 19903735

[23] S. Parakh , S. Shadfar , E. R. Perri , et al., “The Redox Activity of Protein Disulfide Isomerase Inhibits ALS Phenotypes in Cellular and Zebrafish Models,” Iscience 23, no. 5 (2020): 101097, 10.1016/j.isci.2020.101097. 32446203 PMC7240177

[24] Y. Honjo , S. Kaneko , H. Ito , et al., “Protein Disulfide Isomerase‐Immunopositive Inclusions in Patients With Amyotrophic Lateral Sclerosis,” Amyotrophic Lateral Sclerosis 12, no. 6 (2011): 444–450, 10.3109/17482968.2011.594055. 21745122

[25] D. B. Medinas , P. Rozas , and C. Hetz , “Critical Roles of Protein Disulfide Isomerases in Balancing Proteostasis in the Nervous System,” Journal of Biological Chemistry 298, no. 7 (2022): 102087, 10.1016/j.jbc.2022.102087. 35654139 PMC9253707

[26] Y. Tabata , K. Takano , T. Ito , et al., “Vaticanol B, a Resveratrol Tetramer, Regulates Endoplasmic Reticulum Stress and Inflammation,” American Journal of Physiology‐Cell Physiology 293, no. 1 (2007): C411–C418, 10.1152/ajpcell.00095.2007. 17475668

[27] C. Turano , S. Coppari , F. Altieri , and A. Ferraro , “Proteins of the PDI family: Unpredicted Non‐ER Locations and Functions,” Journal of Cellular Physiology 193 (2002): 154–163, 10.1002/jcp.10172. 12384992

[28] A. Igbaria , P. I. Merksamer , A. Trusina , et al., “Chaperone‐Mediated Reflux of Secretory Proteins to the Cytosol During Endoplasmic Reticulum Stress,” Proceedings of the National Academy of Sciences 116, no. 23 (2019): 11291–11298, 10.1073/pnas.1904516116. PMC6561268 31101715

[29] D. Sicari , F. G. Centonze , R. Pineau , et al., “Reflux of Endoplasmic Reticulum Proteins to the Cytosol Inactivates Tumor Suppressors,” EMBO Reports 22, no. 5 (2021): 51412, 10.15252/embr.202051412. PMC8724677 33710763

[30] A. D. D. Purificação , V. Debbas , L. Y. Tanaka , et al., “DNAJB12 and DNJB14 Are Non‐Redundant Hsp40 Redox Chaperones Involved in Endoplasmic Reticulum Protein Reflux,” Biochimica et Biophysica Acta (BBA)—General Subjects 1868, no. 1 (2024): 130502, 10.1016/j.bbagen.2023.130502. 37925033

[31] S. Parakh , E. R. Perri , M. Vidal , et al., “Protein Disulfide Isomerase Endoplasmic Reticulum Protein 57 (ERp57) Is Protective Against ALS‐Associated Mutant TDP‐43 in Neuronal Cells,” NeuroMolecular Medicine 26, no. 1 (2024): 23, 10.1007/s12017-024-08787-0. 38861223 PMC11166824

[32] L. P. Bergeron‐Sandoval , N. Safaee , and S. W. Michnick , “Mechanisms and Consequences of Macromolecular Phase Separation,” Cell 165, no. 5 (2016): 1067–1079, 10.1016/j.cell.2016.05.026. 27203111

[33] S. Boeynaems , S. Alberti , N. L. Fawzi , et al., “Protein Phase Separation: A New Phase in Cell Biology,” Trends in Cell Biology 28, no. 6 (2018): 420–435, 10.1016/j.tcb.2018.02.004. 29602697 PMC6034118

[34] S. F. Shimobayashi , P. Ronceray , D. W. Sanders , M. P. Haataja , and C. P. Brangwynne , “Nucleation Landscape of Biomolecular Condensates,” Nature 599, no. 7885 (2021): 503–506, 10.1038/s41586-021-03905-5. 34552246

[35] Y. Shin and C. P. Brangwynne , “Liquid Phase Condensation in Cell Physiology and Disease,” Science 357, no. 6357 (2017): aaf4382, 10.1126/science.aaf4382. 28935776

[36] S. Maharana , J. Wang , D. K. Papadopoulos , et al., “RNA Buffers the Phase Separation Behavior of Prion‐Like RNA Binding Proteins,” Science 360, no. 6391 (2018): 918–921, 10.1126/science.aar7366. 29650702 PMC6091854

[37] F. Gasset‐Rosa , S. Lu , H. Yu , et al., “Cytoplasmic TDP‐43 De‐Mixing Independent of Stress Granules Drives Inhibition of Nuclear Import, Loss of Nuclear TDP‐43, and Cell Death,” Neuron 102, no. 2 (2019): 339–357.e7, 10.1016/j.neuron.2019.02.038. 30853299 PMC6548321

[38] W. M. Babinchak , B. K. Dumm , S. Venus , et al., “Small Molecules as Potent Biphasic Modulators of Protein Liquid‐Liquid Phase Separation,” Nature Communications 11, no. 1 (2020): 5574, 10.1038/s41467-020-19211-z. PMC7643064 33149109

[39] H. Yu , S. Lu , K. Gasior , et al., “HSP70 Chaperones RNA‐Free TDP‐43 Into Anisotropic Intranuclear Liquid Spherical Shells,” Science 371, no. 6529 (2021): abb4309, 10.1126/science.abb4309. PMC8286096 33335017

[40] L. A. Gruijs da Silva , F. Simonetti , S. Hutten , et al., “Disease‐Linked TDP‐43 Hyperphosphorylation Suppresses TDP‐43 Condensation and Aggregation,” EMBO Journal 41, no. 8 (2022): 108443, 10.15252/embj.2021108443. PMC9016352 35112738

[41] S. Lu , J. Hu , O. A. Arogundade , et al., “Heat‐Shock Chaperone HSPB1 Regulates Cytoplasmic TDP‐43 Phase Separation and Liquid‐to‐Gel Transition,” Nature Cell Biology 24, no. 9 (2022): 1378–1393, 10.1038/s41556-022-00988-8. 36075972 PMC9872726

[42] J. Tao , Y. Zeng , B. Dai , et al., “Excess PrPC Inhibits Muscle Cell Differentiation via miRNA‐Enhanced Liquid–Liquid Phase Separation Implicated in Myopathy,” Nature Communications 14, no. 1 (2023): 8131, 10.1038/s41467-023-43826-7. PMC10709375 38065962

[43] X. Zhou , L. Sumrow , K. Tashiro , et al., “Mutations Linked to Neurological Disease Enhance Self‐Association of Low‐Complexity Protein Sequences,” Science 377, no. 6601 (2022): abn5582, 10.1126/science.abn5582. PMC9610444 35771920

[44] J. R. Mann , A. M. Gleixner , J. C. Mauna , et al., “RNA Binding Antagonizes Neurotoxic Phase Transitions of TDP‐43,” Neuron 102, no. 2 (2019): 321–338.e8, 10.1016/j.neuron.2019.01.048. 30826182 PMC6472983

[45] J. Garcia Morato , F. Hans , F. von Zweydorf , et al., “Sirtuin‐1 Sensitive Lysine‐136 Acetylation Drives Phase Separation and Pathological Aggregation of TDP‐43,” Nature Communications 13, no. 1 (2022): 1223, 10.1038/s41467-022-28822-7. PMC8907366 35264561

[46] A. E. Conicella , G. L. Dignon , G. H. Zerze , et al., “TDP‐43 α‐Helical Structure Tunes Liquid–Liquid Phase Separation and Function,” Proceedings of the National Academy of Sciences 117, no. 11 (2020): 5883–5894, 10.1073/pnas.1912055117. PMC7084079 32132204

[47] A. Wang , A. E. Conicella , H. B. Schmidt , et al., “A Single N‐Terminal Phosphomimic Disrupts TDP‐43 Polymerization, Phase Separation, and RNA Splicing,” EMBO Journal 37, no. 5 (2018): 97452, 10.15252/embj.201797452. PMC5830921 29438978

[48] X. Yan , D. Kuster , P. Mohanty , et al., “Intra‐Condensate Demixing of TDP‐43 Inside Stress Granules Generates Pathological Aggregates,” Cell 188, no. 15 (2025): 4123–4140.e18, 10.1016/j.cell.2025.04.039. 40412392 PMC12303766

[49] J. Zhang , J. Hu , R. Liu , et al., “YAP Maintains the Dynamics of TDP‐43 Condensates and Antagonizes TDP‐43 Pathological Aggregates,” Nature Cell Biology 27, no. 7 (2025): 1148–1160, 10.1038/s41556-025-01685-y. 40542195

[50] X. Zuo , J. Zhou , Y. Li , et al., “TDP‐43 Aggregation Induced by Oxidative Stress Causes Global Mitochondrial Imbalance in ALS,” Nature Structural & Molecular Biology 28, no. 2 (2021): 132–142, 10.1038/s41594-020-00537-7. 33398173

[51] Y. Iguchi , M. Katsuno , S. Takagi , et al., “Oxidative Stress Induced by Glutathione Depletion Reproduces Pathological Modifications of TDP‐43 Linked to TDP‐43 Proteinopathies,” Neurobiology of Disease 45, no. 3 (2012): 862–870, 10.1016/j.nbd.2011.12.002. 22198567

[52] L. Kang , M. Piao , N. Liu , W. Gu , and C. Feng , “Sevoflurane Exposure Induces Neuronal Cell Ferroptosis Initiated by Increase of Intracellular Hydrogen Peroxide in the Developing Brain via ER Stress ATF3 Activation,” Molecular Neurobiology 61, no. 4 (2024): 2313–2335, 10.1007/s12035-023-03695-z. 37874483 PMC10972952

[53] C. D. Hu , Y. Chinenov , and T. K. Kerppola , “Visualization of Interactions Among bZIP and Rel Family Proteins in Living Cells Using Bimolecular Fluorescence Complementation,” Molecular Cell 9, no. 4 (2002): 789–798, 10.1016/s1097-2765(02)00496-3. 11983170

[54] C. D. Hu and T. K. Kerppola , “Simultaneous Visualization of Multiple Protein Interactions in Living Cells Using Multicolor Fluorescence Complementation Analysis,” Nature Biotechnology 21, no. 5 (2003): 539–545, 10.1038/nbt816. PMC1820765 12692560

[55] P. Klappa , L. W. Ruddock , N. J. Darby , and R. B. Freedman , “The b' Domain Provides the Principal Peptide‐Binding Site of Protein Disulfide Isomerase but All Domains Contribute to Binding of Misfolded Proteins,” EMBO Journal 17, no. 4 (1998): 927–935, 10.1093/emboj/17.4.927. 9463371 PMC1170442

[56] V. D. Nguyen , K. Wallis , M. J. Howard , et al., “Alternative Conformations of the X Region of Human Protein Disulphide‐Isomerase Modulate Exposure of the Substrate Binding b′ Domain,” Journal of Molecular Biology 383, no. 5 (2008): 1144–1155, 10.1016/j.jmb.2008.08.085. 18801374

[57] S. Alberti , A. Gladfelter , and T. Mittag , “Considerations and Challenges in Studying Liquid‐Liquid Phase Separation and Biomolecular Condensates,” Cell 176, no. 3 (2019): 419–434, 10.1016/j.cell.2018.12.035. 30682370 PMC6445271

[58] B. Dai , T. Zhong , Z.‐X. Chen , et al., “Myricetin Slows Liquid–Liquid Phase Separation of Tau and Activates ATG5‐Dependent Autophagy to Suppress Tau Toxicity,” Journal of Biological Chemistry 297, no. 4 (2021): 101222, 10.1016/j.jbc.2021.101222. 34560101 PMC8551527

[59] Y.‐Y. Gao , T. Zhong , L.‐Q. Wang , et al., “Zinc Enhances Liquid‐Liquid Phase Separation of Tau Protein and Aggravates Mitochondrial Damages in Cells,” International Journal of Biological Macromolecules 209, no. Pt A (2022): 703–715, 10.1016/j.ijbiomac.2022.04.034. 35405154

[60] X.‐N. Li , Y. Gao , Y. Li , et al., “Arg177 and Asp159 From Dog Prion Protein Slow Liquid–Liquid Phase Separation and Inhibit Amyloid Formation of Human Prion Protein,” Journal of Biological Chemistry 299, no. 11 (2023): 105329, 10.1016/j.jbc.2023.105329. 37805139 PMC10641668

[61] P. Yang , C. Mathieu , R.‐M. Kolaitis , et al., “G3BP1 is a Tunable Switch That Triggers Phase Separation to Assemble Stress Granules,” Cell 181, no. 2 (2020): 325–345.e28, 10.1016/j.cell.2020.03.046. 32302571 PMC7448383

[62] Y. Gwon , B. A. Maxwell , R. M. Kolaitis , P. Zhang , H. J. Kim , and J. P. Taylor , “Ubiquitination of G3BP1 Mediates Stress Granule Disassembly in a Context‐Specific Manner,” Science 372, no. 6549 (2021): abf6548, 10.1126/science.abf6548. PMC8574224 34739333

[63] C. Yang , Z. Wang , Y. Kang , et al., “Stress Granule Homeostasis Is Modulated by TRIM21‐Mediated Ubiquitination of G3BP1 and Autophagy‐Dependent Elimination of Stress Granules,” Autophagy 19, no. 7 (2023): 1934–1951, 10.1080/15548627.2022.2164427. 36692217 PMC10283440

[64] B. D. Freibaum , J. Messing , H. Nakamura , et al., “Identification of Small Molecule Inhibitors of G3BP‐Driven Stress Granule Formation,” Journal of Cell Biology 223, no. 3 (2024): 202308083, 10.1083/jcb.202308083. PMC10824102 38284934

[65] I. I. Sanchez , T. B. Nguyen , W. E. England , et al., “Huntington's Disease Mice and Human Brain Tissue Exhibit Increased G3BP1 Granules and TDP43 Mislocalization,” Journal of Clinical Investigation 131, no. 12 (2021): 140723, 10.1172/JCI140723. 33945510 PMC8203471

[66] S. C. Barber and P. J. Shaw , “Oxidative Stress in ALS: Key Role in Motor Neuron Injury and Therapeutic Target,” Free Radical Biology and Medicine 48, no. 5 (2010): 629–641, 10.1016/j.freeradbiomed.2009.11.018. 19969067

[67] E. V. Ilieva , V. Ayala , M. Jove , et al., “Oxidative and Endoplasmic Reticulum Stress Interplay in Sporadic Amyotrophic Lateral Sclerosis,” Brain 130, no. Pt 12 (2007): 3111–3123, 10.1093/brain/awm190. 17716997

[68] N. F. Liachko , P. J. McMillan , T. J. Strovas , et al., “The Tau Tubulin Kinases TTBK1/2 Promote Accumulation of Pathological TDP‐43,” PLoS Genetics 10, no. 12 (2014): 1004803, 10.1371/journal.pgen.1004803. PMC4256087 25473830

[69] X. R. Ma , M. Prudencio , Y. Koike , et al., “TDP‐43 Represses Cryptic Exon Inclusion in the FTD–ALS Gene UNC13A,” Nature 603, no. 7899 (2022): 124–130, 10.1038/s41586-022-04424-7. 35197626 PMC8891019

[70] A.‐L. Brown , O. G. Wilkins , M. J. Keuss , et al., “TDP‐43 Loss and ALS‐Risk SNPs Drive Mis‐Splicing and Depletion of UNC13A,” Nature 603, no. 7899 (2022): 131–137, 10.1038/s41586-022-04436-3. 35197628 PMC8891020

[71] M. Jo , S. Lee , Y. M. Jeon , S. Kim , Y. Kwon , and H. J. Kim , “The Role of TDP‐43 Propagation in Neurodegenerative Diseases: Integrating Insights From Clinical and Experimental Studies,” Experimental & Molecular Medicine 52, no. 10 (2020): 1652–1662, 10.1038/s12276-020-00513-7. 33051572 PMC8080625

[72] B. A. Keller , K. Volkening , C. A. Droppelmann , L. C. Ang , R. Rademakers , and M. J. Strong , “Co‐Aggregation of RNA Binding Proteins in ALS Spinal Motor Neurons: Evidence of a Common Pathogenic Mechanism,” Acta Neuropathologica 124, no. 5 (2012): 733–747, 10.1007/s00401-012-1035-z. 22941224

[73] L. T.‐W. Lin , A. Razzaq , S. E. Di Gregorio , et al., “Hsp90 and Its co‐Chaperone Sti1 Control TDP‐43 Misfolding and Toxicity,” FASEB Journal 35, no. 5 (2021): 21594, 10.1096/fj.202002645R. 33908654

[74] K. Luo , Z. Wang , K. Zhuang , S. Yuan , F. Liu , and A. Liu , “Suberoylanilide Hydroxamic Acid Suppresses Axonal Damage and Neurological Dysfunction After Subarachnoid Hemorrhage via the HDAC1/HSP70/TDP‐43 Axis,” Experimental & Molecular Medicine 54, no. 9 (2022): 1423–1433, 10.1038/s12276-022-00761-9. 35501375 PMC9535006

[75] E. Feneberg , D. Gordon , A. G. Thompson , et al., “An ALS‐Linked Mutation in TDP‐43 Disrupts Normal Protein Interactions in the Motor Neuron Response to Oxidative Stress,” Neurobiology of Disease 144 (2020): 105050, 10.1016/j.nbd.2020.105050. 32800996

[76] M. Vendruscolo and M. Fuxreiter , “Protein Condensation Diseases: Therapeutic Opportunities,” Nature Communications 13, no. 1 (2022): 5550, 10.1038/s41467-022-32940-7. PMC9500012 36138006

[77] A. Patil , A. R. Strom , J. A. Paulo , et al., “A Disordered Region Controls cBAF Activity via Condensation and Partner Recruitment,” Cell 186, no. 22 (2023): 4936–4955.e26, 10.1016/j.cell.2023.08.032. 37788668 PMC10792396

[78] M. V. Vega , A. Nigro , S. Luti , et al., “Isolation and Characterization of Soluble Human Full‐Length TDP‐43 Associated With Neurodegeneration,” FASEB Journal 33, no. 10 (2019): 10780–10793, 10.1096/fj.201900474R. 31287959

[79] K. Wang , J.‐Q. Liu , T. Zhong , et al., “Phase Separation and Cytotoxicity of Tau Are Modulated by Protein Disulfide Isomerase and S‐Nitrosylation of This Molecular Chaperone,” Journal of Molecular Biology 432, no. 7 (2020): 2141–2163, 10.1016/j.jmb.2020.02.013. 32087196

