# Supplementary figures and images for: Protein Disulfide Isomerase Disassembles TDP‐43/G3BP1 Condensates and Antagonizes TDP‐43 Pathological Aggregates
Source: Adv Sci (Weinh). 2026 May 25;13(38):e16846. doi: 10.1002/advs.202516846 (PMC13336076; doi:10.1002/advs.202516846)

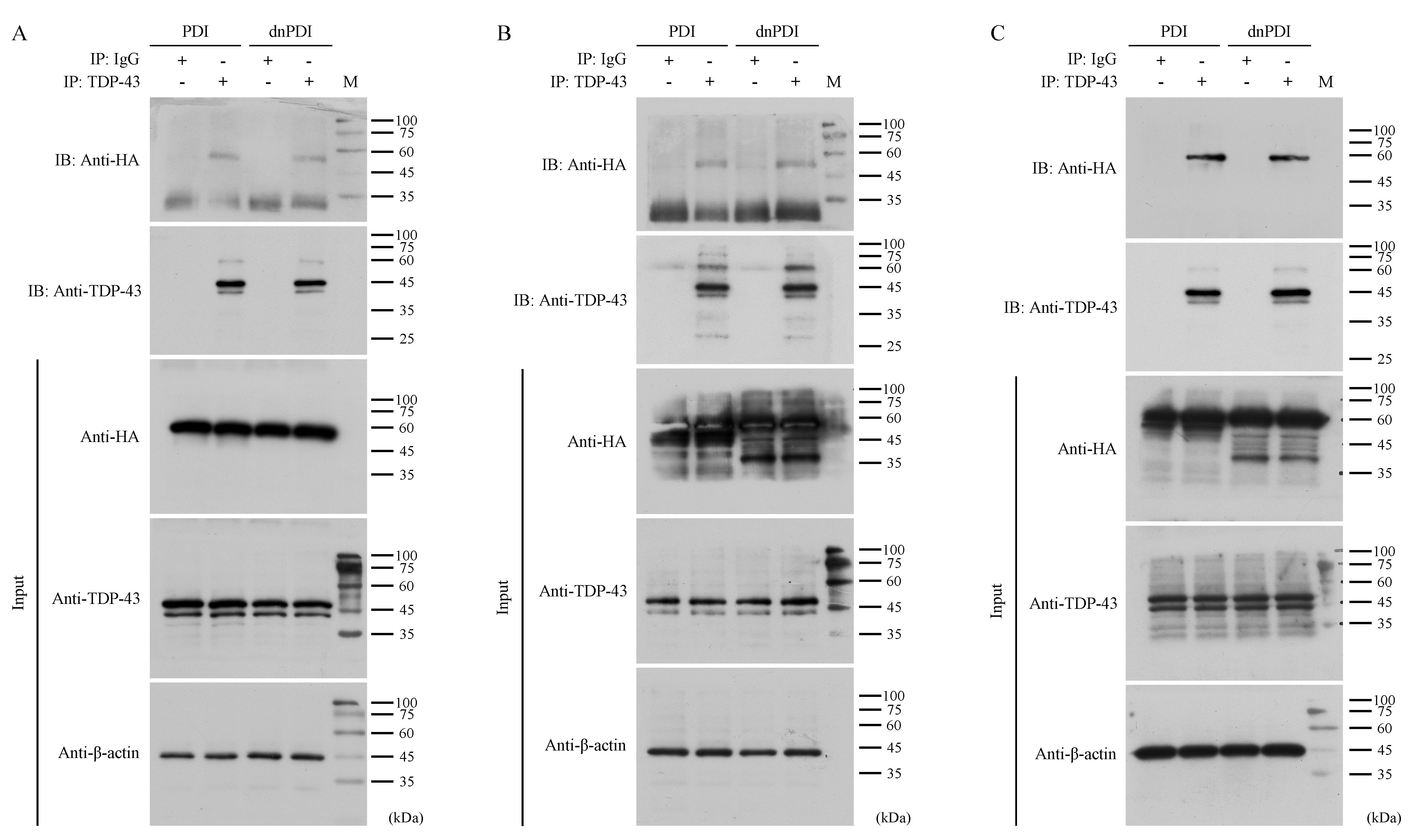

Supplement: Supplementary file 2 — Supporting File 2: advs75718‐sup‐0002‐Data.zip. [file ADVS-13-e16846-s001.zip › advs75718-sup-0002-Data/Fig1A_Uncropped_images.tif]

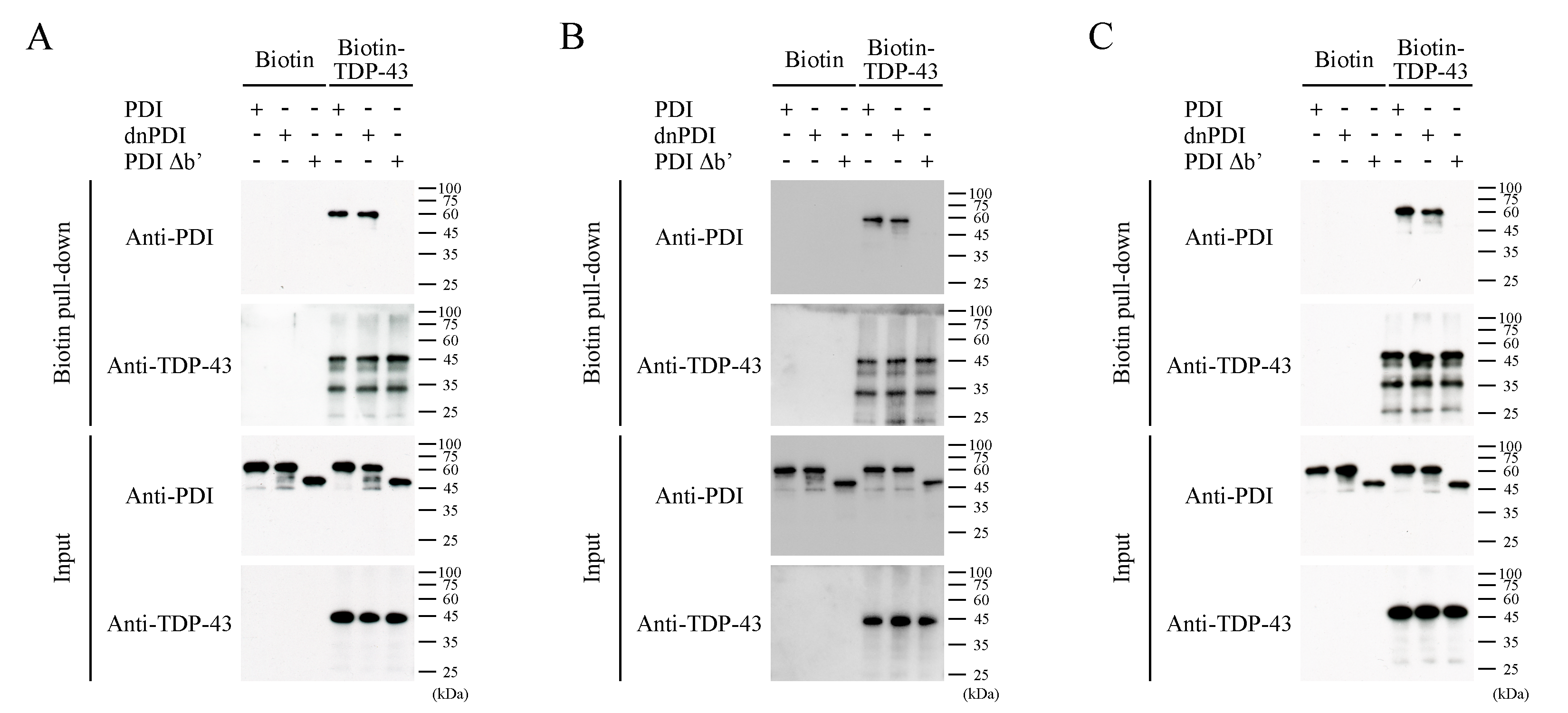

Supplement: Supplementary file 2 — Supporting File 2: advs75718‐sup‐0002‐Data.zip. [file ADVS-13-e16846-s001.zip › advs75718-sup-0002-Data/Fig1G_Uncropped_images.tif]

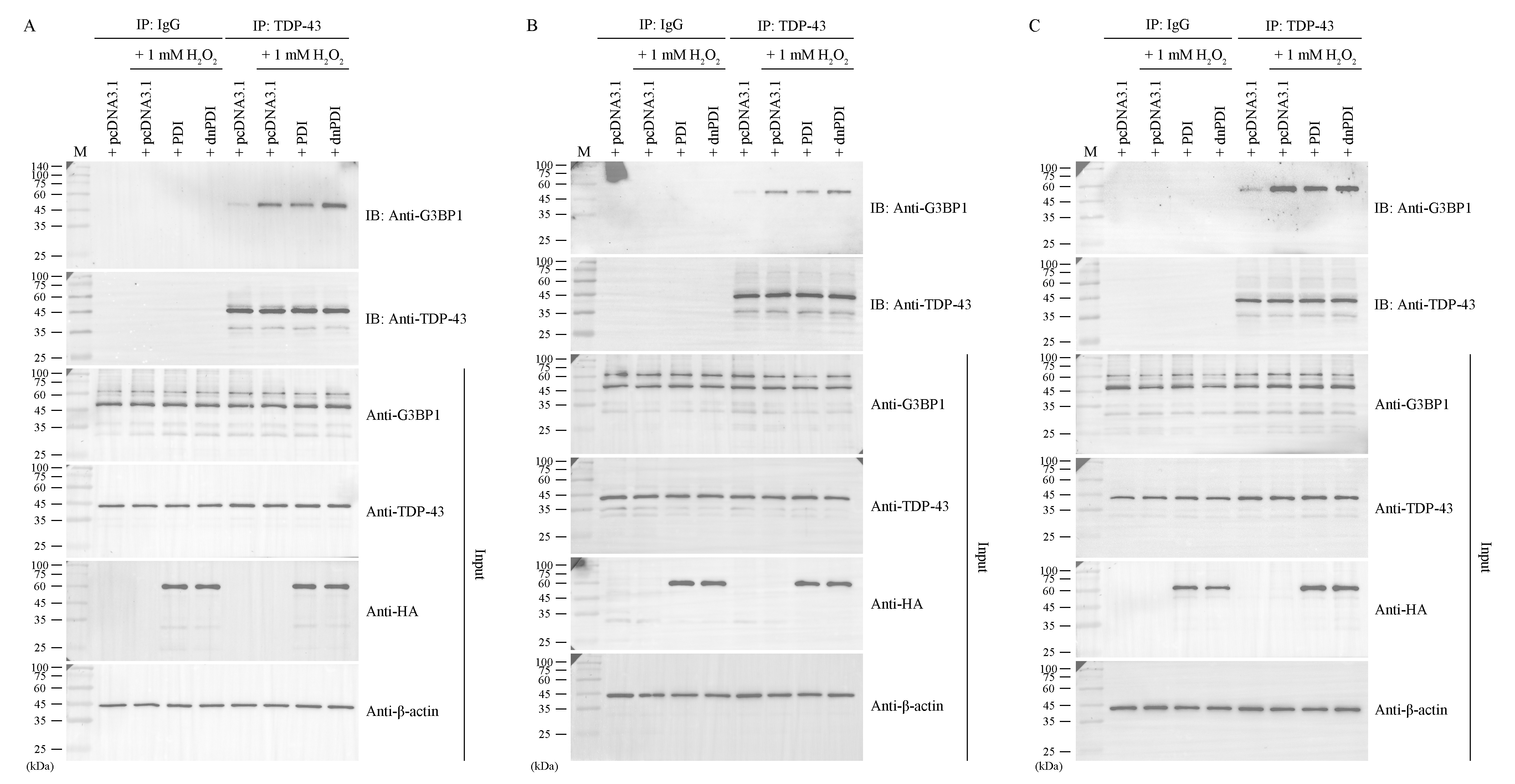

Supplement: Supplementary file 2 — Supporting File 2: advs75718‐sup‐0002‐Data.zip. [file ADVS-13-e16846-s001.zip › advs75718-sup-0002-Data/Fig4A_Uncropped_images.tif]

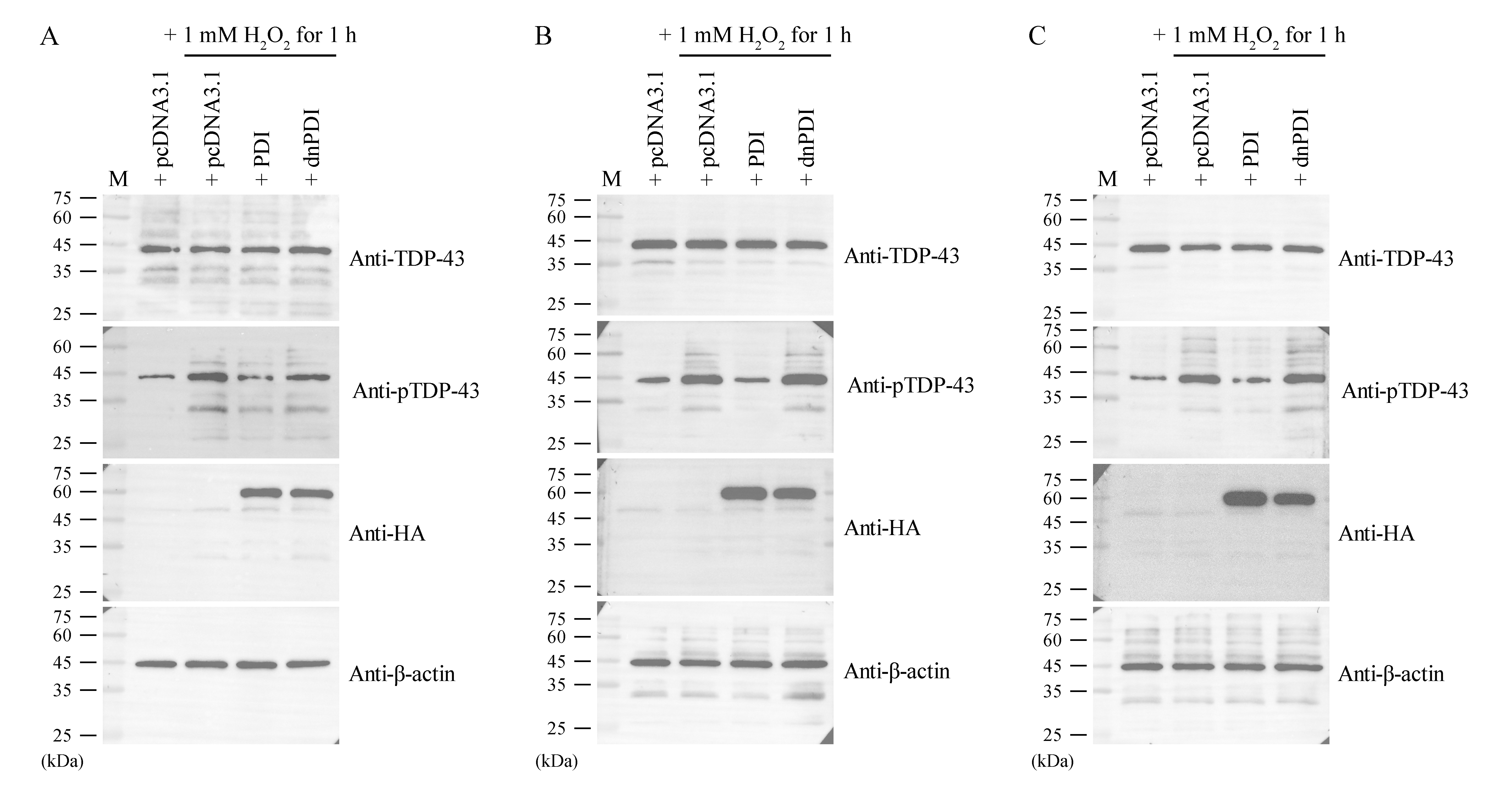

Supplement: Supplementary file 2 — Supporting File 2: advs75718‐sup‐0002‐Data.zip. [file ADVS-13-e16846-s001.zip › advs75718-sup-0002-Data/Fig5A_Uncropped_images.tif]

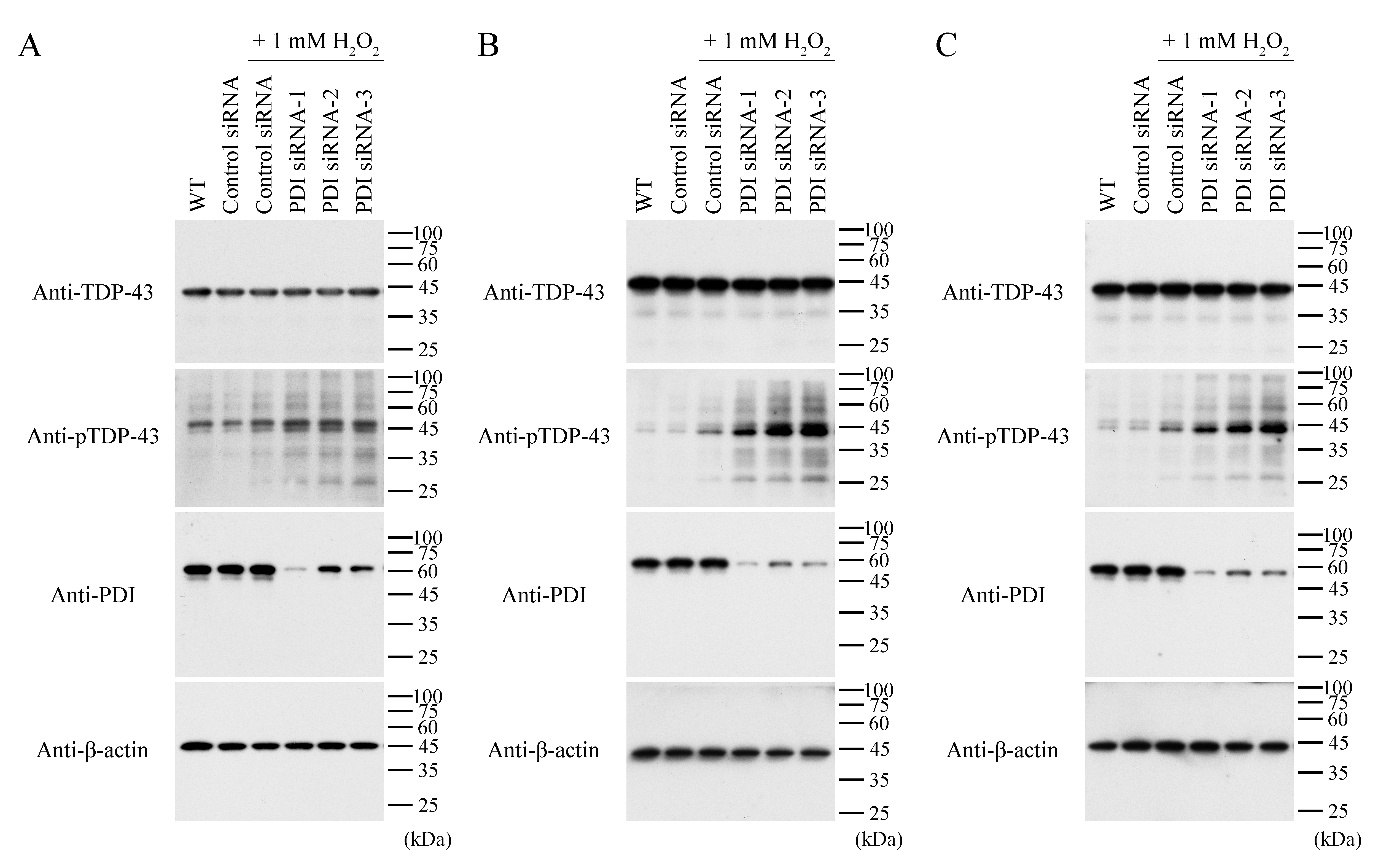

Supplement: Supplementary file 2 — Supporting File 2: advs75718‐sup‐0002‐Data.zip. [file ADVS-13-e16846-s001.zip › advs75718-sup-0002-Data/Fig5D_Uncropped_images.tif]

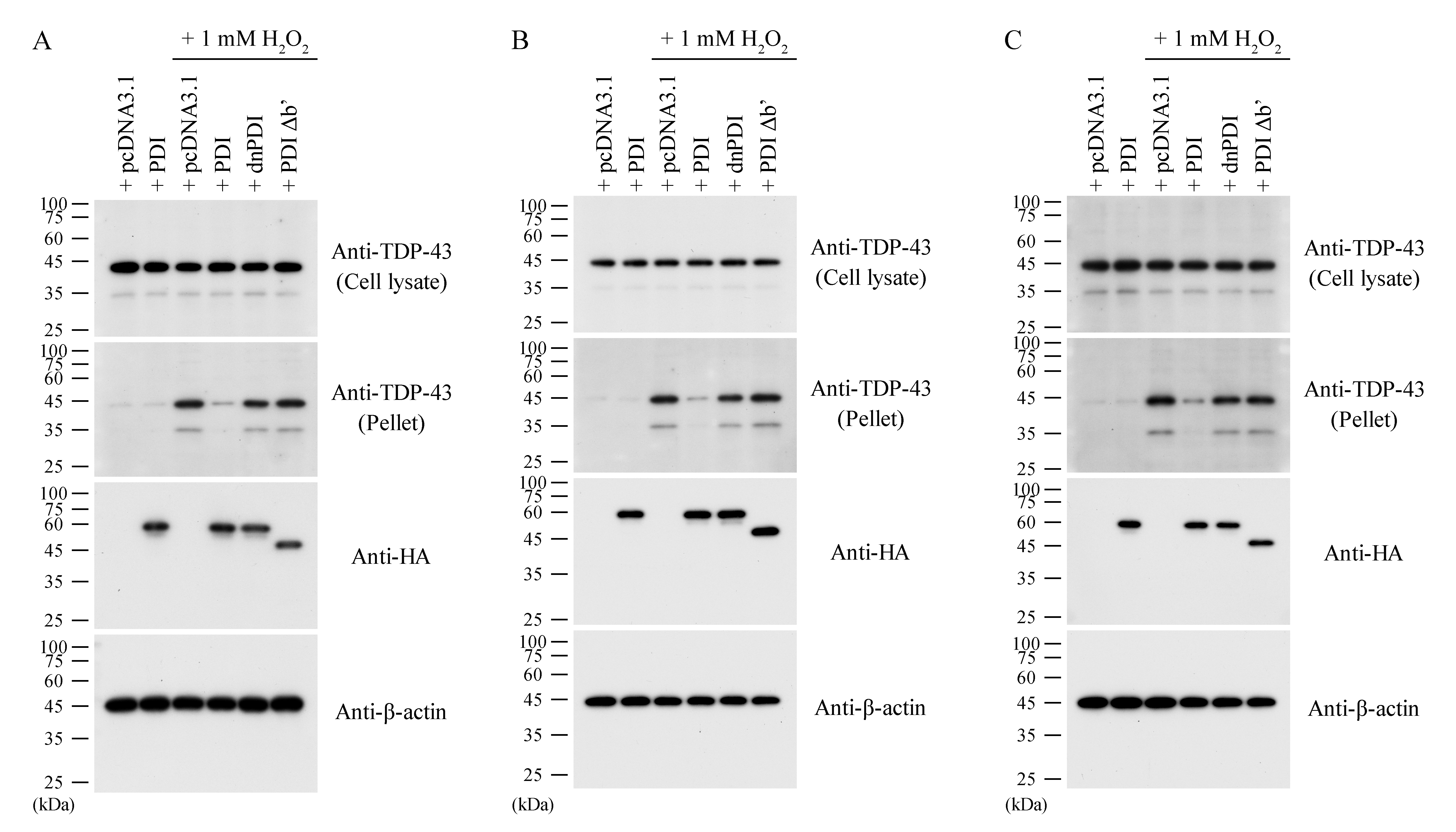

Supplement: Supplementary file 2 — Supporting File 2: advs75718‐sup‐0002‐Data.zip. [file ADVS-13-e16846-s001.zip › advs75718-sup-0002-Data/Fig6A_Uncropped_images.tif]

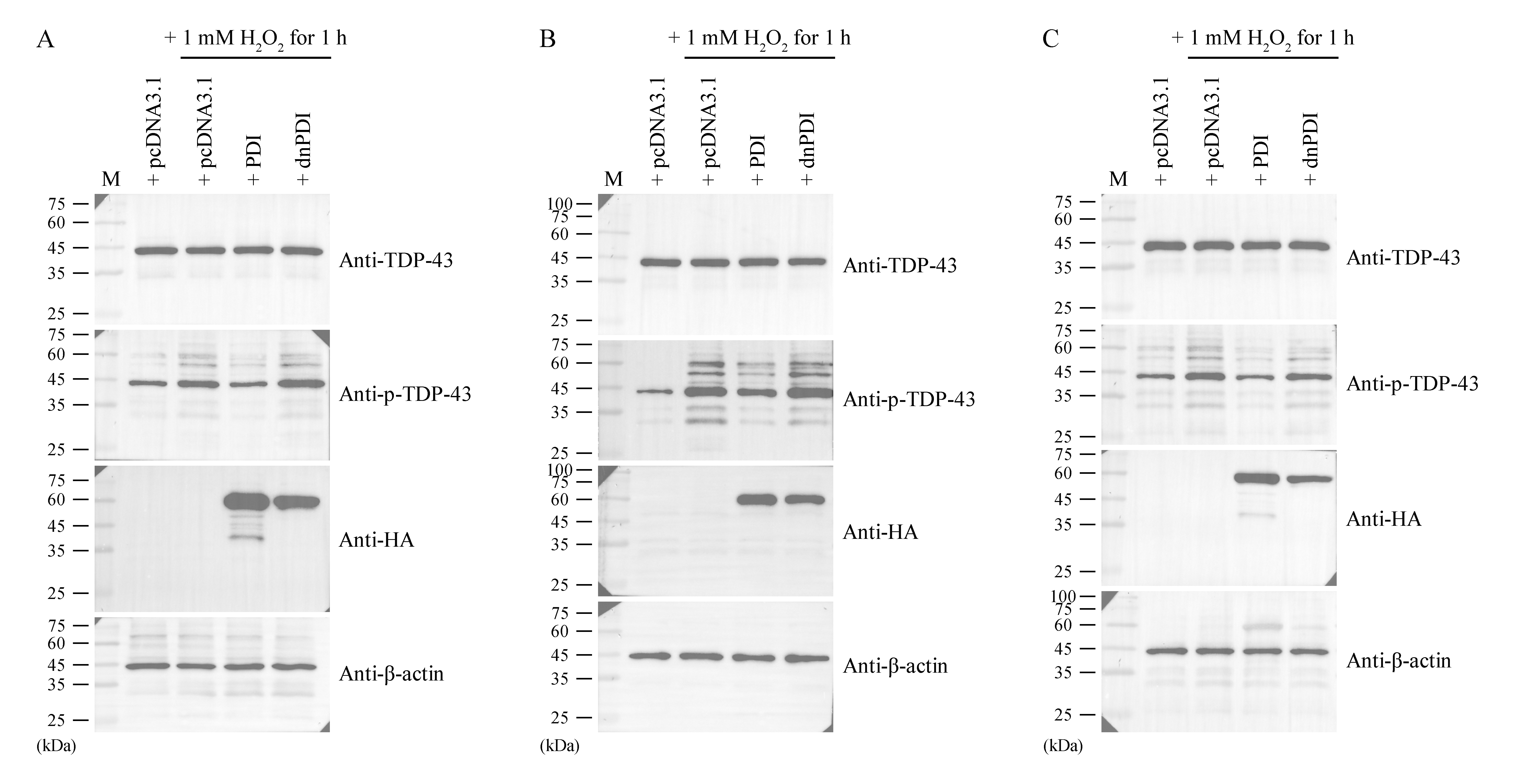

Supplement: Supplementary file 2 — Supporting File 2: advs75718‐sup‐0002‐Data.zip. [file ADVS-13-e16846-s001.zip › advs75718-sup-0002-Data/FigS11A_Uncropped_images.tif]

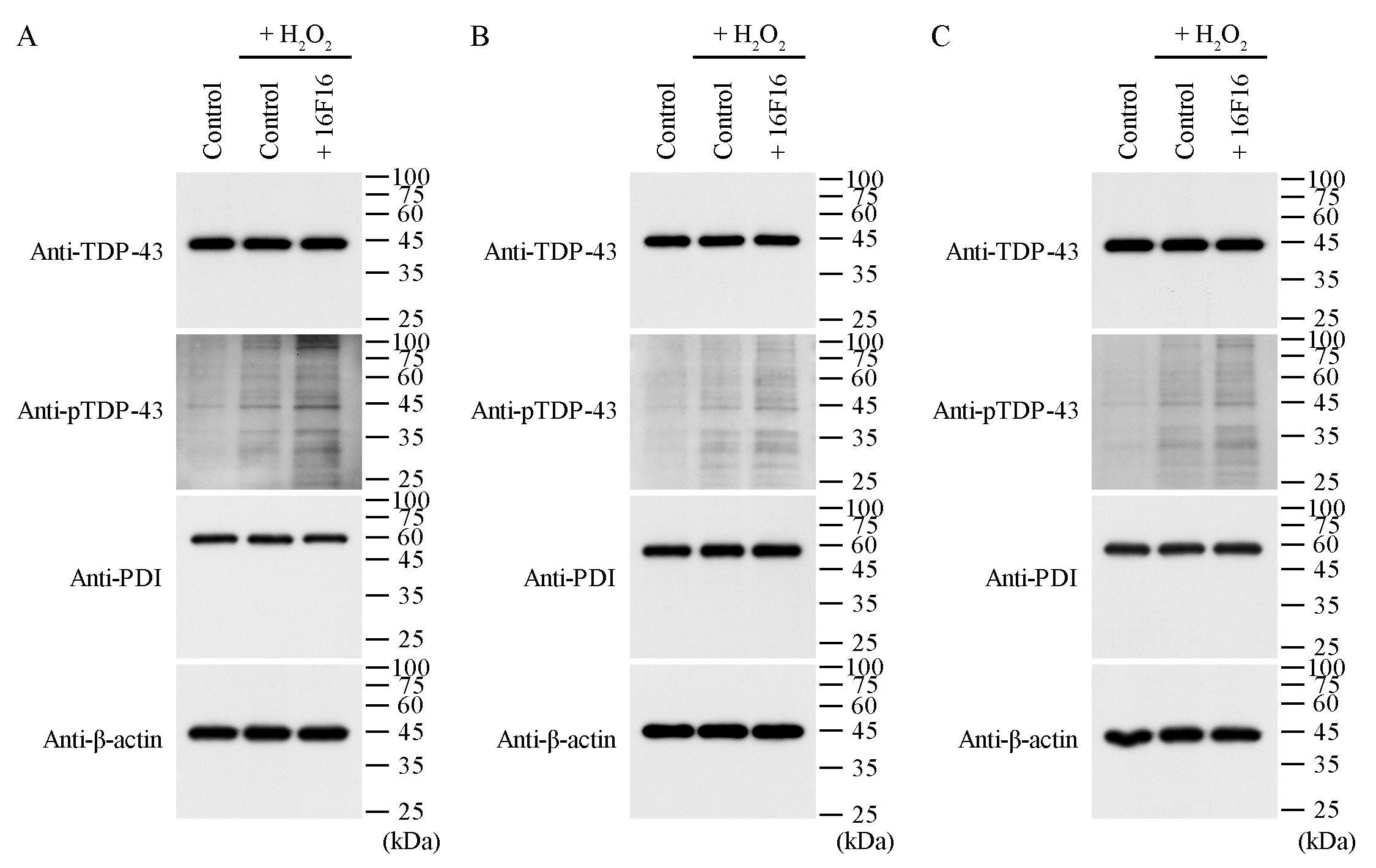

Supplement: Supplementary file 2 — Supporting File 2: advs75718‐sup‐0002‐Data.zip. [file ADVS-13-e16846-s001.zip › advs75718-sup-0002-Data/FigS11D_Uncropped_images.tif]

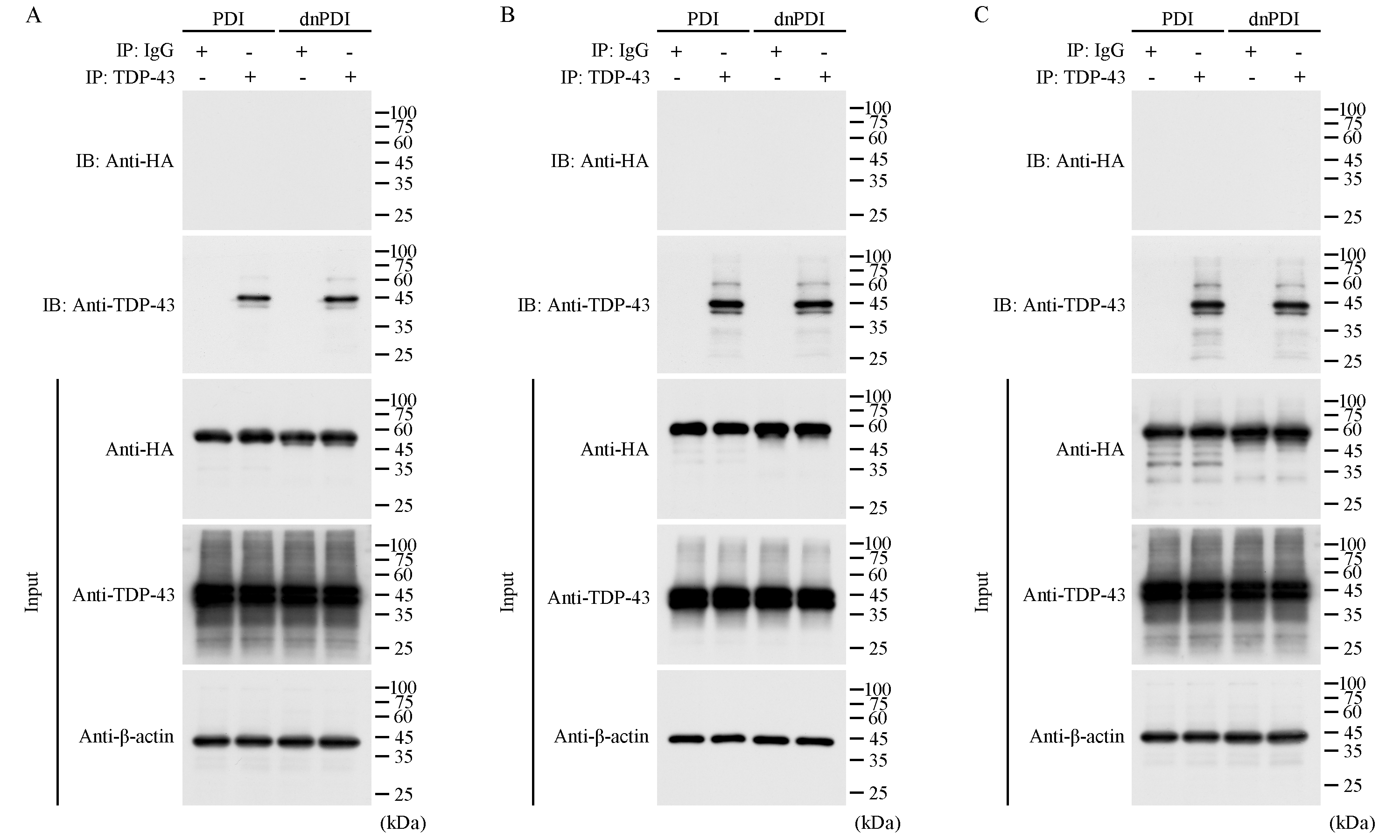

Supplement: Supplementary file 2 — Supporting File 2: advs75718‐sup‐0002‐Data.zip. [file ADVS-13-e16846-s001.zip › advs75718-sup-0002-Data/FigS1A_Uncropped_images.tif]

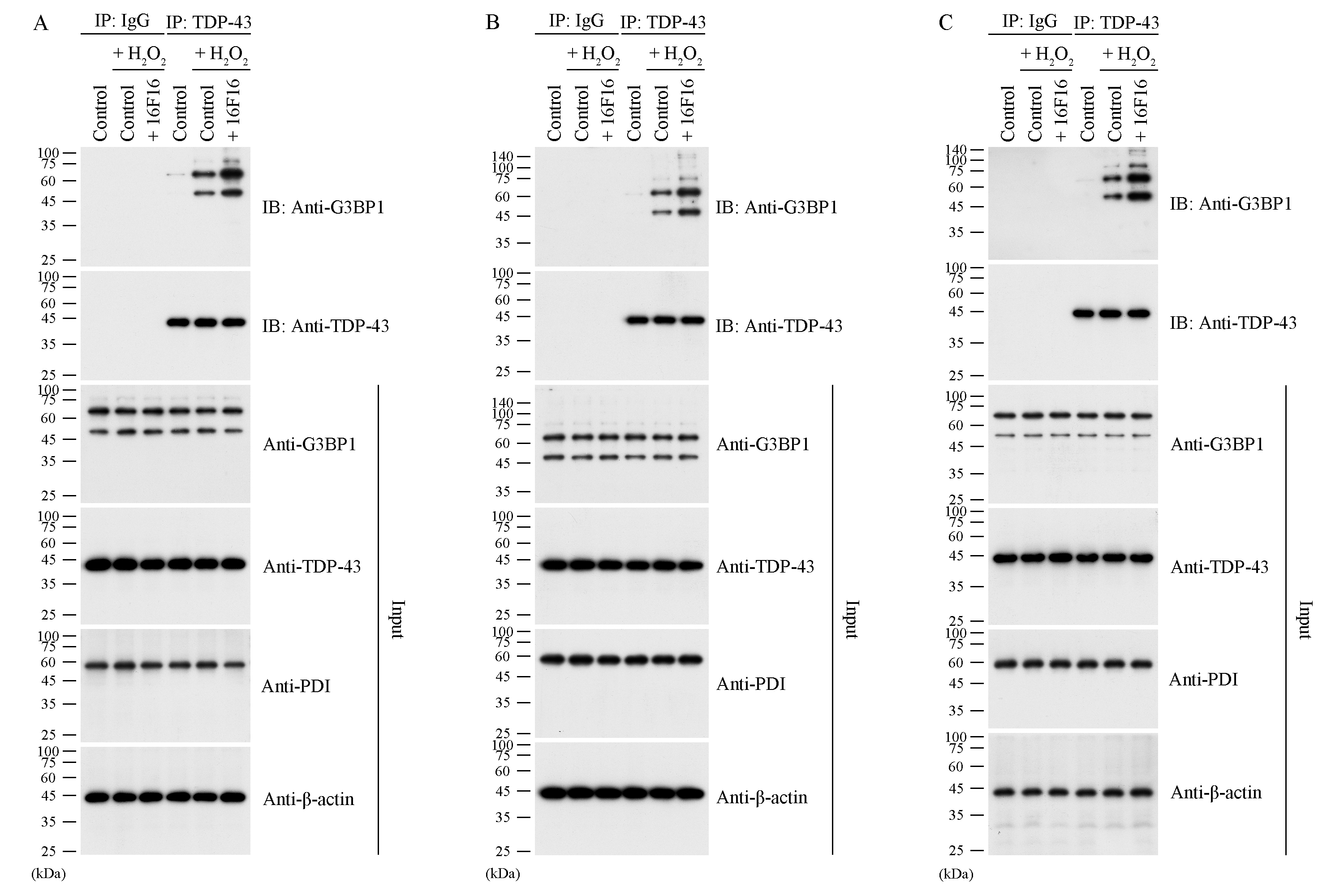

Supplement: Supplementary file 2 — Supporting File 2: advs75718‐sup‐0002‐Data.zip. [file ADVS-13-e16846-s001.zip › advs75718-sup-0002-Data/FigS9B_Uncropped_images.tif]
