# Supplementary material for: Protein Disulfide Isomerase Disassembles TDP‐43/G3BP1 Condensates and Antagonizes TDP‐43 Pathological Aggregates
Source: Adv Sci (Weinh). 2026 May 25;13(38):e16846. doi: 10.1002/advs.202516846 (PMC13336076; doi:10.1002/advs.202516846)
Supplement: Supplementary file 1 — Supporting File 1: advs75718‐sup‐0001‐SuppMat.doc. [file ADVS-13-e16846-s002.doc]

**Protein Disulfide Isomerase Disassembles TDP-43/G3BP1 Condensates and Antagonizes TDP-43 Pathological Aggregates**

Jia-Qi Liu ^1^, Hao Liu ^1^, Yu-Xuan Sun ^1^, Yuying Li ^2^, Xiangyi Liu ^3^, Li-Qiang Wang ^1^, Zhaofei Yang ^4^, Qi Fu ^5^, Xiaojiao Xu ^6^, Jie Chen ^1^, Yingshuang Zhang ^3^, Jun Zhou ^5^, Weidong Le ^6^, Mengchao Cui ^2,7^ and Yi Liang ^1,^*

Running Title: PDI suppresses TDP-43 phase separation in ALS

**Appendix A. Supplementary data**

**Table S1. Information for primers.**

| Primer name | Application | Sequence (5’ to 3’) |
| --- | --- | --- |
| S-PDI C53A | dnPDI vector | GCCCCTTGGGCTGGCCACTGCAAGG |
| A-PDI C53A | dnPDI vector | GCAGTGGCCAGCCCAAGGGGCATAG |
| S-PDI C56A | dnPDI vector | GTGGCCACGCCAAGGCTCTGGCCCC |
| A-PDI C56A | dnPDI vector | GAGCCTTGGCGTGGCCACACCAAGG |
| S-PDI C397A | dnPDI vector | CCCATGGGCTGGTCACTGCAAACAG |
| A-PDI C397A | dnPDI vector | CAGTGACCAGCCCATGGGGCATAGA |
| S-PDI C400A | dnPDI vector | GTGGTCACGCCAAACAGTTGGCTCC |
| A-PDI C400A | dnPDI vector | CAACTGTTTGGCGTGACCACACCAT |
| Actin-Fw | RT-qPCR | ACTCTTCCAGCCTTCCTTCC |
| Actin-Rv | RT-qPCR | CAATGCCAGGGTACATGGTG |
| TDP-43-Fw | RT-qPCR | AATTCTGCATGCCCCAGA |
| TDP-43-Rv | RT-qPCR | GAAGCATCTGTCTCATCCATTTT |
| P4HB-Fw | RT-qPCR | TTCAGGAATGGAGACACGGC |
| P4HB-Rv | RT-qPCR | TCCACGTCCTTGAAGAAGCC |
| UNC13A-Fw | RT-qPCR | GGACGTGTGGTACAACCTGG |
| UNC13A-Rv | RT-qPCR | GTGTACTGGACATGGTACGGG |
| UNC13A CE-Fw | RT-qPCR | CAAGCGAACTGACAAATCTGCCGTGTCG |
| UNC13A CE-Rv | RT-qPCR | GGCATCGTCACCCTTGGCATCTGG |
| TTBK1-Fw | RT-qPCR | ACACAGCCATCACCAGCAG |
| TTBK1-Rv | RT-qPCR | GCCAGGAGGTTGTTTGGGG |

**FIGURE S1** **|** TDP-43, PDI, and pathological stress conditions are all required for the interaction of TDP-43 with PDI in living cells. PDI includes wild-type PDI and its mutant dnPDI. (A) Co-IP assay to verify that TDP-43 did not bind to PDI in HEK-293T cells transiently co-transfected with TDP-43 and HA-tagged wild-type PDI or dnPDI treated with PBS. Anti-TDP-43 antibody-binding beads were used for co-IP experiments and then detected by western blotting using anti-HA, anti-TDP-43, and anti-β-actin antibodies. We have added IP bands of TDP-43 in the second row. (B−G) The negative controls of BiFC experiments. (B,C) BiFC assay to verify that TDP-43 did not bind to PDI in living cells treated with PBS. HEK-293T cells transiently expressing both TDP-43-GN172 and wild-type PDI-GC155 (B) or dnPDI-GC155 (C) constructs were treated with PBS. (D−G) TDP-43 and PDI are required for this putative interaction in living cells. HEK-293T cells transiently expressing both TDP-43-GN172 and GC155 constructs (D) and HEK-293T cells transiently expressing both GN172 and wild-type PDI-GC155 (E), dnPDI-GC155 (F), or GC155 (G) constructs were treated with 1 mM H_2_O_2_ for 1 h. The nuclei were stained with Hoechst 33342 (blue). (H,I) The vector-only control of BiFC experiments. Immunofluorescence images of HEK-293T cells transiently expressing TDP-43-GN172 treated with 1 mM H_2_O_2_ or PBS for 1 h, using the anti-FLAG antibody (red) or anti-GFP antibody (red)and staining with DAPI (blue). EGFP (green) was not detected in (B−G) and (H,I). Scale bars, 10 μm (B−I).

**FIGURE** **S2** **|** TDP-43 accumulates and exhibits overlapping distribution with PDI in the cytoplasm of the brain of ALS patients and AD-TDP patients. (A**−**H) Double immunofluorescence staining (anti-TDP-43 and anti-PDI) of paraffin brain sections from two patients with ALS (A and B, Cases 1 and 2) and six patients with AD-TDP (C−H, Cases 3 to 8). ALS patient one and ALS patient two (upper row) were sampled in the precentral gyrus, ALS patient two (upper row) were also sampled in the substantia nigra (bottom row), and the AD-TDP patients were sampled in the hippocampus and temporal lobe. (I−M) TDP-43 neither mislocalized to the cytoplasm nor colocalized with PDI in the brain of five controls. Double immunofluorescence staining (anti-TDP-43 and anti-PDI) of paraffin brain sections from three healthy controls (Controls 1 to 3) (I−K), two controls with Castleman disease and progressive supranuclear palsy (PSP), respectively (Controls 4 and 5) (L,M). The healthy controls and the control with PSP were sampled in the hippocampus and temporal lobe, and the control with Castleman disease was sampled in the precentral gyrus. Shown are nuclei stained with DAPI (blue); signals were detected with antibodies against TDP-43 (green) and PDI (red). The enlarged regions show 9-fold enlarged images from the merged images. Orange puncta in the merged images indicate that cytoplasmic TDP-43 and PDI signals exhibit overlapping distribution patterns in the brain of these patients (A**−**H). Scale bars, 20 μm. Line-scan analyses (right) show the spatial overlap between cytoplasmic TDP-43 (green) and PDI (red) signals of these patients (A**−**H) or the spatial overlap between TDP-43 (green) and PDI (red) signals of these controls (I−M). The fluorescence of each group was separately normalized using the average of the maximum green or red fluorescence values in the selected regions. (N) Pearson correlation coefficient (r) between TDP-43 and PDI in the brain of ALS patients and AD-TDP patients as well as in the brain of five controls. Data are presented as a scatter plot, showing the quantification of data from at least ten fields of vision per group. Black lines represent the mean of Pearson r value. Statistical analyses were performed using one-way ANOVA followed by Bonferroni multiple comparison tests, and different letters indicate significant differences at the level of *p* < 0.05.

**FIGURE S3** **|** PDI is partly mislocalized from the ER to the cytosol in the brain of ALS patients and AD-TDP patients. (A−H) Double immunofluorescence staining (anti-ER and anti-PDI) of paraffin brain sections from two patients with ALS (A and B, Cases 1 and 2) and six patients with AD-TDP (C−H, Cases 3 to 8). (I−K) Double immunofluorescence staining (anti-ER and anti-PDI) of paraffin brain sections from three healthy controls (Controls 1 to 3). Shown are nuclei stained with DAPI (blue); signals were detected with antibodies against ER (green) and PDI (red). The enlarged regions show 9-fold enlarged images from the merged images. Red puncta in the merged images indicate that PDI is partly mislocalized from the ER to the cytosol (A−H). Scale bars, 20 μm. Line-scan analyses (right) show the spatial overlap between ER (green) and PDI (red) signals of these patients (A−H) or the spatial overlap between ER (green) and PDI (red) signals of these controls (I−K). The fluorescence of each group was separately normalized using the average of the maximum green or red fluorescence values in the selected regions. (L) Pearson correlation coefficient (r) between ER and PDI in the brain of ALS patients and AD-TDP patients as well as in the brain of three healthy controls. Data are presented as a scatter plot, showing the quantification of data from at least ten fields of vision per group. Black lines represent the mean of Pearson r value. Statistical analyses were performed using one-way ANOVA followed by Bonferroni multiple comparison tests, and different letters indicate significant differences at the level of *p* < 0.05.

**FIGURE S4** **|** Non-specific staining of human brain samples (the secondary antibody control). (A−H) Double immunofluorescence staining of paraffin brain sections from two patients with ALS (A and B, Cases 1 and 2) and six patients with AD-TDP (C−H, Cases 3 to 8). (I−M) Double immunofluorescence staining of paraffin brain sections from three healthy controls (Controls 1 to 3) (I−K), two controls with Castleman disease and progressive supranuclear palsy (PSP), respectively (Controls 4 and 5) (L,M). Shown are nuclei stained with DAPI (blue); signals were detected with antibodies against rabbit IgG (green) and mouse IgG (red). Scale bars, 20 μm.

**FIGURE S5** **|** (A,B) A droplet fusion event occurs in the regions of different time slots. During the first 20 min after droplet initiation, two small liquid condensates of TDP-43 rapidly fused into one larger liquid droplet within 2 seconds. (C) After 60 min of incubation, two small liquid condensates of TDP-43 slowly fused into one larger liquid droplet within 60 seconds. Fluorescence images of in vitro phase-separated droplets (red) of 10 μM TAMRA-labeled TDP-43 incubated with Tris-HCl buffer (pH 8.0) containing 10% PEG 3350 on ice for the indicated time. Scale bars, 2 μm.

**FIGURE S6** **|** Wild-type PDI, dnPDI, SNO-PDI, or bovine serum albumin (BSA) alone did not form liquid droplets. (A) Characterization of S-nitrosylated PDI by fluorescence spectroscopy. Tris-HCl buffer (pH 8.0) (control) and Tris-HCl buffer (pH 8.0) containing wild-type PDI or SNO-PDI were incubated with a solution containing 100 μM DAN and 100 μM CuSO_4_ in the dark at room temperature for 30 min. S-nitrosylated PDI was then assessed by the release of NO, causing the conversion of DAN into the fluorescent compound NAT. The fluorescence intensity of NAT in the aforementioned samples (open red circles shown in scatter plots) measured using excitation at 375 nm and emission at 450 nm is expressed as the mean ± SD (with error bars) of values obtained in four independent experiments. SNO-PDI, *p* = 0.00000000088. Statistical analyses were performed using two-sided Student’s *t* tests. Values of *p* < 0.05 indicate statistically significant differences. The following notation was used throughout: **p* < 0.05, ***p* < 0.01, ****p* < 0.001, and *****p* < 0.0001 relative to the control (wild-type PDI). (B−E) Fluorescence images of Tris buffer containing 10 μM FITC-labeled wild-type PDI (B), dnPDI (C), SNO-PDI (D), or BSA (E) (green) and 10% PEG 3350 on ice as four controls for the regulation of TDP-43 LLPS by wild-type PDI. Scale bar, 10 μm.

**FIGURE S7** **|** Wild-type PDI enhances fluorescence recovery and negatively modulates phase separation of TDP-43. (A−D) FRAP analysis of selected liquid droplets of 10 μM TAMRA-labeled TDP-43 incubated with Tris-HCl buffer (pH 8.0) containing 10% PEG 3350 (A), 10 μM wild-type PDI and 10% PEG 3350 (B), 10 μM dnPDI and 10% PEG 3350 (C), or 10 μM SNO-PDI and 10% PEG 3350 (D) on ice before (prebleach), during (0 s), and after photobleaching (20, 50, 100, and 200 s, respectively). The internal photobleaching is marked by a red square. Scale bar, 5 μm. (E) Normalized kinetics of fluorescence recovery data of TDP-43 (blue circle), TDP-43 + wild-type PDI (red square), TDP-43 + dnPDI (olive circle), and TDP-43 + SNO-PDI (magenta square) obtained from FRAP intensity. The normalized fluorescence intensity is expressed as the mean ± SD (with error bars) of values obtained in three independent experiments. The solid lines show the best single exponential fit for the fluorescence intensity-time curves. (F) The fluorescence recovery percentage of TDP-43 condensates (open red circles shown in scatter plots) is expressed as the mean ± SD (with error bars) of values obtained in three biologically independent experiments. TDP-43 + wild-type PDI, *p* = 0.00045; TDP-43 + dnPDI, *p* = 0.681 or 0.00359 (red); and TDP-43 + SNO-PDI, *p* = 0.139 or 0.0358 (red). Statistical analyses were performed using two-sided Student’s *t* tests. Values of *p* < 0.05 indicate statistically significant differences. The following notation was used throughout: **p* < 0.05, ***p* < 0.01, and ****p* < 0.001 relative to the control (fluorescence recovery for TDP-43 or TDP-43 incubated with wild-type PDI). n.s., not significant.

**FIGURE S8** **|** Wild-type PDI blocks the mislocalization of TDP-43 to the cytoplasm induced by H_2_O_2_. (A) Western blot for endogenous TDP-43 in the cytoplasmic and nuclear protein fractions and the corresponding cell lysates from N2a cells transiently expressing HA-tagged wild-type PDI or HA-tagged dnPDI or HA-tagged PDI Δb’ mutant treated with 1 mM H_2_O_2_ for 1 h. β-actin and histone H3 served as markers for the cytosolic and nuclear fractions, respectively, and the empty vector pcDNA3.1 served as a control. (B) The normalized amount of endogenous TDP-43 in the cytoplasm (left) and nucleus (right) of the aforementioned cells (open red circles shown in scatter plots) is expressed as the mean ± SD (with error bars) of values obtained in three independent experiments. Wild-type PDI blocks but dnPDI and PDI Δb’ mutant do not block the mislocalization of TDP-43 to the cytoplasm induced by H_2_O_2_. (C,D) Immunofluorescence images of N2a cells transiently expressing both EGFP-TDP-43 and the empty vector pcDNA3.1 (control) and transiently expressing both EGFP-TDP-43 and the empty vector pcDNA3.1 treated with 1 mM H_2_O_2_ for 20 min (C) and 1 h (D) (control + H_2_O_2_), respectively; transiently expressing both EGFP-TDP-43 and HA-tagged wild-type PDI treated with 1 mM H_2_O_2_ for 20 min (C) and 1 h (D) (wild-type PDI + H_2_O_2_), respectively; and transiently expressing both EGFP-TDP-43 and HA-tagged dnPDI also treated with 1 mM H_2_O_2_ for 20 min (C) and 1 h (D) (dnPDI + H_2_O_2_), respectively, using an anti-HA antibody (red) and staining with DAPI (blue). EGFP-TDP-43 (green) was detected. Scale bars: 10 μm. (E) The relative number of cells with nuclear-localized TDP-43 (normal/total) (percent) in the aforementioned cells (open red circles shown in scatter plots) is expressed as the mean ± SD (with error bars) of values obtained in three biologically independent experiments, and the quantification of data is from at least five fields of vision per group. (B,E) Statistical analyses were performed using one-way ANOVA followed by Bonferroni multiple comparison tests, and different letters indicate significant differences at the level of *p* < 0.05.

**FIGURE S9** **|** Wild-type PDI also disassembles TDP-43/G3BP1 condensates in HEK-293T cells. (A) Immunofluorescence images of HEK-293T cells transiently expressing both EGFP-TDP-43 and pcDNA3.1 treated with PBS for 1 h (control), transiently expressing both EGFP-TDP-43 and HA-tagged wild-type PDI treated with PBS for 1 h (PDI), transiently expressing both EGFP-TDP-43 and pcDNA3.1 treated with 1 mM H_2_O_2_ for 1 h (control + H_2_O_2_), transiently expressing both EGFP-TDP-43 and HA-tagged wild-type PDI treated with 1 mM H_2_O_2_ for 1 h (PDI + H_2_O_2_), transiently expressing both EGFP-TDP-43 and HA-tagged dnPDI treated with 1 mM H_2_O_2_ for 1 h (dnPDI + H_2_O_2_), and transiently expressing both EGFP-TDP-43 and HA-tagged PDI Δb’ mutant treated with 1 mM H_2_O_2_ for 1 h (PDI Δb’ + H_2_O_2_), using anti-HA (red) and anti-G3BP1 (magenta) antibodies and staining with DAPI (blue). EGFP-TDP-43 (green) was detected. The white arrows indicate colocalization of TDP-43 condensates and endogenous G3BP1 in HEK-293T cells. (B−D) Basal PDI disassembles TDP-43/G3BP1 condensates in HEK-293T cells by significantly decreasing the interaction of cytoplasmic TDP-43 with G3BP1 induced by H_2_O_2_. (B) Co-IP assay to verify the interaction of endogenous TDP-43 with endogenous G3BP1 in HEK-293T cells treated with PBS for 1 h (control), treated with 1 mM H_2_O_2_ for 1 h (control + H_2_O_2_), and treated with 5 μM PDI inhibitor 16F16 for 16 h and then 1 mM H_2_O_2_ for 1 h (control + 16F16 + H_2_O_2_). Anti-TDP-43 antibody-binding beads were used for co-IP experiments and then detected by western blotting using anti-G3BP1, anti-TDP-43, anti-PDI, and anti-β-actin antibodies. (C) The normalized amount of immunoprecipitated G3BP1 in the aforementioned cells (open red circles shown in scatter plots) is expressed as the mean ± SD (with error bars) of values obtained in three independent experiments. Control + H_2_O_2_, *p* = 0.0000072; and control + 16F16 + H_2_O_2_, *p* = 0.0000096 or 0.00038 (red). (D) Basal PDI disassembles TDP-43/G3BP1 condensates in HEK-293T cells. Immunofluorescence images of HEK-293T cells transiently expressing EGFP-TDP-43 treated with PBS for 1 h (control), transiently expressing EGFP-TDP-43 treated with 1 mM H_2_O_2_ for 1 h (control + H_2_O_2_), and transiently expressing EGFP-TDP-43 treated with 5 μM PDI inhibitor 16F16 for 16 h and then 1 mM H_2_O_2_ for 1 h (control + 16F16 + H_2_O_2_), using anti-PDI (red) and anti-G3BP1 (magenta) antibodies and staining with DAPI (blue). EGFP-TDP-43 (green) was detected. The white arrows indicate colocalization of TDP-43 condensates and endogenous G3BP1 in HEK-293T cells. Scale bars: 10 μm (A,D). Statistical analyses were performed using two-sided Student’s *t*-test. Values of *p* < 0.05 indicate statistically significant differences. The following notation is used throughout: **p* < 0.05; ***p* < 0.01; ****p* < 0.001; and *****p* < 0.0001 relative to control or control + H_2_O_2_.

**Figure S10** **|** Wild-type PDI slows down phase separation of TDP-43 through free cysteine residues of the protein. (A) TDP-43 condensate formation regulated by PDI. Fluorescence images of 10 μM recombinant TDP-43 labeled by TAMRA (red) and incubated with Tris-HCl buffer containing wild-type PDI and dnPDI at 10 μM and 10% PEG 3350 on ice, and of 10 μM recombinant TDP-43-iodoacetamide (IAM) also labeled by TAMRA (red) and incubated with Tris-HCl buffer containing 10 μM wild-type PDI and 10% PEG 3350 on ice. Scale bar, 10 μm. (B) Characterization of free cysteine thiols in TDP-43 and TDP-43-IAM. The normalized amount of free cysteine thiols in TDP-43 and TDP-43-IAM (open red circles shown in scatter plots) is expressed as the mean ± SD (with error bars) of values obtained in three independent experiments. TDP-43-IAM, *p* = 0.000073. Statistical analyses were performed using two-sided Student’s *t* tests. Values of *p* < 0.05 indicate statistically significant differences. The following notation was used throughout: **p* < 0.05, ***p* < 0.01, ****p* < 0.001, and *****p* < 0.0001 relative to the control (TDP-43). (C) Scatter plot analyzing both the area of droplets (TDP-43 condensates, μm^2^) coacervated with wild-type PDI (pale blue), dnPDI (dark blue), or without PDI (control, very light pale blue), and the area of droplets (TDP-43-IAM condensates, μm^2^) coacervated with wild-type PDI (blue) or without PDI (control, pale gray-blue), and showing the quantification of data from *n* = 1000 condensates examined over 3 independent experiments. Black lines represent the mean of area of droplets. Statistical analyses were performed using one-way ANOVA followed by Bonferroni multiple comparison tests, and different letters indicate significant differences at the level of *p* < 0.05.

**FIGURE S11** **|** Wild-type PDI antagonizes pathological phosphorylation of TDP-43 induced by H_2_O_2_ by inhibiting oxidative stress-induced elevation of transcript expression levels of the *TTBK1* gene in HEK-293T cells. (A) Western blot for phosphorylated endogenous TDP-43 (pTDP-43) in HEK-293T cells transiently expressing empty vector pcDNA3.1 treated with PBS for 1 h, transiently expressing pcDNA3.1 treated with 1 mM H_2_O_2_ for 1 h, and transiently expressing HA-tagged wild-type PDI or HA-tagged dnPDI treated with 1 mM H_2_O_2_ for 1 h. The cell lysates from the aforementioned cells were probed by anti-TDP-43, anti-pTDP-43 (pSer409/410), anti-HA, and anti-β-actin antibodies. (B) The normalized amount of pTDP-43 in the aforementioned cells (open red circles shown in scatter plots) is expressed as the mean ± SD (with error bars) of values obtained in three independent experiments. Wild-type PDI + H_2_O_2_, *p* = 0.00027; and dnPDI + H_2_O_2_, *p* = 0.977 or 0.00095 (red). (C) Immunofluorescence imaging of HEK-293T cells transiently expressing both EGFP-TDP-43 and pcDNA3.1 treated with PBS for 1 h (control), transiently expressing both EGFP-TDP-43 and HA-tagged wild-type PDI treated with PBS for 1 h (PDI), transiently expressing both EGFP-TDP-43 and pcDNA3.1 treated with 1 mM H_2_O_2_ for 1 h (control + H_2_O_2_), transiently expressing both EGFP-TDP-43 and HA-tagged wild-type PDI treated with 1 mM H_2_O_2_ for 1 h (PDI + H_2_O_2_), transiently expressing both EGFP-TDP-43 and HA-tagged dnPDI treated with 1 mM H_2_O_2_ for 1 h (dnPDI + H_2_O_2_), and transiently expressing both EGFP-TDP-43 and HA-tagged PDI Δb’ mutant also treated with 1 mM H_2_O_2_ for 1 h (PDI Δb’ + H_2_O_2_), using antibodies against HA (red) and pTDP-43 (magenta) and staining with DAPI (blue). EGFP-TDP-43 (green) was observed. (D−F) Basal PDI antagonizes pathological phosphorylation of TDP-43 induced by H_2_O_2_ in HEK-293T cells. (D) Western blot for phosphorylated endogenous TDP-43 (pTDP-43) in HEK-293T cells treated with PBS for 1 h (control), treated with 1 mM H_2_O_2_ for 1 h (control + H_2_O_2_), and treated with 5 μM PDI inhibitor 16F16 for 16 h and then 1 mM H_2_O_2_ for 1 h (control + 16F16 + H_2_O_2_). The cell lysates from the aforementioned cells were probed by anti-TDP-43, anti-pTDP-43 (pSer409/410) (the pTDP-43 Proteintech 80007-1 monoclonal antibody), anti-PDI, and anti-β-actin antibodies. (E) The normalized amount of pTDP-43 in the aforementioned cells (open red circles shown in scatter plots) is expressed as the mean ± SD (with error bars) of values obtained in three independent experiments. Control + H_2_O_2_, *p* = 0.00015; and control + 16F16 + H_2_O_2_, *p* = 0.000060 or 0.00076 (red). Statistical analyses were performed using two-sided Student’s *t*-test. Values of *p* < 0.05 indicate statistically significant differences. The following notation is used throughout: **p* < 0.05; ***p* < 0.01; and ****p* < 0.001 relative to control (pcDNA3.1 + H_2_O_2_ or wild-type PDI + H_2_O_2_). n.s., not significant (B). The following notation is used throughout: **p* < 0.05; ***p* < 0.01; ****p* < 0.001; and *****p* < 0.0001 relative to control or control + H_2_O_2_ (E). (F) Immunofluorescence imaging of the same HEK-293T cells as in (D), except for HEK-293T cells transiently expressing EGFP-TDP-43, using antibodies against PDI (red) and pTDP-43 (magenta) and staining with DAPI (blue). EGFP-TDP-43 (green) was observed. Scale bars: 10 μm (C,F). (G) RT-qPCR experiments demonstrated that wild-type PDI strongly inhibited H_2_O_2_-induced elevation of transcript expression levels of the *TTBK1* gene in HEK-293T cells. Relative transcript expression levels of the human *TARDBP* gene, the human *P4HB* gene, and the *TTBK1* gene associated with pathological phosphorylation of TDP-43 in HEK-293T cells transiently expressing the empty vector pcDNA3.1 treated with PBS (control) or 1 mM H_2_O_2_ (control + H_2_O_2_) for 1 h, transiently expressing wild-type PDI treated with PBS (PDI) or 1 mM H_2_O_2_ (PDI + H_2_O_2_) for 1 h, and transiently expressing dnPDI (dnPDI + H_2_O_2_) or PDI Δb’ mutant (PDI Δb’ + H_2_O_2_) also treated with 1 mM H_2_O_2_ for 1 h. The relative transcript expression levels of the three genes in the aforementioned cells are expressed as the mean ± SD (with error bars) of values obtained in three biologically independent experiments. Statistical analyses were performed using one-way ANOVA followed by Bonferroni multiple comparison tests, and different letters indicate significant differences at the level of *p* < 0.05.
